# Construction of 2DE Patterns of Plasma Proteins: Aspect of Potential Tumor Markers

**DOI:** 10.3390/ijms231911113

**Published:** 2022-09-21

**Authors:** Stanislav Naryzhny, Natalia Ronzhina, Elena Zorina, Fedor Kabachenko, Nikolay Klopov, Victor Zgoda

**Affiliations:** 1 Institute of Biomedical Chemistry, Pogodinskaya, 10, 119121 Moscow, Russia; 2 Petersburg Institute of Nuclear Physics (PNPI) of National Research Center “Kurchatov Institute”, 188300 Gatchina, Russia; 3 Institute of Biomedical Systems and Biotechnology, Peter the Great St. Petersburg Polytechnic University, 195251 St. Petersburg, Russia

**Keywords:** plasma, biomarker, proteomics, 2DE, proteoform, pattern

## Abstract

The use of tumor markers aids in the early detection of cancer recurrence and prognosis. There is a hope that they might also be useful in screening tests for the early detection of cancer. Here, the question of finding ideal tumor markers, which should be sensitive, specific, and reliable, is an acute issue. Human plasma is one of the most popular samples as it is commonly collected in the clinic and provides noninvasive, rapid analysis for any type of disease including cancer. Many efforts have been applied in searching for “ideal” tumor markers, digging very deep into plasma proteomes. The situation in this area can be improved in two ways—by attempting to find an ideal single tumor marker or by generating panels of different markers. In both cases, proteomics certainly plays a major role. There is a line of evidence that the most abundant, so-called “classical plasma proteins”, may be used to generate a tumor biomarker profile. To be comprehensive these profiles should have information not only about protein levels but also proteoform distribution for each protein. Initially, the profile of these proteins in norm should be generated. In our work, we collected bibliographic information about the connection of cancers with levels of “classical plasma proteins”. Additionally, we presented the proteoform profiles (2DE patterns) of these proteins in norm generated by two-dimensional electrophoresis with mass spectrometry and immunodetection. As a next step, similar profiles representing protein perturbations in plasma produced in the case of different cancers will be generated. Additionally, based on this information, different test systems can be developed.

## 1. Introduction

In a broad sense, tumor biomarkers are components that are either produced directly or indirectly because of a tumor. Moreover, these biomarkers can be common cellular products that are overproduced by cancer cells or the products of genes that are expressed only during malignant transformation. Thus, a tumor marker that is present in significant quantities indicates the presence of cancer. The marker can be present inside the tumor or enter the bloodstream [1,2]. This point is fundamentally important, as it allows the noninvasive examination and treatment of patients with various malignant neoplasms. The list of biochemical tumor markers known today is large [2]. Although some of these biomarkers have been successfully used in treatment, none of them fully satisfy the so-called “ideal marker”, which should be highly sensitive, specific, reliable with high predictive value, and correlate with the stages of tumor development [3].

Therefore, the search for new markers continues. Here, multi-omics technologies such as genomics, transcriptomics, and metabolomics are very important, but proteomics plays a central role since tumor biomarkers are mostly proteins. From a proteomic point of view, the search is based on a comparative analysis of proteomes. These proteomes are from body fluids (blood plasma, cerebrospinal fluid, saliva, urine, etc.) or tissues. Here, human plasma is one of the most popular clinical samples as it provides noninvasive, rapid analysis for any type of disease. A special human plasma proteome project (HPPP) project was initiated in 2002 (https://www.hupo.org/plasma-proteome-project accessed on 10 September 2022). Now, this initiative has achieved great success in plasma protein analysis (http://plasmaproteomedatabase.org/index.html accessed on 10 September 2022) [4,5]. One of the main advantages of using plasma samples is that only a minimally invasive assay such as a routine blood test analysis is required. To the greatest extent, this certainly concerns the hematopoietic organs (for instance, the major human plasma proteins are synthesized mostly in the liver), but also applies to other tissues, and even the brain, which is separated by the blood–brain barrier. It is expected that the blood plasma proteome should reflect, to varying degrees, changes in cellular proteomes caused by diseases. In recent years, biomarker selection guidelines have been developed [6,7,8,9,10]. Here, the classical proteomic approaches are used: two-dimensional electrophoresis (2DE), immunodetection, and mass spectrometry (MS), which have many methodological options that allow highly productive analysis individually or together in different combinations. Electrophoretic separation of plasma proteins offers a valuable diagnostic tool, as well as a way to monitor clinical progress [11]. MS measures, with high accuracy, the masses of peptides obtained by specific hydrolysis of proteins and is very specific. This approach was applied for detecting ovarian cancer (OC) based on just MS-spectra [12]. In addition, MS-based proteomics can detect and quantify protein variants—proteoforms [13]. Ideally, MS-based proteomics can analyze a whole proteome [14,15,16]. A rapid, robust, and reproducible shotgun plasma proteomics workflow was developed to produce “plasma proteome profiles” [14,17].

Accordingly, there are several directions for proteomics to develop ideal oncomarkers. First, we can go deep—find highly specific proteoforms/oncomarkers secreted by a tumor in low abundancy. Second, go wide—select, and analyze a panel of multiple proteins/oncomarkers. Third, combine these approaches. There are already some examples of generation from such panels [18]. This strategy can be applied to solid or liquid biopsies depending on the real situation. Here, the question arises about how to select these oncomarkers, as the concentration range of putative oncomarkers in plasma is very wide. The plasma proteome is the most complete version of the whole human proteome. In addition to the “classical plasma proteins”, it contains tissue proteins plus numerous individual immunoglobulins [19,20]. In clinics, a lot of information about the health state is obtained by analysis of blood proteins. Accordingly, in diagnosis and therapeutic monitoring, human plasma proteome analysis is a promising solution. The major protein, albumin, accounts for ~50% of the mass of all proteins. Nine proteins (IgG, apolipoprotein A1, apolipoprotein A2, transferrin, fibrinogen, haptoglobin, alpha1-antitrypsin, transthyretin) make up 40%, another 12 make up the next 9%, and the rest only 1%. Accordingly, it is common practice to remove the most abundant proteins (deplete) before deep proteomics analysis of plasma [21].

Two-dimensional electrophoresis analysis of human plasma proteins has a long history, where, possibly, the input of L. Anderson and N.G. Anderson is most impressive [22,23,24]. There are many publications where the 2DE image of plasma proteins was used as a specific profile for testing the cancer-related changes in the human body [25,26,27,28,29]. However, if we are going to decipher the whole panel of plasma proteins as a combined tumor biomarker, we need to obtain reliable data about every protein in connection to its response during the malignancy process. Previously, we started to collect information about the proteoform profiles of different cellular proteins into a database “2DE pattern” using our original approaches [30]. These approaches are time consuming and labor intensive but allow the presentation of panoramic data about different proteoforms and could be very useful in biomarker studies. Here, as a next step in searching for specific oncomarkers, we produced 2DE profiles for the human plasma proteins. The most abundant, “classical plasma proteins” were selected as they are detected reliably by common proteomics methods.

## 2. Results

**Figure 1:**
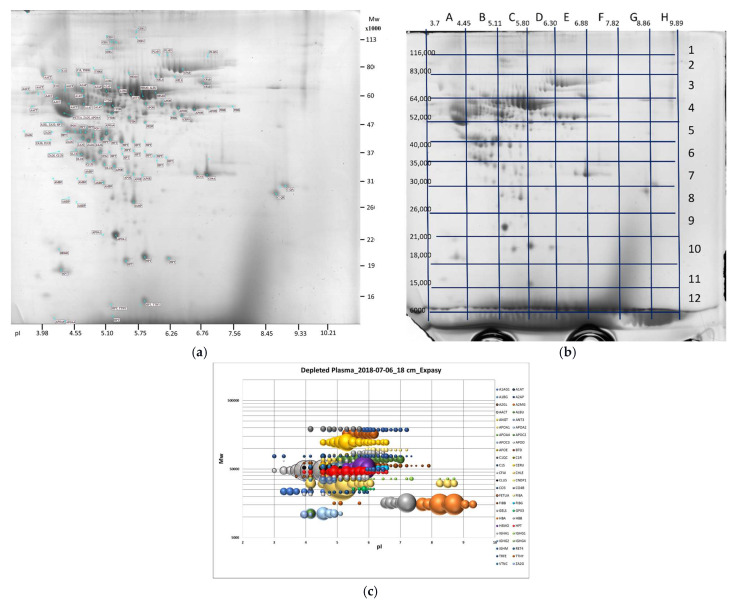
Two-dimensional electrophoresis image of depleted plasma proteins taken for the sectional analysis. (**a**) A classical annotated 2DE image of plasma proteins; (**b**) a sectional analysis of the gel presented in (**a**). The stained gel was divided into the sections with the predetermined coordinates, and each section was treated and analyzed by LC ESI-MS/MS (see Materials and Methods, Section 4.2, 2DE); (**c**) a semi-virtual 2DE of the major plasma proteins. The plasma proteins were separated by isoelectrophocusing (IEF), using the 18-cm Immobiline DryStrip 3–11 NL. The strip was cut to 36 equal sections, and each section was treated and analyzed by LC ESI-MS/MS (see Materials and Methods, Section 4.2, 2DE). According to the abundance (emPAI) of each protein in the sections, the graph was plotted. The ball size is proportional to the protein emPAI in each section.

In our study, using classical 2DE, sectional 2DE, and semi-virtual 2DE in combination with liquid chromatography–electrospray ionization tandem mass spectrometry (LC ESI-MS/MS), we generated 2DE patterns for the most abundant plasma proteins. In Figure 1, these 2DE images of plasma proteins are presented. The 2DE patterns of more than 100 reliable and confidently detected sets (Supplementary Tables S1 and S2) are presented in Supplementary Figure S1. We also collected data from the literature about the possibilities of using these plasma proteins as cancer biomarkers (Table 1) [20,31]. The detailed information about these proteins and the 2DE patterns of plasma proteins in norm generated in our experiments are described below and in the Supplementary File.

### 2.1. ALPHA-1-ACID GLYCOPROTEIN 1 (A1AG1_HUMAN)

The two-dimensional electrophoresis pattern of AGP-1 represents a chain of spots in the pI-range from 3 to 5 (Supplementary Figure S1). This pattern is well-represented in the SWISS-2DPAGE (pI/Mw: 4.11–4.29/43–46,000) [22]. Such a pattern is a result of heavy glycosylation (82 N-linked glycans at 6 sites), phosphorylation (2 sites), acetylation (2 sites), ubiquitylation (1 site) (https://www.uniprot.org/uniprot/P02763 accessed on 10 September 2022).

### 2.2. ALPHA-1-ACID GLYCOPROTEIN 2 (A1AG2_HUMAN)

The two-dimensional electrophoresis pattern of AGP-2 is very similar to AGP-1 (Supplementary Figure S1) and in SWISS-2DPAGE is overlapped with AGP-1 pattern [22]. AGP-2 can be glycosylated (99 N-linked glycans at 7 sites) and acetylated (1 site). (https://www.uniprot.org/uniprot/P19652 accessed on 10 September 2022).

### 2.3. ALPHA-1-ANTITRYPSIN (A1AT_HUMAN)

The two-dimensional electrophoresis pattern of Serpin A1 represents a chain of spots in the pI-range 4.5–5.1 (Supplementary Figure S1) [265] that is a result of multiple N-linked glycosylations (112 N-linked glycans at 5 sites, 5 O-linked glycans at 6 sites), phosphorylation (13 sites), and acetylation (17 sites) (https://www.phosphosite.org/ accessed on 10 September 2022) [266]. Accordingly, in SWISS-2DPAGE, 22 spots of serpin A1 are present (pI/Mw: 4.87–5.10/48–108,000) [22].

### 2.4. ALPHA-1B GLYCOPROTEIN (A1BG_HUMAN)

The two-dimensional electrophoresis pattern of alpha-1-B glycoprotein represents a long chain of spots in the pI-range 4–6 and Mw ~ 54,000 (Supplementary Figure S1) that is a result of heavy glycosylation (24 N-linked glycans at 4 sites, 2 O-linked glycans at 1 site) and phosphorylation (1 site) (https://glyconnect.expasy.org/browser/proteins/780 accessed on 10 September 2022). In SWISS-2DPAGE, alpha-1-B glycoprotein is represented as a chain of six spots (pI/Mw: 4.99–5.25/73–76,000) [22].

### 2.5. ALPHA-2-MACROGLOBULIN (A2MG_HUMAN)

The two-dimensional electrophoresis pattern of alpha-2-M represents a chain of heavy Mw spots (mostly in the pI-range 5.8–6.3) (Supplementary Figure S1). This pattern is also well-represented in the SWISS-2DPAGE https://world-2dpage.expasy.org/ accessed on 10 September 2022 [22]. Alpha-2-M has eight sites of O-GalNAc and eight sites of N-GlcNAc (https://glygen.org/protein/P01023#glycosylation accessed on 10 September 2022).

### 2.6. ALPHA-2-ANTIPLASMIN (A2AP_HUMAN)

The two-dimensional electrophoresis pattern of α2AP represents a chain of spots in the pI-range 4–6 with Mw ~ 50,000 (Supplementary Figure S1) that is a result of glycosylation (4 N-linked glycans at 1 site, 3 O-linked glycans at 4 sites), phosphorylation (7 sites), and ubiquitylation (2 sites) (https://glygen.org/protein/P08697#glycosylation accessed on 10 September 2022). In SWISS-2DPAGE, alpha-2-antiplasmin is represented as a chain of seven spots (pI/Mw: 4.87–5.17/66–74,000).

### 2.7. LEUCINE-RICH ALPHA-2-GLYCOPROTEIN (A2GL_HUMAN)

The two-dimensional electrophoresis pattern of LRG1 represents a chain of spots in the pI-range 3.5–5.0 with Mw ~ 40,000–50,000 (Supplementary Figure S1). This pattern is well-represented in the SWISS-2DPAGE https://world-2dpage.expasy.org/ accessed on 10 September 2022 and has a characteristic for multiple glycosylation profiles, where acidic spots have higher Mw [22]. LRG1 has at least six sites of glycosylation: one is O-GalNAc and five are N-GlcNAc (https://www.uniprot.org/uniprot/P02750 accessed on 10 September 2022).

### 2.8. ALPHA-1-ANTICHYMOTRYPSIN (AACT_HUMAN)

The two-dimensional electrophoresis pattern of ACT represents a chain of spots in the pI-range 4.0–5.0 and Mw 50–60,000 (Supplementary Figure S1). This pattern is also well-represented in the SWISS-2DPAGE, where two chains (20 spots) of both ACT forms are presented [22]. ACT has seven sites of N-GlcNAc and four sites of O-GalNAc (https://glygen.org/protein/P01011#glycosylation accessed on 10 September 2022).

### 2.9. ADIPONECTIN (ADIPO_HUMAN)

The two-dimensional electrophoresis pattern of adiponectin represents a chain of spots in the pI-range 5.0–5.5 and Mw 26,000 (Supplementary Figure S1). There are six sites of O-linked glycosylation and two sites of phosphorylation in adiponectin (https://glygen.org/protein/Q15848#glycosylation accessed on 10 September 2022).

### 2.10. AFAMIN (AFAM_HUMAN)

The two-dimensional electrophoresis pattern of adiponectin represents a chain of spots in the pI-range 4.5–6.0 and Mw ~ 70,000 (Supplementary Figure S1). There are six sites of N-linked glycosylation in afamin, and more than 90% of the glycans are sialylated (https://glygen.org/protein/P43652#glycosylation accessed on 10 September 2022).

### 2.11. ALBUMIN (ALBU_HUMAN)

The two-dimensional electrophoresis pattern of albumin represents a chain of spots in the pI-range 5.5–6.5 and Mw ~ 70,000 (Supplementary Figure S1). Its pattern is also well-represented in the SWISS-2DPAGE [22]. Albumin can be modified by N-linked glycans at one site, 7 O-linked glycans at 11 sites, phosphorylated at multiple sites (at least 15), and acetylated (1 site) (https://glygen.org/protein/P02768#glycosylation accessed on 10 September 2022).

### 2.12. PROTEIN AMBP (AMBP_HUMAN)

The two-dimensional electrophoresis pattern of AMBP represents two groups of spots in the pI-range 4.0–6.5: alpha-1-microglobulin with Mw ~ 26,000 and inter-alpha-trypsin inhibitor light chain/bikunin that is assembled in a high Mw complex by a chondroitin-like glycosaminoglycan (GAG) cross-linking with Mw 120,000 (Supplementary Figure S1). In the SWISS-2DPAGE, AMBP is represented only by the chain of three alpha-1-microglobulin spots. AMBP can be glycosylated (31 N-Linked glycans at 2 sites, 7 O-Linked glycans at 3 sites), phosphorylated (3 sites), and acetylated (1 site) (https://www.phosphosite.org/ accessed on 10 September 2022).

### 2.13. ANGIOTENSINOGEN (ANGT_HUMAN)

The two-dimensional electrophoresis pattern of angiotensinogen represents a chain of spots in the pI-range 4.0–6.4 and Mw ~ 50,000 (Supplementary Figure S1). In the SWISS-2DPAGE, angiotensinogen is represented by one spot (pI/Mw: 5.07/58,973). It was reported that there were 20 N-linked glycans at 3 sites and 1 O-linked glycan (1 site) (https://glygen.org/protein/P01019#glycosylation accessed on 10 September 2022).

### 2.14. ANTITHROMBIN-III (ANT3_HUMAN)

The two-dimensional electrophoresis pattern of ATIII represents a chain of eight spots in the pI-range 4.5–6.0 and Mw ~ 50,000 (Supplementary Figure S1). In the SWISS-2DPAGE, only two spots are presented (pI/Mw: 5.20/58,973 and 5.27/58,653) for ATIII [22]. The protein can be glycosylated (24 N-linked glycans at 4 sites, 2 O-linked glycans at 1 site), phosphorylated (9 sites), and ubiquitinated (1 site) (https://www.phosphosite.org/ accessed on 10 September 2022).

### 2.15. APOLIPOPROTEIN A-I (APOA1_HUMAN)

The two-dimensional electrophoresis pattern of apoA-I represents a chain of spots in the pI-range 4.5–6.5 and Mw ~ 26,000 (Supplementary Figure S1). In the SWISS-2DPAGE, nine spots are presented (chain of five spots pI/Mw: 4.99–5.48/~23,000 and four spots with Mw ~ 8000–9000) [22]. The protein can be heavily phosphorylated (13 sites), acetylated (13 sites), ubiquitinated (7 sites), succinylated (3 sites), or glycosylated (2 sites) (https://www.phosphosite.org/ accessed on 10 September 2022).

### 2.16. APOLIPOPROTEIN A-II (APOA2_HUMAN)

The two-dimensional electrophoresis pattern of apoA-II represents a chain of spots in the pI-range 4.5–6.0 and Mw ~ 9000 (Supplementary Figure S1). In the SWISS-2DPAGE, two spots are presented (pI/Mw: 4.74/12,520 and 4.71/7250) [22]. ApoA-II can be glycosylated (3 O-linked glycans at 3 sites), phosphorylated (7 sites), acetylated (1 site), or succinylated (1 site).

### 2.17. APOLIPOPROTEIN A-IV (APOA4_HUMAN)

The two-dimensional electrophoresis pattern of apoA-IV represents a chain of spots in the pI-range 4.5–6.0 and Mw ~ 40,000 (Supplementary Figure S1). In the SWISS-2DPAGE, six spots are presented (pI/Mw: 5.05–5.10/~43,000 (3 spots), 5.11/21,945, and 4.87–4.97/9–10,000 (3 spots)). ApoA-IV can be glycosylated (1 O-linked glycan at 1 site), phosphorylated (7 sites), acetylated (9 site), or ubiquitinated (1 site) (https://www.phosphosite.org/ accessed on 10 September 2022).

### 2.18. APOLIPOPROTEIN B-100 (APOB_HUMAN)

The two-dimensional electrophoresis pattern of apo B-100 represents a long chain of spots in the pI-range 3.5–7.5 and heavy Mw > 120,000 (Supplementary Figure S1). Apo B-100 can be glycosylated (28 sites, 82 N-linked glycans at 16 sites, 1 O-linked glycan at 7 sites), phosphorylated (43 sites), acetylated (4 sites), or ubiquitinated (8 sites) (https://www.phosphosite.org/ accessed on 10 September 2022).

### 2.19. APOLIPOPROTEIN C-I (APOC1_HUMAN)

The two-dimensional electrophoresis pattern of apo-CI represents a chain of spots in the pI-range 7.8–8.5 and Mw ~ 6000 (Supplementary Figure S1). This protein can be acetylated (3 sites) and ubiquitinated (4 sites) (https://www.phosphosite.org/ accessed on 10 September 2022).

### 2.20. APOLIPOPROTEIN C-II (APOC2_HUMAN)

The two-dimensional electrophoresis pattern of apo-CII represents a chain of spots in the pI-range 4.8–5.2 and Mw ~ 8000 (Supplementary Figure S1). In the SWISS-2DPAGE, two spots for apo-CII are presented (pI/Mw: 4.51/9976 and 4.58/9248). This protein can be acetylated (5 sites), ubiquitinated (2 sites) (https://www.phosphosite.org/ accessed on 10 September 2022), and glycosylated (3 O-linked glycans at 4 sites) (https://www.glygen.org/protein/P02655 accessed on 10 September 2022).

### 2.21. APOLIPOPROTEIN C-III (APOC3_HUMAN)

The two-dimensional electrophoresis pattern of apoC-III represents a chain of spots in the pI-range 3.8–6.1 and Mw ~ 9000 (Supplementary Figure S1). In the SWISS-2DPAGE, only one spot for apoC-III is presented (pI/Mw: 4.63/8528). This protein can be phosphorylated (7 sites) acetylated (1 site), ubiquitinated (1 site) (https://www.phosphosite.org/ accessed on 10 September 2022), and glycosylated (4 O-linked glycans at 1 site) (https://www.glygen.org/protein/P02655 accessed on 10 September 2022).

### 2.22. APOLIPOPROTEIN D (APOD_HUMAN)

The two-dimensional electrophoresis pattern of apoD represents an unusual set of spots in the pI-range 3.5–6.5 and Mw from ~15,000 to ~26,000 and 80,000 (Supplementary Figure S1). In the SWISS-2DPAGE, a cluster of 12 spots for apoD is presented (pI/Mw: 4.44–4.78/27–32,000). ApoD can be heavily glycosylated (115 N-linked glycans at 2 sites, 1 O-linked glycan at 1 site) and phosphorylated (1 site) (https://www.uniprot.org/uniprotkb/P05090/entry accessed on 10 September 2022).

### 2.23. APOLIPOPROTEIN E (APOE_HUMAN)

The two-dimensional electrophoresis pattern of apoE represents a chain of spots in the pI-range 4.5–6.5 and Mw ~ 35,000 (Supplementary Figure S1). In the SWISS-2DPAGE, there is a chain of three spots (pI/Mw: 5.24–5.49/34–35,320). ApoE can be glycosylated (6 O-linked glycans at 6 sites), phosphorylated (9 sites), acetylated (1 site), and ubiquitinated (5 sites) (https://www.phosphosite.org/ accessed on 10 September 2022).

### 2.24. APOLIPOPROTEIN F (APOF_HUMAN)

The two-dimensional electrophoresis pattern of apo-F represents a set of spots in the pI-range 3.5–4.2 and Mw from ~15,000 to ~32,000 (Supplementary Figure S1). ApoF can be glycosylated (16 N-linked glycans at 3 sites, 6 O-linked glycans at 5 sites), phosphorylated (1 site), and ubiquitinated (2 sites) (https://www.phosphosite.org/ accessed on 10 September 2022).

### 2.25. BETA-2-GLYCOPROTEIN 1 (APOH_HUMAN)

The two-dimensional electrophoresis pattern of apo-H represents a chain of spots in the pI-range 6.2–8.4 and Mw ~ 52,000 (Supplementary Figure S1), which is much higher than the theoretical one because of heavy glycosylation (85 N-linked annotations at 4 sites and 3 O-linked annotations at 3 sites) (https://glygen.org/protein/P02749#glycosylation accessed on 10 September 2022).

### 2.26. APOLIPOPROTEIN M (APOM_HUMAN)

The two-dimensional electrophoresis pattern of apoM represents a chain of spots in the pI-range 4.5–6.5 and Mw ~ 22,000 (Supplementary Figure S1). There are 13 N-linked annotations at 1 site (N135) in apoM (https://glygen.org/protein/O95445#glycosylation accessed on 10 September 2022).

### 2.27. Complement System

The results of several studies suggest that changes in the complement system can not only promote an antitumor response but can also influence tumor development through proliferation, survival, angiogenesis, and invasiveness [267,268]. The presence of many complement components with different functions makes the study of this system very difficult [269]. In any case, it is becoming clear that complement activation stimulates carcinogenesis and protects against immune destruction, although it has long been believed that the complement system helps the body identify and eliminate transformed cells. Moreover, the complement is activated by different mechanisms in the case of different types of cancer, and the results of activation may be different for different types of cancer or over time for the same tumor [270,271,272].

#### 2.27.1. C1R (C1R_HUMAN)

The two-dimensional electrophoresis pattern represents only a chain of spots of the complement C1r subcomponent in the pI-range 4.5–6.2 and Mw ~ 80,000 (Supplementary Figure S1) that corresponds to only a complement C1r subcomponent. The cleaved heavy and light chains were not detected. There are 25 N-linked glycosylation annotations at four sites and one phosphorylation site in the complement C1r subcomponent (https://glygen.org/protein/P00736#glycosylation accessed on 10 September 2022).

#### 2.27.2. C1S (C1S_HUMAN)

The two-dimensional electrophoresis-pattern represents a chain of five spots of C1s in the pI-range from 4.0 to 4.9 and Mw ~ 80,000 (Supplementary Figure S1). The cleaved heavy and light chains were not detected. There are seven N-linked glycans at two sites (https://glygen.org/protein/P09871#glycosylation accessed on 10 September 2022).

#### 2.27.3. COMPLEMENT C1qC (C1QC_HUMAN)

The two-dimensional electrophoresis pattern of C1q represents a long horizontal chain of spots in the pI-range 3.0–9.5 with Mw ~ 23,000 and a vertical chain of heavy complexes (Mw 23,000 and up) with pI ~ 9.0 (Supplementary Figure S1). It was reported there was only one O-linked glycosylation of C1q (https://glygen.org/protein/P02747#glycosylation accessed on 10 September 2022).

#### 2.27.4. COMPLEMENT FACTOR I (CFAI_HUMAN)

The two-dimensional electrophoresis pattern of the complement factor I represents the chains of many spots in the pI-range 4.5–6.8 from Mw ~ 64,000 (complement factor I) to Mw ~ 30,000 (the complement factor I heavy and light chains) (Supplementary Figure S1). In the SWISS-2DPAGE, the complement factor I is represented only by one spot (pI/Mw: 5.03/37,900). There are 57 N-linked glycosylation annotations at 6 sites for the complement factor I (https://glygen.org/protein/P05156#glycosylation accessed on 10 September 2022).

#### 2.27.5. COMPLEMENT FACTOR B (CFAB_HUMAN)

The two-dimensional electrophoresis pattern of the complement factor B represents the chains of spots in the pI-range 4.5–6.8 with Mw ~ 90,000 (Supplementary Figure S1). The cleaved heavy and light chains were not detected. In the SWISS-2DPAGE, the complement factor B is represented by a chain of six spots (pI 5.88–6.28, Mw ~ 100,000). There are 19 N-linked glycans (4 sites), and 3 O-linked glycans (3 sites) in the complement factor B (https://glygen.org/protein/P00751#glycosylation accessed on 10 September 2022).

#### 2.27.6. COMPLEMENT FACTOR D (CFAD_HUMAN)

The two-dimensional electrophoresis pattern of the complement factor D represents two spots (pI ~8.0, Mw 25,000) (Supplementary Figure S1). The protein can be phosphorylated (2 sites), glycosylated (2 sites), ubiquitinated (2 sites), and methylated (1 site) (https://www.phosphosite.org accessed on 10 September 2022).

#### 2.27.7. COMPLEMENT FACTOR H (CFAH_HUMAN)

The two-dimensional electrophoresis pattern of the complement factor D represents a long chain of spots in the pI-range 5.5–7 with Mw ~ 140,000 (Supplementary Figure S1). It was reported there were 62 N-linked glycans in 9 sites in the complement factor D (https://glygen.org/protein/P08603#glycosylation accessed on 10 September 2022).

#### 2.27.8. COMPLEMENT C2 (CO2_HUMAN)

The two-dimensional electrophoresis pattern of the complement C2 represents a chain of spots in the pI-range 6–7 with Mw ~ 80,000 (Supplementary Figure S1). It was reported there were 33 N-linked glycosylations at 9 sites and one phosphorylation (S266) of the complement C2 (https://glygen.org/protein/P06681#glycosylation accessed on 10 September 2022).

#### 2.27.9. COMPLEMENT C3 (CO3_HUMAN)

The two-dimensional electrophoresis pattern of the complement C3 represents a cluster of spots in the pI-range 3.5–7.5 and Mw from ~30,000 to 180,000 (Supplementary Figure S1). In the SWISS-2DPAGE, there are the complement C3 beta chain (5 spots with pI 6.81–6.98, Mw ~ 71,000) and the complement C3dg fragment (a spot with pI 4.84 and Mw 40,915). There are 50 N-linked glycans at 4 sites, 2 O-linked glycans at 2 sites, and 12 phosphorylation sites (https://glygen.org/protein/P01024#glycosylation accessed on 10 September 2022).

#### 2.27.10. COMPLEMENT C4-A (CO4A_HUMAN)

The two-dimensional electrophoresis pattern of the complement C4-A represents a wide cluster of spots in the pI-range 3.0–10.0 and Mw from ~25,000 to 190,000 (Supplementary Figure S1). There are 35 N-linked glycans (4 sites), 6 O-linked glycans (4 sites), and 3 phosphoserine sites (https://glygen.org/protein/P0C0L4#glycosylation accessed on 10 September 2022).

#### 2.27.11. COMPLEMENT C4-B (C4B) (CO4B_HUMAN)

The two-dimensional electrophoresis pattern of the complement C4-B represents a wide cluster of spots in the pI-range 3.0–10.0 and Mw from ~35,000 to 19,0000 (Supplementary Figure S1). In the SWISS-2DPAGE, only a complement C4 gamma chain (2 spots with pI/Mw: 6.41/31,942 and 6.54/31,735) was detected. It was reported there were 34 N-linked glycans (4 sites) and 1 O-linked glycan (1 site) in the complement C4-B (https://glygen.org/protein/P0C0L5#glycosylation accessed on 10 September 2022).

#### 2.27.12. COMPLEMENT C5 (CO5_HUMAN)

The two-dimensional electrophoresis pattern of the complement C4-B represents a wide cluster of spots in the pI-range 5.0–6.8 and Mw ~ 70,000–190,000 (Supplementary Figure S1). It was reported there were eight N-linked glycans at three sites (https://glygen.org/protein/P01031#glycosylation accessed on 10 September 2022).

#### 2.27.13. COMPLEMENT C6 (CO6_HUMAN)

The two-dimensional electrophoresis pattern of the complement C6 represents a chain of spots in the pI-range 4.0–6.5 and Mw ~ 100,000 (Supplementary Figure S1). It was reported there were 6 C-linked annotations at 6 sites, 12 N-linked annotations at 4 sites, 2 O-linked annotations at 2 sites (https://glygen.org/protein/P13671#glycosylation accessed on 10 September 2022), and 5 sites of phosphorylation (https://www.phosphosite.org/ accessed on 10 September 2022).

#### 2.27.14. COMPLEMENT C7 (CO7_HUMAN)

The two-dimensional electrophoresis pattern of the complement C7 represents a chain of spots in the pI-range 4.5–6.5 and Mw ~ 100,000 (Supplementary Figure S1). It was reported there were three N-linked glycans at two sites and two O-linked glycans at one site (https://glygen.org/protein/P10643#glycosylation accessed on 10 September 2022).

#### 2.27.15. COMPLEMENT C9 (CO9_HUMAN)

The two-dimensional electrophoresis pattern of the complement C9 represents a chain of spots in the pI-range 4.5–5.5 and Mw ~ 60,000 (Supplementary Figure S1). The protein can be glycosylated (10 N-linked glycans at 2 sites, 4 O-linked glycans at 5 sites), phosphorylated (10 sites), acetylated (1 site), and ubiquitinated (3 sites) (https://www.phosphosite.org/ accessed on 10 September 2022).

### 2.28. CARBONIC ANHYDRASE I (CAH1_HUMAN)

The two-dimensional electrophoresis pattern of CAB represents a chain of spots in the pI-range 5–7 and Mw ~ 28,000 (Supplementary Figure S1). It was reported there was glycosylation (2 O-linked at 2 sites) (https://glygen.org/protein/P00915#glycosylation accessed on 10 September 2022), phosphorylation (11 sites), acetylation (5 sites), and ubiquitylation (2 sites) (https://www.phosphosite.org/ accessed on 10 September 2022).

### 2.29. CORTICOSTEROID-BINDING GLOBULIN (CBG_HUMAN)

The two-dimensional electrophoresis pattern of CBG represents a chain of spots in the pI-range 3.7–5.1 and Mw ~ 50,000 (Supplementary Figure S1). It was reported there were 48 N-linked glycosylations at 6 sites and 2 O-linked glycosylations at 1 site (https://glygen.org/protein/P08185#glycosylation accessed on 10 September 2022).

### 2.30. CARBOXYPEPTIDASE N CATALYTIC CHAIN (CBPN_HUMAN)

The two-dimensional electrophoresis pattern of CBPN represents a chain of spots in the pI-range 4.5–7 and Mw ~ 50,000 (Supplementary Figure S1). It was reported there were 32 N-linked glycans at 5 sites and 2 O-linked glycans at 1 site https://glygen.org/protein/P08185#glycosylation accessed on 10 September 2022.

### 2.31. MONOCYTE DIFFERENTIATION ANTIGEN CD14 (CD14_HUMAN)

The two-dimensional electrophoresis pattern of CD14 represents a chain of spots in the pI-range 4.5–5.8 and Mw ~ 40,000 (Supplementary Figure S1). It was reported there were 26 N-linked glycans at 2 sites and 4 O-linked glycans at 3 sites (https://glygen.org/protein/P08571#glycosylation accessed on 10 September 2022).

### 2.32. CERULOPLASMIN (CERU_HUMAN)

The two-dimensional electrophoresis pattern of ceruloplasmin represents a chain of spots in the pI-range 4.0–6.2 and Mw ~ 120,000 (Supplementary Figure S1). In the SWISS-2DPAGE, 3 chains of 27 spots with pI 4.96–5.24 and Mw ~ 120–16,0000 are present. There are 237 N-linked annotations at 8 sites, 10 O-linked annotations at 7 sites of glycosylation, and 3 sites of phosphorylation for ceruloplasmin (https://glygen.org/protein/P00450#glycosylation accessed on 10 September 2022).

### 2.33. CHOLINESTERASE (CHLE_HUMAN)

The two-dimensional electrophoresis pattern of cholinesterase represents a chain of five spots in the pI-range 4.5–5.2 and Mw ~ 65,000 (Supplementary Figure S1). There are 34 N-linked annotations at 12 sites, one O-linked annotation for glycosylation, and phosphorylation at S226 for cholinesterase (https://glygen.org/protein/P06276#glycosylation accessed on 10 September 2022).

### 2.34. CLUSTERIN (CLUS_HUMAN)

The two-dimensional electrophoresis pattern of ceruloplasmin represents a chain of 18 spots in the pI-range 4.5–6.5 and Mw ~ 35,000 (Supplementary Figure S1). In the SWISS-2DPAGE, 17 spots with pI 4.73–5.07 and Mw ~ 35–39,000 are shown. Clusterin is heavily glycosylated (149 N-linked glycans at 6 sites, O-linked glycan at 1 site) and phosphorylated (4 sites) (https://glygen.org/protein/P10909#glycosylation accessed on 10 September 2022).

### 2.35. BETA-ALA-HIS DIPEPTIDASE (CNDP1_HUMAN)

The two-dimensional electrophoresis pattern of beta-Ala-His dipeptidase represents two spots around pI 5.0 and Mw ~ 54,000 (Supplementary Figure S1). It was reported there were 18 N-linked glycans at 1 site, 1 O-linked glycan at 2 sites, and phosphorylation at S219 (https://glygen.org/protein/Q96KN2#glycosylation accessed on 10 September 2022).

### 2.36. CARBOXYPEPTIDASE N SUBUNIT 2 (CPN2_HUMAN)

The two-dimensional electrophoresis pattern of carboxypeptidase N subunit 2 represents nine spots with pI 3.5–5.5 and Mw ~ 65,000 (Supplementary Figure S1). It is known there were 10 N-linked glycans at 3 sites in carboxypeptidase N subunit 2 (https://www.glygen.org/protein/P22792 accessed on 10 September 2022).

### 2.37. C-REACTIVE PROTEIN (CRP_HUMAN)

The two-dimensional electrophoresis pattern of CRP represents a single spot (pI/Mw: 5.2/24,000) (Supplementary Figure S1). In the SWISS-2DPAGE, a similar situation exists—a single spot (pI/Mw: 5.12/23,760). Thus far, it was reported there was only one PTM (a pyroglutamic acid, Q19) for CRP (https://www.uniprot.org/uniprotkb/P02741/entry accessed on 10 September 2022).

### 2.38. EXTRACELLULAR MATRIX PROTEIN I (ECM1_HUMAN)

The two-dimensional electrophoresis pattern of ECM1 represents a spot (pI/Mw: 6.0/60,000) (Supplementary Figure S1). It was reported there were 21 N-linked glycans at 4 sites, and 3 O-linked glycans at 6 sites (https://www.glygen.org/protein/Q16610 accessed on 10 September 2022).

### 2.39. FIBULIN-1 (FBLN1_HUMAN)

The two-dimensional electrophoresis pattern of FIBL-1 represents a chain of four spots (pI/Mw: 4.5–5.2/75,000) (Supplementary Figure S1). It was reported there were 10 N-linked glycans at two sites, one O-linked glycan, and one phosphorylation at S147 (https://www.glygen.org/protein/P23142 accessed on 10 September 2022).

### 2.40. FICOLIN-3 (FCN3_HUMAN)

The two-dimensional electrophoresis-pattern of ficolin-3 represents a chain of five spots (pI/Mw: 5.8–6.5/30,000) (Supplementary Figure S1). It was reported there were five N-linked annotation(s) at one site (https://www.glygen.org/protein/O75636 accessed on 10 September 2022).

### 2.41. ALPHA-2-HS-GLYCOPROTEIN (FETUA_HUMAN)

The two-dimensional electrophoresis-pattern of fetuin-A represents a set of proteoforms (pI/Mw: 3.7–6.3/~40,000-up) (Supplementary Figure S1). In the SWISS-2DPAGE, 15 spots (pI/Mw: 4.56–4.77/52–58,000) are shown. The protein is heavily glycosylated (126 N-linked annotations at 2 sites, 43 O-linked annotations at 14 sites) and phosphorylated (https://www.glygen.org/protein/P02765 accessed on 10 September 2022).

### 2.42. FETUIN-B (FETUB_HUMAN)

The two-dimensional electrophoresis pattern of fetuin-B represents a chain of proteoforms (pI/Mw: 5.0–6.3/50,000-up) (Supplementary Figure S1). The protein is heavily glycosylated (26 N-linked annotations at 3 sites, 8 O-linked annotation(s) at 6 sites) and phosphorylated (https://www.glygen.org/protein/Q9UGM5 accessed on 10 September 2022).

### 2.43. FIBRINOGEN ALPHA CHAIN (FIBA_HUMAN)

The two-dimensional electrophoresis pattern of FBA represents several sets of chains with pI 5.0–7.5 (Mw ~ 30–35,000, Mw ~ 64–83,000, Mw ~ 110,000 and up) (Supplementary Figure S1). In the SWISS-2DPAGE, a double chain of 19 spots (pI/Mw: 6.65–7.78/63–67,000) is presented [22]. The protein can be heavily glycosylated (12 N-linked annotations at 3 sites, 43 O-linked annotations at 34 sites) and phosphorylated (https://www.glygen.org/protein/P02671 accessed on 10 September 2022).

### 2.44. FIBRINOGEN BETA CHAIN (FIBB_HUMAN)

The two-dimensional electrophoresis-pattern of FBB represents a chain of spots (pI/Mw: 5.5–8.5/~52,000) (Supplementary Figure S1). In the SWISS-2DPAGE, a chain of four spots (pI/Mw: 6.1–6.55/55–56,000) is presented [22]. The protein can be glycosylated (52 N-linked annotations at 4 sites and 5 O-linked annotations at 3 sites) (https://www.glygen.org/protein/P02675 accessed on 10 September 2022).

### 2.45. FIBRINOGEN GAMMA CHAIN (FIBG_HUMAN)

The two-dimensional electrophoresis-pattern of FGG represents a chain of spots with pI 4.5–7 (Mw ~ 50,000) (Supplementary Figure S1). In the SWISS-2DPAGE, 3 chains of 13 spots (pI/Mw: 5.07–5.65/44–51,000) are presented [22]. The protein is glycosylated (39 N-linked annotations at 4 sites, 1 O-linked annotation at 1 site) and phosphorylated at S68 (https://www.glygen.org/protein/P02679 accessed on 10 September 2022).

### 2.46. FIBRONECTIN (FINC_HUMAN)

The two-dimensional electrophoresis pattern of fibronectin represents a chain of spots with pI 4.5–6.7 (Mw ~ 112,000 up) (Supplementary Figure S1). The protein is heavily glycosylated (265 N-linked annotations at 13 sites, 37 O-linked annotations at 25 sites) and phosphorylated (https://www.glygen.org/protein/P02751 accessed on 10 September 2022).

### 2.47. PLASMA GELSOLIN (GELS_HUMAN)

The two-dimensional electrophoresis pattern of gelsolin represents a chain of spots with pI 4.5–6.5 (Mw ~ 83,000) (Supplementary Figure S1). The protein can be heavily phosphorylated (25 sites), acetylated (12 sites), and ubiquitinated (10 sites) (https://www.phosphosite.org accessed on 10 September 2022).

### 2.48. GLUTATHION PEROXIDASE 3 (GPX3_HUMAN)

The two-dimensional electrophoresis pattern of this protein represents a chain of spots with pI 4.9–6.9 (Mw ~ 25,000) (Supplementary Figure S1). It is phosphorylated (4 sites) and acetylated (3 sites) (https://www.phosphosite.org/ accessed on 10 September 2022).

### 2.49. HEMOGLOBIN SUBUNIT ALPHA (HBA_HUMAN)

The two-dimensional electrophoresis pattern of this protein represents a chain of spots with pI 7.5–9 (Mw ~ 15,000) (Supplementary Figure S1). In the SWISS-2DPAGE, two spots (pI/Mw: 9.2/11,000, 8.9/11,000) are presented. The protein is glycosylated (3 O-linked annotations at 3 sites), glycated (6 sites) (https://www.glygen.org/protein/P69905 accessed on 10 September 2022), phosphorylated (17 sites), acetylated (4 sites), and ubiquitinated (8 sites) (https://www.phosphosite.org/ accessed on 10 September 2022).

### 2.50. HEMOGLOBIN SUBUNIT BETA (HBB_HUMAN)

The two-dimensional electrophoresis-pattern of this protein represents a chain of spots with pI 6.5–6.9 (Mw ~ 15,000) (Supplementary Figure S1). In the SWISS-2DPAGE, two spots (pI/Mw: 7/15,000, 6.9/15,000) are presented. The protein is glycosylated (4 O-linked annotations at 4 sites), glycated (6 sites), and phosphorylated (14 sites) (https://www.glygen.org/protein/P68871 accessed on 10 September 2022).

### 2.51. HEMOPEXIN (HEMO_HUMAN)

The two-dimensional electrophoresis pattern of this protein represents a chain of spots with pI 5–6.9 (Mw ~ 50,000) (Supplementary Figure S1). In the SWISS-2DPAGE, chains of five spots (pI 5.25–5.59/Mw ~ 72–77,000) and two spots (4.48/19,274, 4.56/18,289) are presented. The protein is glycosylated (184 N-linked annotations at 6 sites, 21 O-linked annotations at 6 sites (https://www.glygen.org/protein/P02790 accessed on 10 September 2022)).

### 2.52. HEPARIN COFACTOR 2 (HEP2_HUMAN)

The two-dimensional electrophoresis-pattern of this protein represents a chain of spots with pI 4.9–6.5 (Mw ~ 55,000) (Supplementary Figure S1). The protein is glycosylated (39 N-linked annotations at 3 sites, 13 O-linked annotations at 9 sites) and phosphorylated at S37 (https://www.glygen.org/protein/P05546 accessed on 10 September 2022).

### 2.53. HAPTOGLOBIN (HPT_HUMAN)

**Figure 2:**
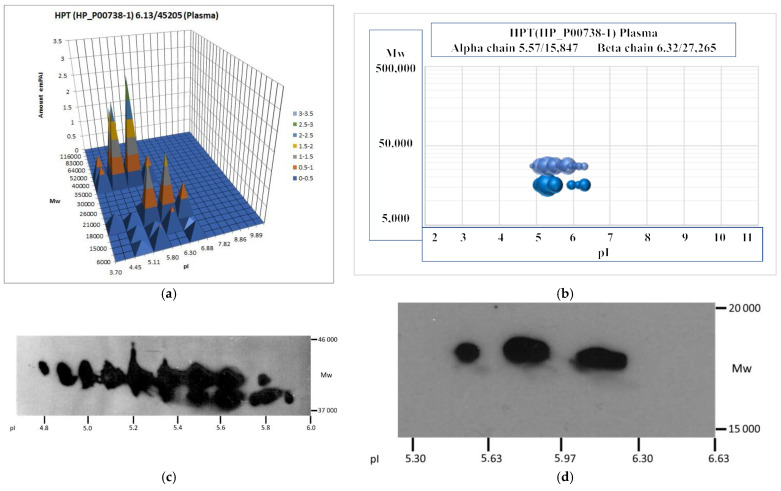
Two-dimensional electrophoresis patterns of haptoglobin alpha and beta chains. (**a**) A sectional analysis of alpha and beta chains. The stained gel was divided into the sections with the predetermined coordinates. Then each section was treated and analyzed by LC ESI-MS/MS (see Materials and Methods, Section 4.2, 2DE). According to the abundance (emPAI) of Hpt in each section, the graph was plotted. (**b**) A semi-virtual 2DE of alpha and beta chains (see Materials and Methods, Section 4.2, 2DE). According to the emPAI of alpha chain (theoretical Mw: 15,946) or beta chain (theoretical Mw: 27,265) in the sections, the graph was plotted. The ball size is proportional to emPAI in each section. (**c**) Two-dimensional electrophoresis–Western of beta chain. (**d**) Two-dimensional electrophoresis–Western of alpha2 chain.

The two-dimensional electrophoresis pattern of Hp represents ~16 spots of beta chains with pI 4.8–6.0 (Mw ~ 40,000) and 3 spots of alpha 2 chain (Figure 2). In the SWISS-2DPAGE, a chain of 19 spots (pI 4.88–5.86/Mw ~ 40,000, beta chain), 3 spots (pI 5.68–6.37/Mw ~ 17,000, alpha 2 chain), and 2 spots (pI 5.13–5.37/Mw ~ 12,000, alpha 1 chain) are presented. Hp is heavily glycosylated (351 N-linked annotations at 4 sites, 1 O-linked annotations at 1 site) (https://www.glygen.org/protein/P00738 accessed on 10 September 2022).

### 2.54. HAPTOGLOBIN-RELATED PROTEIN (HPTR_HUMAN)

The two-dimensional electrophoresis pattern of this protein represents a chain of spots with pI 4.6–6.5 (Mw ~ 40,000) (Supplementary Figure S1). The protein is N-linked glycosylated (5 sites), acetylated (1 site), and ubiquitinated (2 sites) (https://www.phosphosite.org/ accessed on 10 September 2022).

### 2.55. HISTIDINE-RICH GLYCOPROTEIN (HRG_HUMAN)

The two-dimensional electrophoresis-pattern of this protein represents a chain of spots with pI 4.5–7.8 (Mw ~ 64,000) and spots around pI/Mw: 5.5/53,000 (Supplementary Figure S1). In the SWISS-2DPAGE, only a single spot (pI/Mw: 5.3/53,000) is present. The protein is glycosylated (44 N-linked glycans at 4 sites, 4 O-linked glycans at 3 sites) (https://www.glygen.org/protein/P04196 accessed on 10 September 2022).

### 2.56. PLASMA PROTEASE C1 INHIBITOR (IC1_HUMAN)

The two-dimensional electrophoresis pattern of this protein represents a long chain of spots with pI 3.2–5.2 (Mw ~ 64,000). The protein is heavily glycosylated (107 N-linked annotations at 8 sites, 33 O-linked annotations at 21 sites) (https://www.glygen.org/protein/P05155 accessed on 10 September 2022).

### 2.57. INTER-ALPHA-TRYPSIN INHIBITOR HEAVY CHAINS (ITIH1, ITIH2, ITIH3, ITIH4, ITIH5)

In our experiments, 2DE patterns of these proteins are presented by the chains of the precursor proteoforms and the mature ITIH1 (Supplementary Figure S1). The proteins are heavily glycosylated, phosphorylated, acetylated, and ubiquitinated (https://www.phosphosite.org/ accessed on 10 September 2022).

### 2.58. *KALLISTATIN (KAIN_HUMAN)*

In our experiments, the 2DE pattern of this protein is presented as a cluster of proteoforms around pI/Mw: 6–7/40–120,000 (Supplementary Figure S1). The protein is glycosylated (31 N-linked annotations at 4 sites) and phosphorylated (https://www.glygen.org/protein/P29622 accessed on 10 September 2022, https://www.phosphosite.org/ accessed on 10 September 2022.

### 2.59. KININOGEN 1 (KNG1_HUMAN)

The two-dimensional electrophoresis pattern of this protein represents multiple spots (pI 3.5–8.5, Mw ~ 35–64,000) (Supplementary Figure S1). In the SWISS-2DPAGE, only a single spot (pI/Mw: 6.48/7490) is present. The protein can be heavily glycosylated (159 N-linked annotations at 6 sites, 65 O-linked annotations at 26 sites) (https://www.glygen.org/protein/P01042 accessed on 10 September 2022), phosphorylated, acetylated, and ubiquitinated (https://www.phosphosite.org/ accessed on 10 September 2022).

### 2.60. PHOSPHATIDYLCHOLINE-STEROL ACYLTRANSFERASE (LCAT_HUMAN)

The two-dimensional electrophoresis pattern of this protein represents three spots (pI 4.0–4.8, Mw ~ 50,000) (Supplementary Figure S1). The protein is glycosylated (22 N-linked annotations at 5 sites, 3 O-linked annotations at 3 sites) (https://www.glygen.org/protein/P04180 accessed on 10 September 2022), phosphorylated, and ubiquitinated (https://www.phosphosite.org/ accessed on 10 September 2022).

### 2.61. LUMICAN (LUM_HUMAN)

The two-dimensional electrophoresis pattern of this protein represents chains of spots (pI 4.5–6.5, Mw ~ 52–83,000) (Supplementary Figure S1). The protein has multiple PTMs: (97 N-linked glycans at 4 sites, 2 O-linked glycans at 3 sites, phosphorylation (11 sites), and acetylation (8 sites) (https://www.uniprot.org/uniprotkb/P51884/entry accessed on 10 September 2022).

### 2.62. MANNOSE-BINDING PROTEIN C (MBL2_HUMAN)

The two-dimensional electrophoresis pattern of this protein represents just a single spot (pI/Mw: 5.3/26,000) (Supplementary Figure S1).

### 2.63. PIGMENT EPITHELIUM-DERIVED FACTOR (PEDF_HUMAN)

The two-dimensional electrophoresis-pattern of this protein represents a chain of spots (pI 4.5–6.5, Mw ~ 40–52,000) (Supplementary Figure S1). The protein is glycosylated (10 N-linked glycans at 1 site, 2 O-linked glycans at 5 sites), phosphorylated (10 sites), acetylated, and methylated (https://www.uniprot.org/uniprotkb/P36955/entry accessed on 10 September 2022).

### 2.64. N-ACETYLMURAMOYL-L-ALANINE AMIDASE (PGRP2_HUMAN)

The two-dimensional electrophoresis pattern of this protein represents a chain of spots (pI 5.5–6.8, Mw ~ 52–64,000) (Supplementary Figure S1). The protein is glycosylated (12 N-linked glycans at 3 sites, 4 O-linked glycans at 7 sites) and phosphorylated (4 sites) (https://www.uniprot.org/uniprotkb/Q96PD5/entry accessed on 10 September 2022).

### 2.65. PHOSPHATIDYLINOSITOL-GLYCAN-SPECIFIC PHOSPHOLIPASE D (PHLD_HUMAN)

The two-dimensional electrophoresis pattern of this protein represents a chain of spots (pI 4.2–5.6, Mw ~ 83–116,000) (Supplementary Figure S1). The protein is glycosylated (10 sites, 22 N-linked glycans at 4 sites), phosphorylated (5 sites), and acetylated (https://www.phosphosite.org/ accessed on 10 September 2022).

### 2.66. PLASMINOGEN (PLMN_HUMAN)

The two-dimensional electrophoresis pattern of this protein represents two chains of multiple spots (pI 3.3–4.1, Mw ~ 83–116,000) and (pI 6.7–8.5, Mw ~ 83–116,000) (Supplementary Figure S1). In the SWISS-2DPAGE, a single chain (7 spots) is present (pI 6.32–6.49, Mw ~ 112–116,000). The protein is glycosylated (54 N-linked glycans at 4 sites, 12 O-linked glycans at 12 sites) and phosphorylated (15 sites) (https://www.uniprot.org/uniprotkb/P00747/entry accessed on 10 September 2022).

### 2.67. PARAOXONASE (PON1_HUMAN)

The two-dimensional electrophoresis pattern of this protein represents a cluster of spots (pI 4.0–5.5, Mw ~ 35–52,000) (Supplementary Figure S1). In the SWISS-2DPAGE, two spots are presented (pI/Mw: 4.84/45,937 and 4.93/43,391). The protein is glycosylated (30 N-linked glycans at 3 sites), phosphorylated (3 sites), and acetylated (1 site) (https://www.uniprot.org/uniprotkb/P27169/entry accessed on 10 September 2022).

### 2.68. PROPERDIN (PROP_HUMAN)

The two-dimensional electrophoresis pattern of this protein represents two spots (pI/Mw: 8.5/52,000 and 8.7/52,000) (Supplementary Figure S1). The protein is glycosylated (15 C-linked annotations at 15 sites, 2 N-linked annotations at 1 site, 4 O-linked annotations at 4 sites).

### 2.69. VITAMIN K-DEPENDENT PROTEIN S (PROS_HUMAN)

The two-dimensional electrophoresis pattern of this protein represents a chain of spots (pI 3.5–4.5, Mw ~ 64–83,000) (Supplementary Figure S1). The protein is glycosylated (5 N-linked annotations at 3 sites, 4 O-linked annotations at 4 sites) and phosphorylated (8 sites) (https://www.uniprot.org/uniprotkb/P07225/entry accessed on 10 September 2022).

### 2.70. PLASMA RETINOL-BINDING PROTEIN (RET4_HUMAN)

The two-dimensional electrophoresis pattern of this protein represents a cluster of spots (pI 5.0–6.0, Mw ~ 18–26,000) (Supplementary Figure S1). In the SWISS-2DPAGE, three spots are presented (pI ~ 5.0, Mw ~ 20,000). The protein can be phosphorylated and methylated (https://www.phosphosite.org/ accessed on 10 September 2022).

### 2.71. SERUM AMYLOID A (SAA1_HUMAN)

The two-dimensional electrophoresis pattern of this protein represents two spots (pI/Mw: ~5.6/12,000 and ~5.8/12,000) (Supplementary Figure S1). This protein is phosphorylated (5 sites) (https://www.phosphosite.org/ accessed on 10 September 2022).

### 2.72. SERUM AMYLOID P (SAMP_HUMAN)

The two-dimensional electrophoresis pattern of this protein represents a cluster of spots (pI/Mw: ~4.5–6.1/21–35,000) (Supplementary Figure S1). This protein is phosphorylated (7 sites), acetylated (3 sites), ubiquitinated (2 sites) (https://www.phosphosite.org/ accessed on 10 September 2022), and glycosylated (14 N-linked glycans at 1 site, 1 O-linked glycan) (https://www.glygen.org/protein/P02743 accessed on 10 September 2022).

### 2.73. SEX HORMONE-BINDING GLOBULIN (SHBG_HUMAN)

The two-dimensional electrophoresis pattern of this protein represents a chain of spots (pI/Mw: ~5.0–6.0/35–52,000) (Supplementary Figure S1). This protein is phosphorylated (4 sites) (https://www.phosphosite.org/ accessed on 10 September 2022) and glycosylated (12 N-linked glycans at 3 sites, 6 O-linked glycans at 1 site).

### 2.74. S100A8 (CALPOTECTIN) (S10A8_HUMAN)

The two-dimensional electrophoresis pattern of this protein represents one spot (pI/Mw: ~6.8/10,000) (Supplementary Figure S1). However, this protein can be heavily phosphorylated (9 sites), acetylated (https://www.phosphosite.org/ accessed on 10 September 2022), and glycosylated (1 O-linked glycan) (https://www.uniprot.org/ accessed on 10 September 2022).

### 2.75. S100A9 (CALPOTECTIN) (S10A9_HUMAN)

The two-dimensional electrophoresis pattern of S100-A9 represents one spot (pI/Mw: ~6.0/10,000) (Supplementary Figure S1). This protein can be phosphorylated (5 sites), acetylated (3 sites), methylated (https://www.phosphosite.org/ accessed on 10 September 2022), and glycosylated (1 O-linked glycan) (https://www.uniprot.org/ accessed on 10 September 2022).

### 2.76. TETRANECTIN (TETN_HUMAN)

The two-dimensional electrophoresis pattern of this protein represents a chain of spots (pI/Mw: ~5–6.5/21–26,000) (Supplementary Figure S1). This protein can be glycosylated (1 O-linked annotation(s) at 1 site) (https://www.uniprot.org/uniprotkb/P05452/entry accessed on 10 September 2022).

### 2.77. THYROXINE-BINDING GLOBULIN (THBG_HUMAN)

The two-dimensional electrophoresis pattern of this protein represents a chain of spots (pI/Mw: ~5–5.5/52–64,000) (Supplementary Figure S1). This protein can be glycosylated (17 N-linked glycans at 3 sites, 1 O-linked glycan) and phosphorylated (https://www.phosphosite.org/ accessed on 10 September 2022).

### 2.78. PROTHROMBIN (THRB_HUMAN)

The two-dimensional electrophoresis pattern of this protein represents a chain of spots (pI/Mw: 5–6.5/64–83,000) (Supplementary Figure S1). In the SWISS-2DPAGE, there is a chain of five spots (pI/Mw: 4.95–5.05/80,000). This protein can be glycosylated (2 N-linked glycans at 5 sites, 1 O-linked glycan at 6 sites), phosphorylated (9 sites), acetylated (1 site), and ubiquitinated (4 sites) (https://www.uniprot.org/uniprotkb/P00734/entry accessed on 10 September 2022).

### 2.79. SEROTRANSFERRIN (TRFE_HUMAN)

The two-dimensional electrophoresis pattern of this protein represents a cluster of spots (pI/Mw: ~5.7–7.0/64–83,000) (Supplementary Figure S1). In the SWISS-2DPAGE, there are 3 chains of 22 spots (pI/Mw: 6.14–6.64/76–87,000) for serotransferrin. This protein can be glycosylated (145 N-linked glycans at 6 sites, 6 O-linked glycans at 2 sites), phosphorylated (21 sites), acetylated (10 sites), and ubiquitinated (7 sites) (https://www.phosphosite.org/ accessed on 10 September 2022).

### 2.80. TRANSTHYRETIN (TTHY_HUMAN)

The two-dimensional electrophoresis pattern of this protein represents a chain of spots (pI/Mw: 4.8–5.7/15–18,000) (Supplementary Figure S1). In the SWISS-2DPAGE, there are a chain of three spots (pI/Mw: 5.02–5.52/13,800), and a spot (pI/Mw: 5.52/35,391) for transthyretin. This protein can be glycosylated (1 N-linked annotation), phosphorylated (6 sites), acetylated (2 sites), or ubiquitinated (4 sites) (https://www.phosphosite.org/ accessed on 10 September 2022).

### 2.81. VITAMIN D-BINDING PROTEIN (VTDB_HUMAN)

The two-dimensional electrophoresis pattern of this protein represents a cluster of spots (pI/Mw: 4.5–5.7/40–52,000) (Supplementary Figure S1). In the SWISS-2DPAGE, there are two spots (pI/Mw: 5.16/53,772 and 5.24/53,918) for vitamin D-binding protein. This protein can be glycosylated (1 N-Linked glycan at 1 site, 1 O-Linked glycan), phosphorylated (12 sites), acetylated (1 site), or ubiquitinated (1 site) (https://www.phosphosite.org/ accessed on 10 September 2022).

### 2.82. VITRONECTIN (VTNC_HUMAN)

The two-dimensional electrophoresis pattern of this protein represents a cluster of spots (pI/Mw: 3.7–6.6/52–116,000) (Supplementary Figure S1). In the SWISS-2DPAGE, there is only one spot (pI/Mw: 4.58/9248) for vitronectin. VN can be glycosylated (1 N-linked annotation), phosphorylated (20 sites), acetylated (1 site), and ubiquitinated (1 site) (https://www.phosphosite.org/ accessed on 10 September 2022). 

### 2.83. ZINC-ALPHA-2-GLYCOPROTEIN (ZA2G_HUMAN)

The two-dimensional electrophoresis pattern of this protein represents a cluster of spots (pI/Mw: 4.2–5.0/35–40,000) (Supplementary Figure S1). In the SWISS-2DPAGE, there is a chain of four spots (pI/Mw: 4.8–4.97/40–42,000) for zinc-alpha-2-glycoprotein. This protein can be glycosylated (108 N-Linked glycans at 3 sites, 1 O-Linked glycan at 1 site), phosphorylated (1 site), and ubiquitinated (5 sites) (https://www.phosphosite.org/ accessed on 10 September 2022).

## 3. Discussion

Tumorigenesis leads to multiple variations in the human plasma proteome that can be dynamic and alterable during the progress of the disease. Practically all major, so-called “classical”, plasma proteins change abundances or PTMs. The majority of these proteins are secreted by the liver, so it could be anticipated to see these changes only in the case of liver cancer. However, they can be observed with other tumors as well as cancer induces disturbances in the blood homeostasis that is supported by “classical plasma proteins”. It follows that it is possible to search the specific/unspecific ways of tumor prediction not only through the detection of products of the tumor but also by analyzing the changes in “classic plasma proteins”. It is relevant to mention that plasma analysis by a very different approach, differential scanning calorimetry (DSC), can give us a hint. Typically, DSC is used to determine the partial heat capacity of macromolecules as a function of temperature, from which their structural stability during thermal denaturation can be assessed. The method is very sensitive and allows precise determination of thermally-induced conformational transitions of proteins present in plasma. There are already quite a few publications showing that DSC can be used to distinguish between normal and cancerous plasma samples [273,274]. Moreover, the data obtained by this method can be reproduced using major plasma proteins.

It follows that there is a possibility of building test systems based on these major (“classical”) proteins. What is important is that many examples of such systems have been introduced already. For example, the relationship between inflammation and clinical outcome is described using the Modified Glasgow Prognostic Scale (mGPS), which includes levels of C-reactive protein (CRP) and albumin [275]. The combination of elevated CRP (>10 mg/L) and decreased albumin (<35 g/L) corresponds to higher mGPS, which correlates with systemic inflammation and poor outcome of cancer therapy [276]. The OVA1 test uses the other major plasma proteins. OVA1 is an FDA-approved blood test that measures the levels of five proteins (CA125, transferrin, transthyretin, apolipoprotein A1, and beta-2 microglobulin) to detect ovarian cancer risk in women. Here, a sophisticated mathematical formula (multivariate index assay) is used to evaluate and combine the levels of these proteins in plasma, producing an ovarian cancer risk score. Using this approach, OVA1 can detect early-stage ovarian cancer with 98% specificity. The OVERA (second-generation or OVA2) assesses a woman’s malignancy risk using combined results from the following five proteins: apolipoprotein A1, human epididymis protein 4 (HE4), CA-125 II, follicle-stimulating hormone (FSH), and transferrin (Vermillion Inc. OVA1 Products. Updated 2020. Available at: https://vermillion.com/ova-products accessed on 10 September 2022). The observation of enhanced levels of clusterin, ITIH4, antithrombin-III, and C1RL in sera of endometrial cancer patients allowed a mathematical model to be built to detect cancer samples [29]. Accordingly, by the selection of the appropriate panels (proteomics signatures) of the plasma oncomarkers, it is possible to detect/monitor different types of cancers. The main point is to select the correct set of oncomarkers and develop an algorithm that will take into account all possible changes in these oncomarkers (level, PTMs etc.) that are related to cancer. This selection should be meticulously performed based on oncomarker behavior in plasma, not in tissue. We performed a search for publications with information (level, PTMs) about “classical” plasma proteins in the case of malignant processes in the human body (Table 1). As levels of some oncomarkers behave differently in different cancers (rise or fall), the test could specifically detect the type of cancer. Apolipoproteins are a good example here. SAA1 and CRP are APPs that are routinely measured in the clinic. The level of apoA-1 is reduced in many cancers but increased in some [80]. The decreased level of apoA-I in plasma is observed in the case of de novo myelodysplastic syndromes [83], NSCLC [84], nasopharyngeal carcinoma (NPC) [85], esophageal squamous cell carcinoma [86], and BC [75], but it is increased in SCLC, HCC, and bladder cancer [80]. The level of apoA-II is dramatically reduced in the serum of patients with gastric cancer and multiple myeloma [70,87] but increased in HCC and prostate cancer [88,89]. A similar situation can be observed for other apolipoproteins [80].

Another aspect that should be considered is the appearance of proteoforms produced by genetic polymorphisms, alternative splicing, PTMs, etc. These events change the charge (pI) and the weight (Mw) of the protein. Because of that, the experimental pI/Mw of the proteins can be different from the theoretical ones. This leads to the production of sets of proteoforms that in our case are detected as 2DE patterns. There is a belief that some 2DE patterns can be different between norm and cancer and could be used as specific biomarkers. Thus far, there are not many such examples, but progress in proteomics methods should improve the situation [277,278]. Proteomics is generating and analyzing a large volume of data and these data exactly fit the situation with multiple variations in plasma proteomes during cancer development and progression. Here, high-throughput, quantitative mass spectrometry is the best choice. There is already a good example of the possibility of using it in the clinic [14]. Geyer et al. introduced a rapid and robust “plasma proteome profiling” LC-MS/MS pipeline. Their single-run shotgun proteomics workflow enables quantitative analysis of hundreds of plasma proteins from just 1 µL of plasma [14].

Our aim is to build a comprehensive proteoform database containing norm and cancer samples http://2de-pattern.pnpi.nrcki.ru/ accessed on 10 September 2022 [30]. Glioblastoma and hepatocellular carcinoma are the cancers in our study so far. The database contains only the cellular samples, but we are in the process of incorporating tissue and plasma samples. 

## 4. Materials and Methods

### 4.1. Plasma

The pooled human plasma was from healthy male donors (age 20–47 years) [278,279]. Depletion of serum albumin and immunoglobulins IgG was carried out according to Agilent Multiple Affinity Removal System (MARS) protocol (“Agilent Technologies”, Santa Clara, CA, USA) [280,281].

### 4.2. Two-Dimensional Electrophoresis

The detailed process was described previously [280]. In short, 10 μL of plasma (0.5 mg of protein) was mixed with 20 μL of lysis buffer (7 M urea, 2 M thiourea, 4% CHAPS, 1% DTT, 2% (*v*/*v*) ampholytes, pH 3–10, protease inhibitor cocktail) and then with 100 μL of rehydrating buffer (7 M urea, 2 M thiourea, 2% CHAPS, 0.3% DTT, 0.5% IPG (*v*/*v*) buffer, pH 3–11 NL, 0.001% bromophenol blue). Immobiline DryStrip 3–11 NL (7 cm) was passively rehydrated by this solution for 4 h at 4 °C. IEF was run on Hoefer™ IEF100 (“Thermo Fisher Scientific”, Waltham, MA, USA). After IEF, strips were incubated 10 min in the equilibration solution (50 mM Tris, pH 8.8, 6 M urea, 2% SDS, 30% (*v*/*v*) glycerol, 1% DTT), following in the same solution with 5% IAM instead of DTT. The strips were sealed with a hot solution of 0.5% agarose prepared in electrode buffer (25 mM Tris, pH 8.3, 200 mM glycine, and 0.1% SDS) on top of the polyacrylamide gel (14%), and run in the second direction [280]. Gels stained by Coomassie Blue R350 were scanned by ImageScanner III and analyzed using Image Master 2D Platinum 7.0. For the sectional 2DE analysis, this gel was cut into 96 sections with determined coordinates. Each section (~0.7 cm^2^) was shredded and treated with trypsin. Tryptic peptides were eluted from the gel by extraction solution (5% (*v*/*v*) ACN, 5% (*v*/*v*) formic acid) and dried in Speed Vac. In the case of a semi-virtual 2DE, the 18-cm Immobiline DryStrip 3–11 NL was cut into 36 equal sections after IEF. For complete reduction, 300 μL of 3 mM DTT and 100 mM ammonium bicarbonate were added to each section and incubated at 50 °C for 15 min. For alkylation, 20 μL of 100 mM IAM were added and samples were incubated in the dark at r.t. for 15 min. The peptides were eluted with 60% acetonitrile and 0.1% TFA and dried in Speed Vac.

### 4.3. ESI LC-MS/MS Analysis

A detailed procedure was described previously [279,280]. Peptides were dissolved in 5% (*v*/*v*) formic acid. Tandem mass spectrometry analysis was conducted in duplicate on an Orbitrap Q-Exactive mass spectrometer (“Thermo Fisher Scientific”, Waltham, MA, USA). The data were analyzed by Mascot “2.4.1” (“Matrix Sciences”, Mount Prospect, IL, USA) or SearchGui [282] using the following parameters: enzyme—trypsin; maximum of missed cleavage sites—2; fixed modifications—carbamidomethylation of cysteine; variable modifications—oxidation of methionine, phosphorylation of serine, threonine, tryptophan, acetylation of lysine; the precursor mass error—10 ppm; the product mass error—0.01 Da. As a protein sequence database, UniProt (October 2014) was used.

Only 100% confident results of protein identification were selected. Two unique peptides per protein were required for all protein identifications. Exponentially modified PAI (emPAI), the exponential form of protein abundance index (PAI) defined as the number of identified peptides divided by the number of theoretically observable tryptic peptides for each protein, was used to estimate protein abundance [283].

### 4.4. Immunostaining (Western Blotting)

Plasma proteins (0.5 mg) were run by 2DE (cm 2DE, using 13-cm strip pH 4–7). Proteins were transferred (2 h, 28 V) from the gel onto PVDF membrane (Hybond P, 0.2 μm) using two sheets of thick paper (Bio-Rad, Hercules, CA, USA), saturated with 48 mM Tris, 39 mM glycine, 0.037% SDS, 20% ethanol. The membrane was treated following a protocol of Blue Dry Western [36] and treated with antibodies [21]. Primary antibodies were mouse monoclonal anti-Hp (C8, sc-376893, or F8, sc-390962, from “Santa Cruz Biotechnology”, Santa Cruz, CA, USA) in dilution 1/25 (80 ng/mL in TBS (25 mM), Tris (pH 7.5) and 150 mM NaCl containing 3% (*w*/*v*) BSA) or rabbit polyclonal anti-Hp (MBS177476, MyBioSource, San Diego, CA, USA). Secondary goat anti-mouse immunoglobulins G labeled by horseradish peroxidase (NA931V, “GE Healthcare”, Chicago, IL, USA ) were used in TBS containing 3% (*w*/*v*) nonfat dry milk (1/5000 dilution). The reaction was developed using ECL (Western Lightning Ultra, “PerkinElmer”, Waltham, MA, USA) and X-ray film (Amersham Hyper film ECL).

## 5. Conclusions

For now, proteomics is collecting big data about the human plasma proteome http://plasmaproteomedatabase.org/index.html accessed on 10 September 2022 [284]. These data include many proteome parameters: their dynamics, different protein presence, abundance, modifications, variations, etc. In the case of cancer, a proteome performs multiple perturbations, where all its components are involved through changes in their levels and modifications. Here, the plasma proteome works as a united entity that executes and reflects the processes in the human body. Accordingly, the profiling of plasma proteomes is a promising and powerful approach to follow these processes. This profiling could combine hundreds of already known plasma biomarkers and has a very promising future in biomedicine as it could disclose information about any abnormal situation in the human body including cancer. There is a big chance that MS-based proteomics will become a part of the routine medical technique [14,285]. In addition to the usual MS analysis of proteins/proteoforms, this technique should include special processing programs allowing conclusions to be made about the human body’s state based on these variations in protein/proteoform signatures/profiles (level, PTMs, etc.). In our work, we collected information about the connection of cancers with levels of “classical plasma proteins” and generated their proteoform profiles (Table 1, Supplementary Figure S1). As a next step, similar profiles representing protein perturbations in plasma produced in the case of different cancers should be generated. Moreover, based on this information, different test systems can be developed.

## Figures and Tables

**Table 1:** The most abundant plasma proteins related to cancer. Concentration (µg/mL) is presented according to [16] if otherwise not shown. Abundance (emPAI) was calculated according to data from the semi-virtual 2DE (Supplementary Table S1). In the column “Cancer”, the references for cancer-related data are shown (the details are in the Supplementary File).

N.	UniProt	ID (UniProt)	UniProt Name (*Gene*)	pI/Mw	Leve µg/mL µg/mL	EmPAI	Cancer
1	P02763	A1AG1_HUMAN	Alpha-1-acid glycoprotein 1 (*ORM1*)	5.11/21,588	220	116.0	[32,33,34,35,36,37,38,39,40]
2	P19652	A1AG2_HUMAN	Alpha-1-acid glycoprotein 2 (*ORM2*)	5.12/21,651	220	75.0	[33,41,42]
3	P01009	A1AT_HUMAN	Alpha-1-antitrypsin (*SERPINA1*)	5.37/44,325	350	38.0	[34,43,44,45,46,47,48,49,50]
4	P04217	A1BG_HUMAN	Alpha-1B glycoprotein (*A1BG*)	5.63/51,922	50	44.0	[32,51,52,53]
5	P01023	A2MG_HUMAN	Alpha-2-Macroglobulin (*A2M*)	5.98/160,810	220	112.0	[27,54,55,56]
6	P08697	A2AP_HUMAN	Alpha-2-antiplasmin (*SERPINF2*)	5.87/50,451	12	36.0	[57,58]
7	P02750	A2GL_HUMAN	Leucine-rich alpha-2-glycoprotein (*LRG1*)	5.66/34,346	2.7	17.0	[59,60,61]
8	P01011	AACT_HUMAN	Alpha 1-antichymotrypsin (*SERPINA3*)	5.32/45,266	110	151.0	[62,63,64]
9	Q15848	ADIPO_HUMAN	Adiponectin (*ADIPOQ*)	5.46/24,544	0.12	2.4	[65]
10	P43652	AFAM_HUMAN	Afamin (*AFM*)	5.58/66,577	320	39.0	[66,67,68,69,70]
11	P02768	ALBU_HUMAN	Albumin (*ALBU*)	5.67/66,472	1600	1207.0	[71,72,73,74,75]
12	P02760	AMBP_HUMAN	Protein AMBP (*AMBP*) Alpha-1-microglobulin Bikunin	5.76/37,115 6.13/20,847 4.89/15,974	48	28.0	[56,76,77]
13	P01019	ANGT_HUMAN	Angiotensinogen (*AGT*)	5.60/49,761	11	46.0	[37]
14	P01008	ANT3_HUMAN	Antithrombin-III (*SERPINC1*)	5.95/49,039	60	155.0	[48,78,79]
15	P02647	APOA1_HUMAN	Apolipoprotein A-I (*APOAI*)	5.27/28,079	310	354.0	[32,80,81,82,83,84,85,86]
16	P02652	APOA2_HUMAN	Apolipoprotein A-II (*APOA2*)	5.05/8708	750	285.0	[70,80,87,88,89,90,91]
17	P06727	APOA4_HUMAN	Apolipoprotein A-IV (*APOA4*)	5.18/43,376	32	78.5	[68,82,92,93]
18	P04114	APOB_HUMAN	Apolipoprotein B-100 (*APOB*)	6.57/512,858	33	21.0	[52,69,94]
19	P02654	APOC1_HUMAN	Apolipoprotein C1 (*APOC1*)	7.93/6631	77	8.3	[95,96]
20	P02655	APOC2_HUMAN	Apolipoprotein C-II (*APOC2*)	4.58/8204	240	6.1	[20,32]
21	P02656	APOC3_HUMAN	Apolipoprotein C-III (*APOC3*)	4.72/8765	170	6.1	[32,69,91,95]
22	P05090	APOD_HUMAN	Apolipoprotein D (*APOD*)	5.20/19,303	82	16.8	[32,97]
23	P02649	APOE_HUMAN	Apolipoprotein E (*APOE*)	5.52/34,237	14	52.2	[69,97,98,99,100,101]
24	Q13790	APOF_HUMAN	Apolipoprotein F (*APOF*)	4.40/17,425	4.1	2.0	[102]
25	P02749	APOH_HUMAN	Beta-2-glycoprotein 1 (*APOH*)	8.37/36,255	78	0.1	[103,104,105,106,107]
26	O95445	APOM_HUMAN	Apolipoprotein M (*APOM*)	5.66/21,253	1.5	7.9	[108]
27	P02747	C1QC_HUMAN	Complement C1q subcomponent subunit C (*C1QC*)	8.33/22,813	0.91	1.8	[109]
28	P00736	C1R_HUMAN	Complement C1r subcomponent (*C1R*)	5.76/78,213	4.3	9.7	[110,111]
29	P09871	C1S_HUMAN	Complement C1s subcomponent (*C1S*)	4.85/74,887	5.2	5.6	[112,113]
30	P05156	CFAI_HUMAN	Complement factor I (*CFI*)	7.38/63,487	0.006	11.9	[49,114]
31	P00751	CFAB_HUMAN	Complement factor B (*CFB*)	6.66/83,001	95 [115]	1.14	[58,69,116,117]
32	P00746	CFAD_HUMAN	Complement factor D (*CFD*)	6.85/24,405	2.9	7.1	[118]
33	P08603	CFAH_HUMAN	Complement factor H (*CFH*)	6.12/137,053	57	0.24	[32,119,120,121]
34	P06681	CO2_HUMAN	Complement C2 (*C2*)	7.57/81,085	35	20.5	[32]
35	P01024	CO3_HUMAN	Complement C3 (*C3)*	6.00/184,951	260	31.1	[58,93,116,122,123,124,125]
36	P0C0L4	CO4A_HUMAN	Complement C4-A (*C4A*)	6.60/190,534	63 [115]	36.3	[52,124,125]
37	P0C0L5	CO4B_HUMAN	Complement C4-B (*C4B*)	6.83/190,500	90	37.8	[124,125]
38	P01031	CO5_HUMAN	Complement C5 (*C5*)	6.07/186,341	95	35.7	[69,126]
39	P13671	CO6_HUMAN	Complement C6 (*C6*)	6.17/102,412	3.7	15.7	[32,52]
40	P10643	CO7_HUMAN	Complement C7 (*C7*)	6.09/91,115	2.6	17	[69,127]
41	P02748	CO9_HUMAN	Complement component C9 (*C9*)	5.42/60,979	5.2	11.8	[128,129,130]
42	P00915	CAH1_HUMAN	Carbonic anhydrase (*CA1*)	6.63/28,739	0.59	2.5	[131,132]
43	P08185	CBG_HUMAN	Corticosteroid-binding globulin *(SERPINA6*)	5.64/42,639	1.2	27.9	[133]
44	P15169	CBPN_HUMAN	Carboxypeptidase N catalytic chain (*CPN1)*	6.88/50,034	0.72	6.4	[134]
45	P08571	CD14_HUMAN	Monocyte differentiation antigen CD14 (*CD14*) urinary form	5.58/37,215	0.42	4.5	[135,136]
46	P00450	CERU_HUMAN	Ceruloplasmin (*CP*)	5.41/120,085	86	86.7	[34,54,137,138,139,140,141,142]
47	P06276	CHLE_HUMAN	Cholinesterase (*BCHE*)	6.33/65,084	0.17	2.97	[143]
48	P10909	CLUS_HUMAN	Clusterin (*CLU*)	5.89/50,063	25	29.9	[32,52,58,69,144,145,146,147,148,149]
49	Q96KN2	CNDP1_HUMAN	Beta-Ala-His dipeptidase (*CNDP1*)	5.08/53,864	0.23	2.7	[150,151,152,153]
50	P22792	CPN2_HUMAN	Carboxypeptidase N subunit 2 *(CPN2)*	5.54/58,227	2	6.1	[32]
51	P02741	CRP_HUMAN	C-reactive protein (*CRP*) C-reactive protein (1-205)	5.28/23,047 5.28/22,950	0.26	1.0	[154,155]
52	Q16610	ECM1_HUMAN	Extracellular matrix protein 1 (*ECM1*)	6.19/58,812	0.77	9.6	[156,157,158]
53	P23142	FBLN1_HUMAN	Fibulin-1 (*FBLN1*)	5.03/74,291	0.62	11.8	[159,160,161,162,163]
54	O75636	FCN3_HUMAN	Ficolin-3 *(FCN3*)	6.22/30,354	1	11.8	[164,165,166,167,168]
55	P02765	FETUA_HUMAN	Alpha-2-HS-glycoprotein (*AHSG*)	4.53/30,238	82	30.6	[169,170]
56	Q9UGM5	FETUB_HUMAN	Fetuin-B *(FETUB)*	6.52/40,488	0.27	1.8	[171]
57	P02671	FIBA_HUMAN	Fibrinogen alpha chain (*FGA*)	5.79/91,359	0.13	10.9	[32,69,172,173]
58	P02675	FIBB_HUMAN	Fibrinogen beta chain (*FGB)*	7.95/50,763	130	62.5	[32,173,174]
59	P02679	FIBG_HUMAN	Fibrinogen gamma chain (*FGG*)	5.24/48,483	98	39.2	[32,69,175,176,177,178]
60	P02751	FINC_HUMAN	Fibronectin (*FN1*)	5.25/269,259	20	14.1	[48,94,179,180,181,182]
61	P06396	GELS_HUMAN	Plasma gelsolin (*GSN*)	5.72/82,959	16	23.4	[166,183,184]
62	P22352	GPX3_HUMAN	Glutathione peroxidase 3 (*GPX3*)	7.85/23,464	10	11.7	[185]
63	P69905	HBA_HUMAN	Hemoglobin subunit alpha (*HBA1*)	8.73/15,126	41	1129	[54]
64	P68871	HBB_HUMAN	Hemoglobin subunit beta (*HBB*)	6.81/15,867	30	847.0	[54,186]
65	P02790	HEMO_HUMAN	Hemopexin (*HPX*)	6.43/49,295	180	165.0	[177,187,188,189]
66	P05546	HEP2_HUMAN	Heparin Cofactor 2 (*SERPIND1*)	6.26/54,960	4.3	43.0	[58,189,190,191,192]
67	P00738	HPT_HUMAN	Haptoglobin (Zonulin) (*HP*) haptoglobin alpha 1 chain haptoglobin alpha 2 chain haptoglobin beta chain	6.13/43,349 5.23/93,55 5.57/15,946 6.32/27,265	210	323.0	[166,193,194,195,196,197,198,199]
68	P00739	HPTR_HUMAN	Haptoglobin-related protein (*HPR)*	6.63/39,030	41 [200]	105.0	[201]
69	P04196	HRG_HUMAN	Histidine-rich glycoprotein (*HRG*)	7.03/57,660	35	24.0	[202,203]
70	P05155	IC1_HUMAN	Plasma protease C1 inhibitor (*SERPING1*)	5.97/52,843	12	9.4	[204,205]
71	P19827	ITIH1_HUMAN	Inter-alpha-trypsin inhibitor heavy chain H1 (*ITIH1*)	6.33/71,415	24	25.0	[29,206,207,208,209,210]
72	Q06033	ITIH3_HUMAN	Inter-alpha-trypsin inhibitor heavy chain H3 (*ITIH3*)	5.01/69,360	2	7.7	[207]
73	Q14624	ITIH4_HUMAN	Inter-alpha-trypsin inhibitor heavy chain H4 (*ITIH4*)	5.92/70,586	42	41.6	[29,207]
74	P29622	KAIN_HUMAN	Kallistatin (*SERPINA4*)	7.88/46,355	1.1	81.8	[211]
75	P01042	KNG1_HUMAN	Kininogen 1 (*KNG1*)	6.23/69,897	28	7.7	[212,213,214]
76	P04180	LCAT_HUMAN	Phosphatidylcholine-sterol acyltransferase (*LCAT*)	5.71/47,084	0.22	1.8	[171]
77	P51884	LUM_HUMAN	Lumican *(LUM)*	6.17/36,661	4	6.4	[166,215,216,217]
78	P11226	MBL2_HUMAN	Mannose-binding protein C (*MBL2*)	5.40/24,021	0.07	6.4	[171,218,219]
79	P36955	PEDF_HUMAN	Pigment epithelium-derived factor (*SERPINF1*)	5.90/44,388	7.2	14.5	[220]
80	Q96PD5	PGRP2_HUMAN	N-acetylmuramoyl-L-alanine amidase (*PGLYRP2*)	7.64/59,980	14	4.0	[171,221,222]
81	P80108	PHLD_HUMAN	Phosphatidylinositol-glycan-specific phospholipase D (*GPLD1*)	5.78/89,811	4	3.7	[32,223,224]
82	P00747	PLMN_HUMAN	Plasminogen (*PLG*) Plasmin heavy chain A Angiostatin Plasmin heavy chain A, short form Plasmin light chain	7.08/88,432 6.79/63,245 8.30/44,053 7.44/54,341 7.67/25,205	25	81.0	[225,226]
83	P27169	PON1_HUMAN	Serum paraoxonase/arylesterase 1 (*PON1*)	5.08/39,600	7.7	43.4	[79,227,228,229,230,231,232]
84	P27918	PROP_HUMAN	Properdin (*CFP*)	8.33/48,494	0.33	1.7	[233]
85	P07225	PROS_HUMAN	Vitamin K-dependent protein S (*PROS1*)	5.17/70,645	1.7	7.7	[234]
86	P02753	RET4_HUMAN	Plasma retinol-binding protein 4 (*PRBP*)	5.27/21,072	580	39.3	[235,236,237]
87	P0DJI8	SAA1_HUMAN	Serum amyloid A-1 (*SAA1*)	5.89/11,683	7.4	4.3	[32,34,238,239,240]
88	P02743	SAMP_HUMAN	Serum amyloid P-component (*APCS*)	6.12/23,259	8.7	39.5	[241]
89	P04278	SHBG_HUMAN	Sex hormone-binding globulin (*SHBG*)	5.83/40,468	0.26	5.8	[242,243]
90	P05109	S10A8_HUMAN	Protein S100-A8 (*S100A8*)	6.50/10,835	0.27	0.9	[244,245,246]
91	P06702	S10A9_HUMAN	Protein S100-A9 (*S100A9*)	5.71/13,242	1.9	2.6	[244,247]
93	P05452	TETN_HUMAN	Tetranectin (*CLEC3B*)	5.80/20,139	58	31.5	[32,248,249]
94	P05543	THBG_HUMAN	Thyroxine-binding globulin (*SERPINA7)*	5.76/44,102	1.3	12.5	[250,251]
95	P00734	THRB_HUMAN	Prothrombin *(F2)*	5.23/65,308	27	24.8	[252,253]
96	P02787	TRFE_HUMAN	Serotransferrin (*TF*)	6.70/75,195	360	41.8	[25,254]
97	P02766	TTHY_HUMAN	Transthyretin (*TTR*)	5.31/13,761	770	23.9	[32,95,177,255,256,257]
98	P02774	VTDB_HUMAN	Vitamin D-binding protein (*GC*)	5.16/51,197	57	181.4	[32,258,259]
99	P04004	VTNC_HUMAN	Vitronectin (*VTN*)	5.47/52,278	35	22.7	[25,32,52,260]
100	P25311	ZA2G_HUMAN	Zinc-alpha2-glycoprotein (*AZGP1*)	5.58/32,145	31	31.5	[261,262,263,264]

## Data Availability

Not applicable.

## Supplementary Materials

The following supporting information can be downloaded at: https://www.mdpi.com/article/10.3390/ijms231911113/s1.

## Abbreviations

MS	Mass spectrometry
ESI LC-MS/MS	Liquid chromatography–electrospray ionization tandem mass spectrometry
GBM	Glioblastoma multiform
2DE	Two-dimensional gel electrophoresis
emPAI	Exponentially modified protein abundance index
HCC	Hepatocellular carcinoma
CRC	Colorectal cancer
NSCLC	Non-small cell lung cancer
SCLC	Small cell lung carcinoma
HDL	High-density lipoproteins
cSCC	Cutaneous squamous cell carcinoma
BC	Breast cancer
OC	Ovarian cancer
PDAC	Pancreatic cancer
OSCC	Oral squamous cell carcinoma
GC	Gastric cancer
MM	Multiple myeloma
PC	Prostate cancer
