# Supplementary material for: Construction of 2DE Patterns of Plasma Proteins: Aspect of Potential Tumor Markers
Source: Int J Mol Sci. 2022 Sep 21;23(19):11113. doi: 10.3390/ijms231911113 (PMC9569744; doi:10.3390/ijms231911113)
Supplement: Supplementary file 1 [file ijms-23-11113-s001.zip › Suppl Figure S1.pdf]

<table>
<tr><th>N</th><th>Protein</th><th>Semi-virtual 2DE</th><th>2DE sectional analysis</th></tr>
<tr><td>1.</td><td>A1AG1</td><td>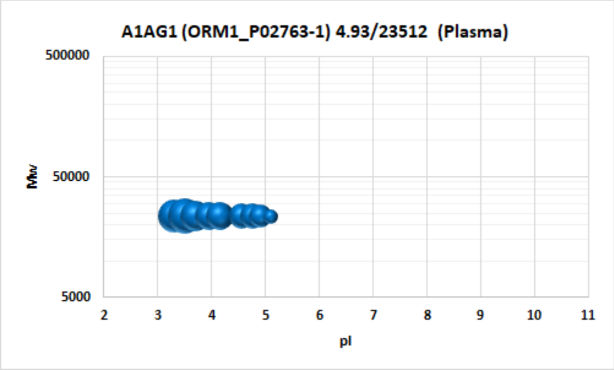
</td><td>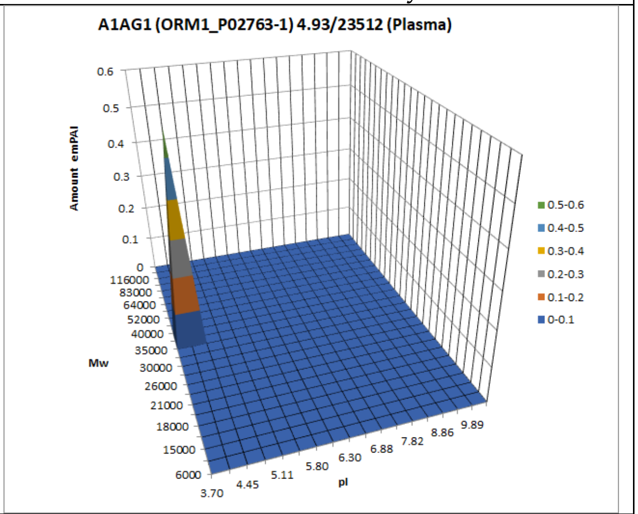
</td></tr>
<tr><td>2.</td><td>A1AG2</td><td>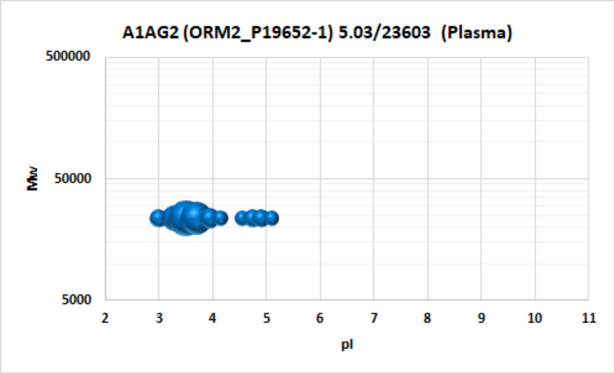
</td><td>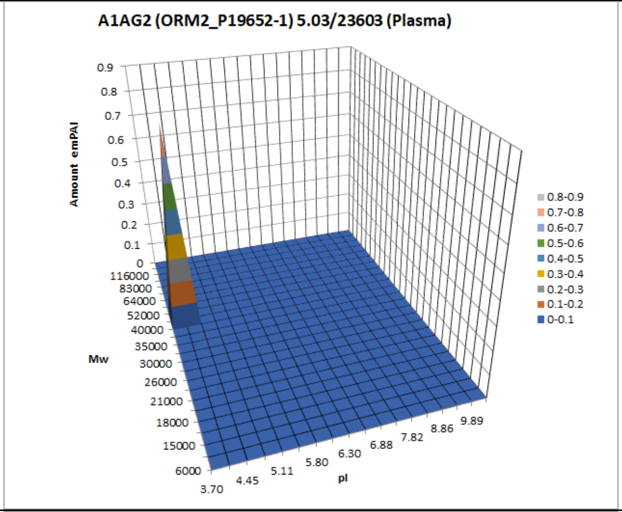
</td></tr>
<tr><td>3.</td><td>A1AT</td><td>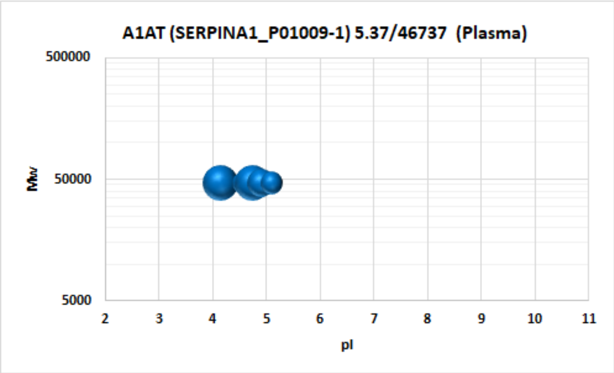
</td><td>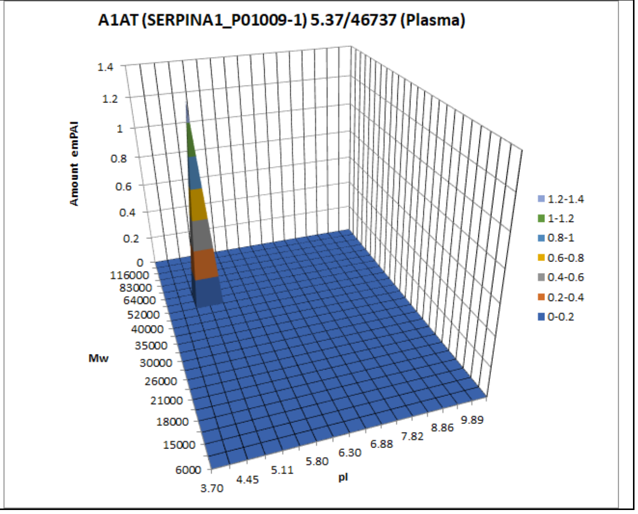
</td></tr>
</table>

4.	A1BG	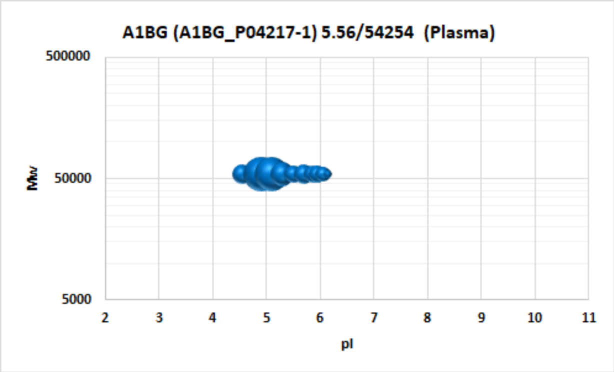	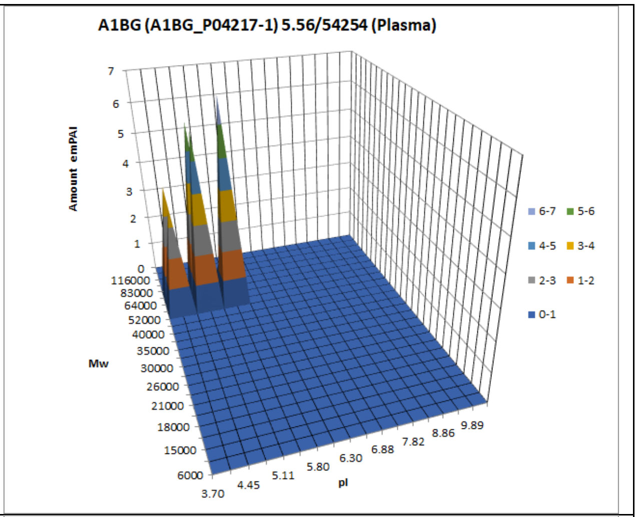
5.	A2AP	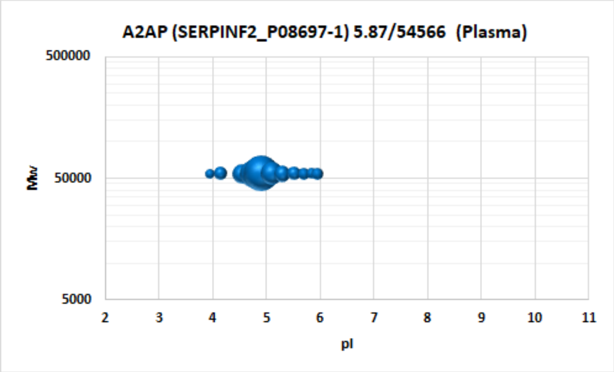	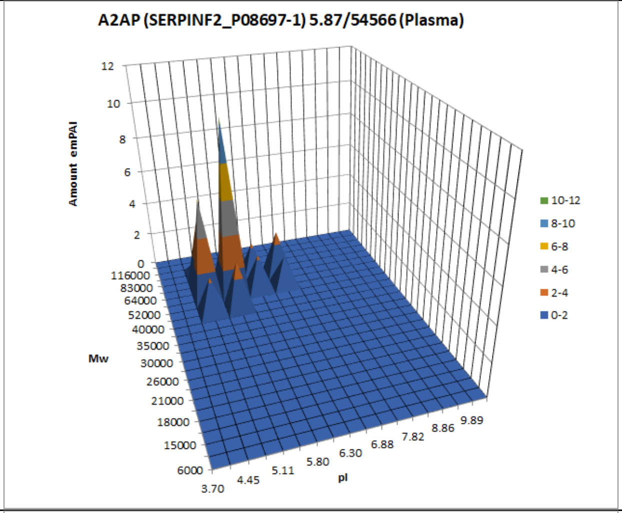
6.	A2GL	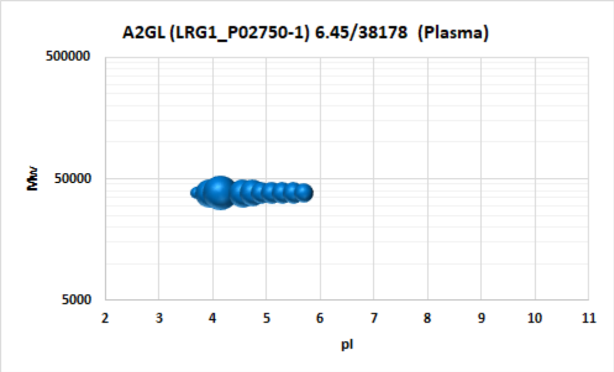	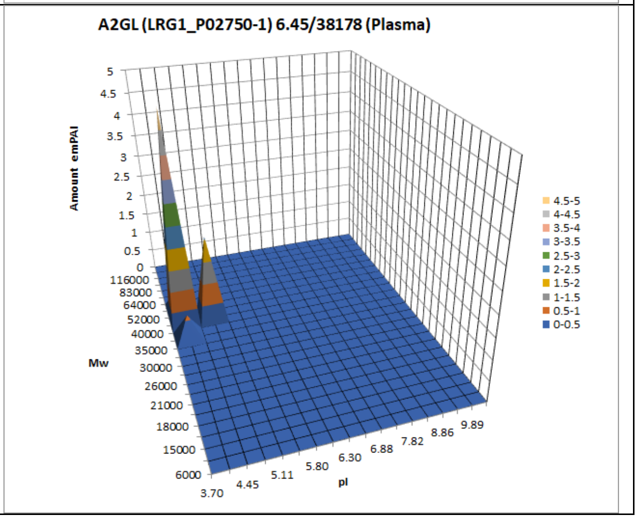

<table>
<tr>
<td>7.</td>
<td>A2MG</td>
<td>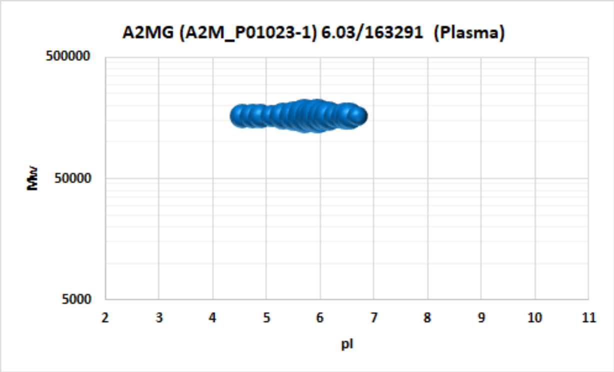
</td>
<td>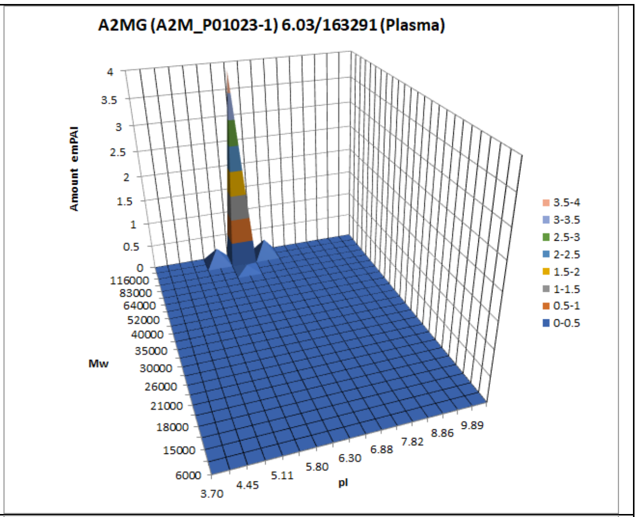
</td>
</tr>
<tr>
<td>8.</td>
<td>AACT</td>
<td>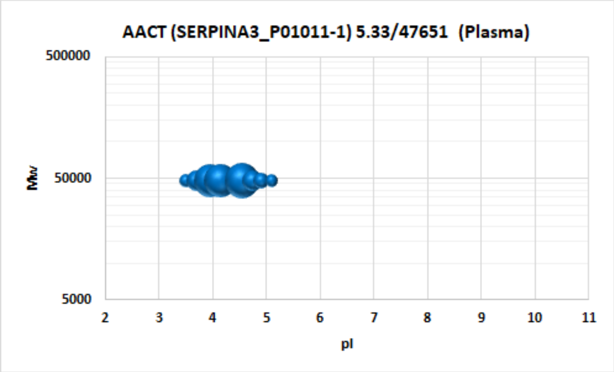
</td>
<td>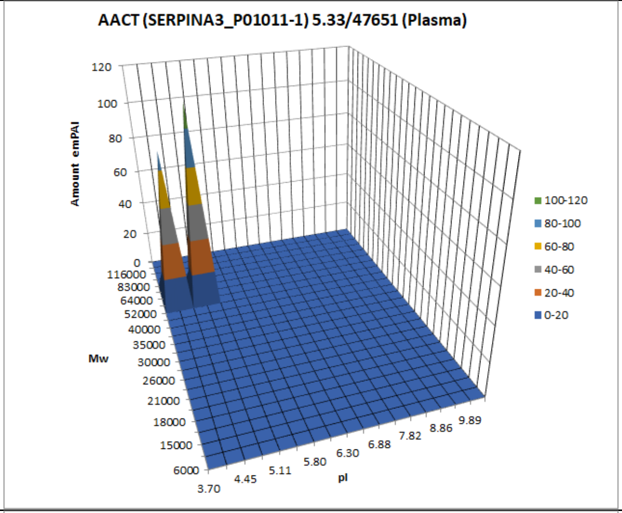
</td>
</tr>
<tr>
<td>9.</td>
<td>ADIPO</td>
<td>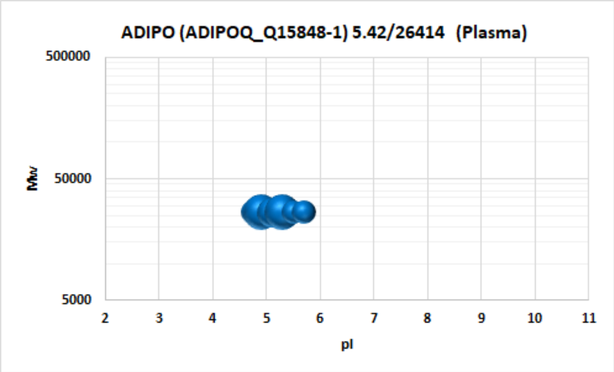
</td>
<td>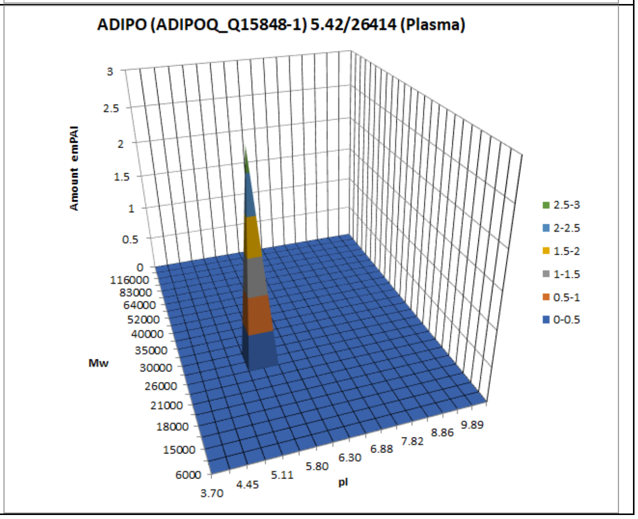
</td>
</tr>
</table>

<table>
<tr><td>10.</td><td>AFAM</td><td>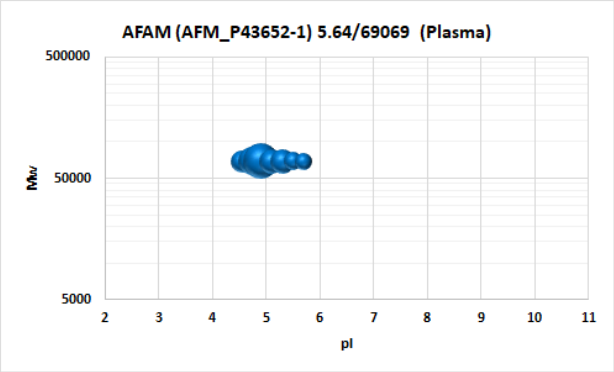
</td><td>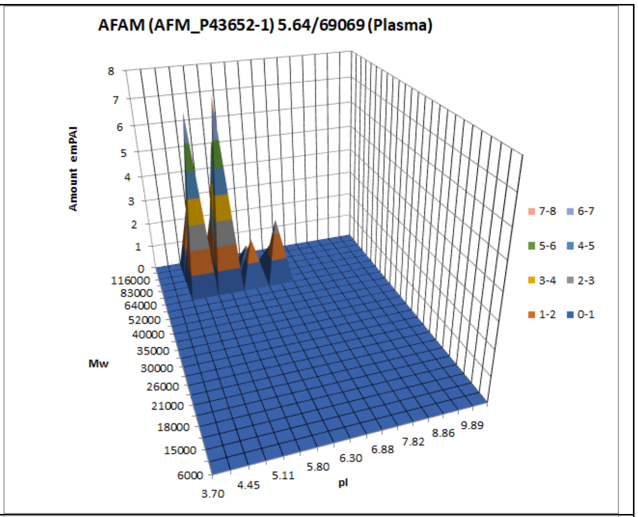
</td></tr>
<tr><td>11.</td><td>ALBU</td><td>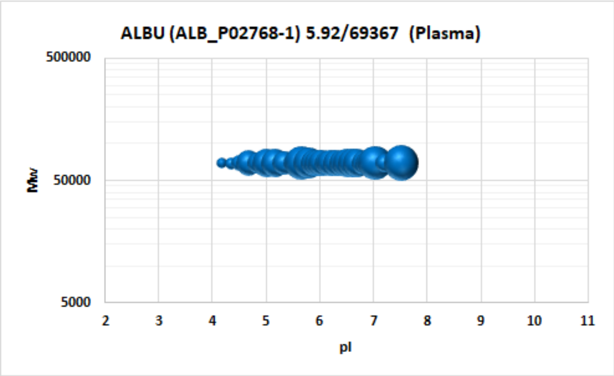
</td><td>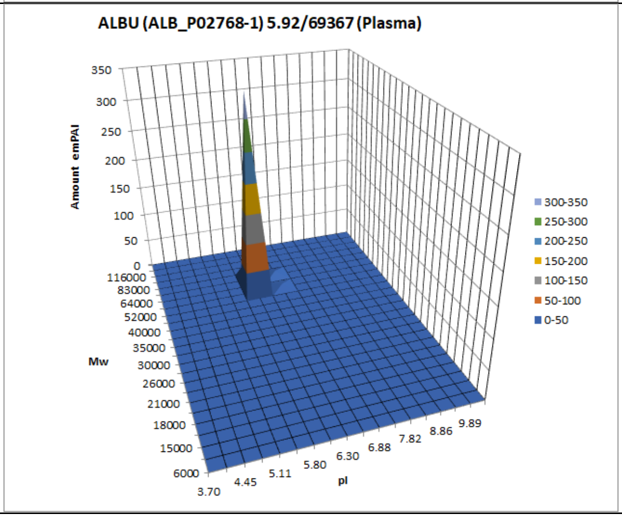
</td></tr>
<tr><td>12.</td><td>AMBP</td><td>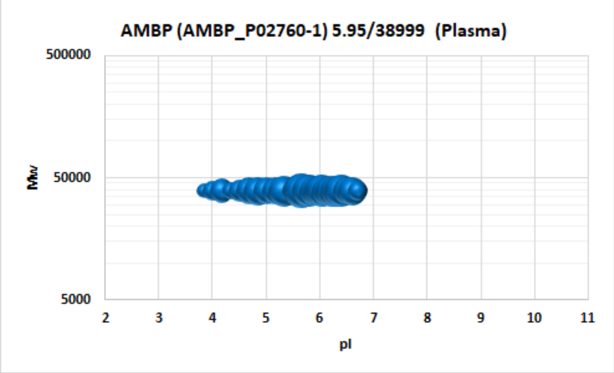
</td><td>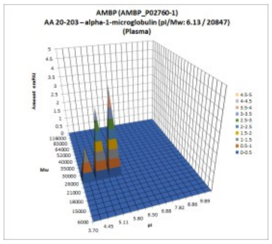

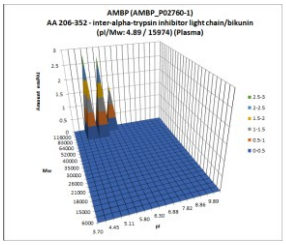
</td></tr>
</table>

13.	ANGT	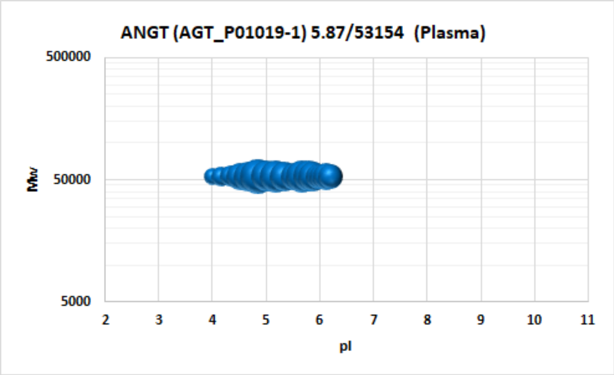	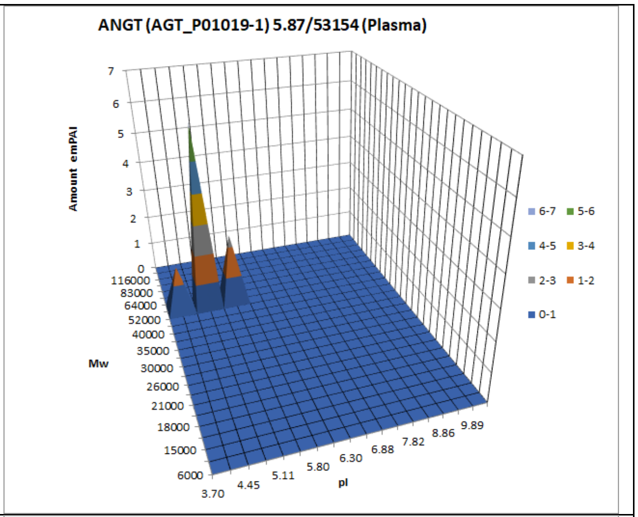
14.	ANT3	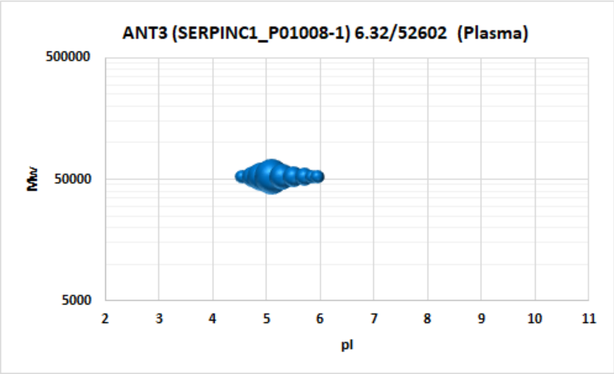	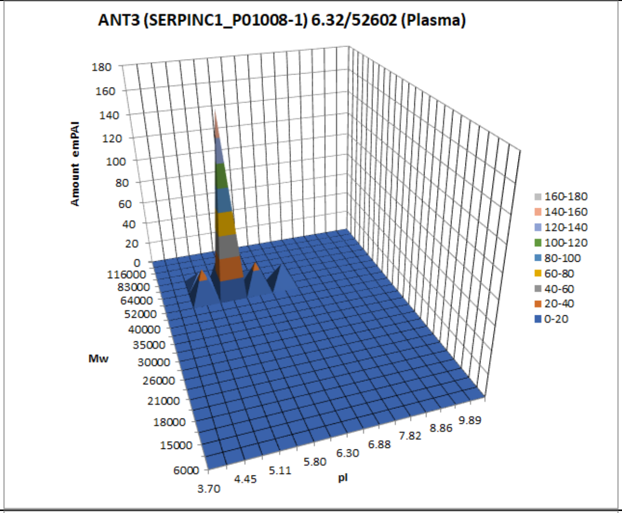
15.	APOA1	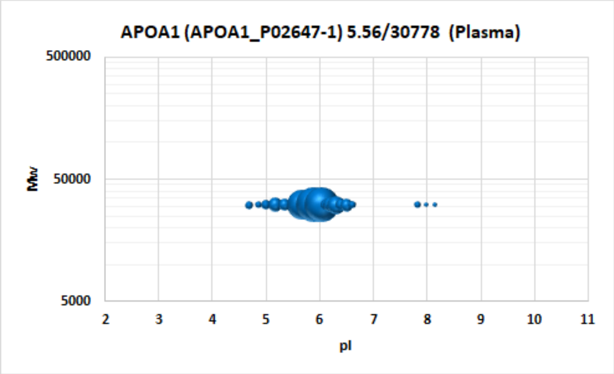	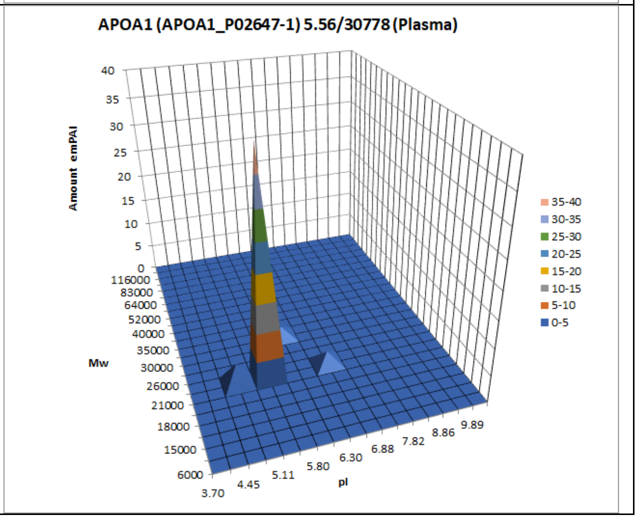

16.	APOA2	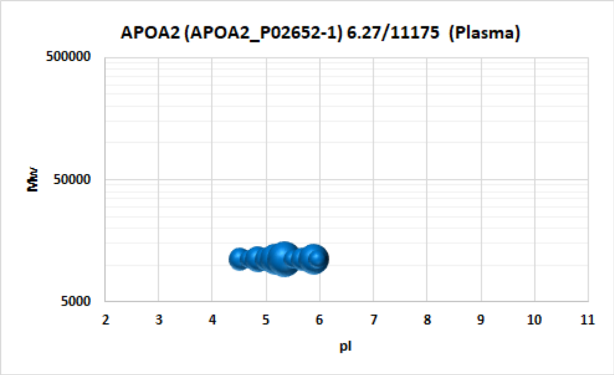	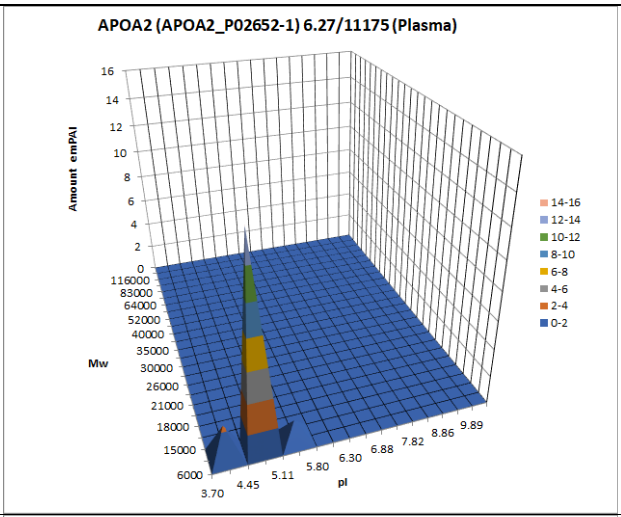
17.	APOA4	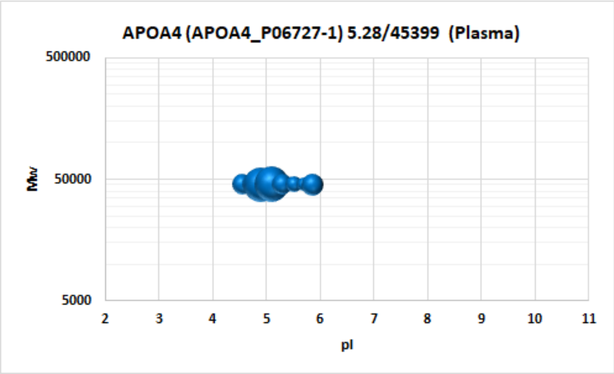	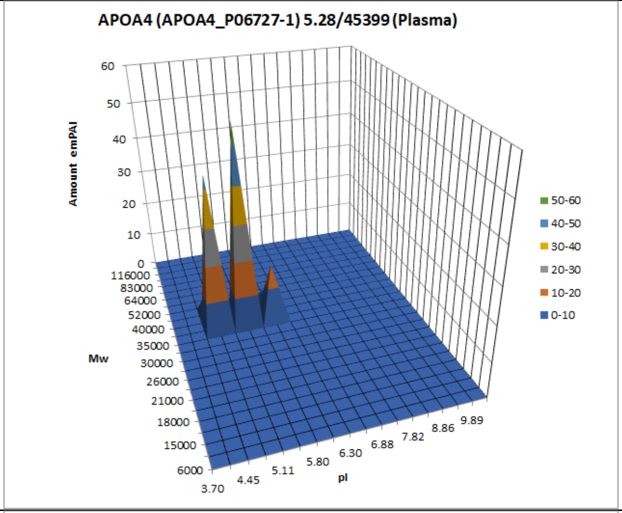
18.	APOB	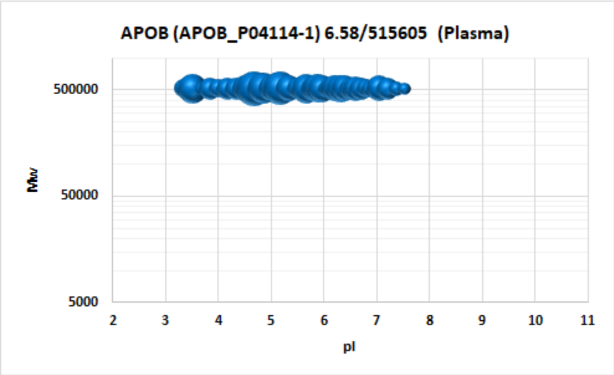	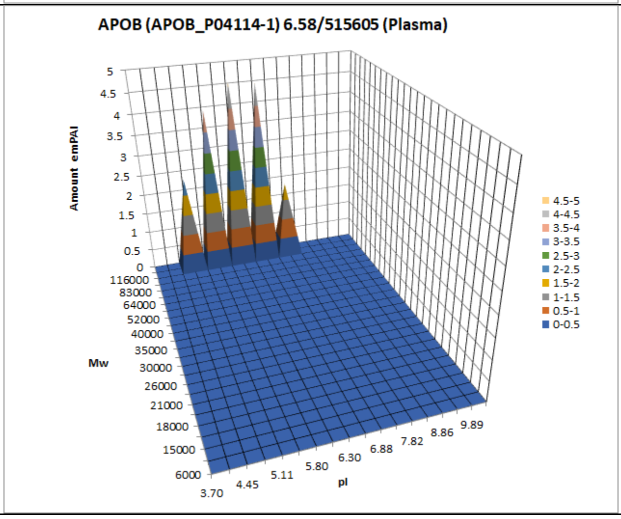

<table>
<tr>
<td>19.</td>
<td>APOC1</td>
<td>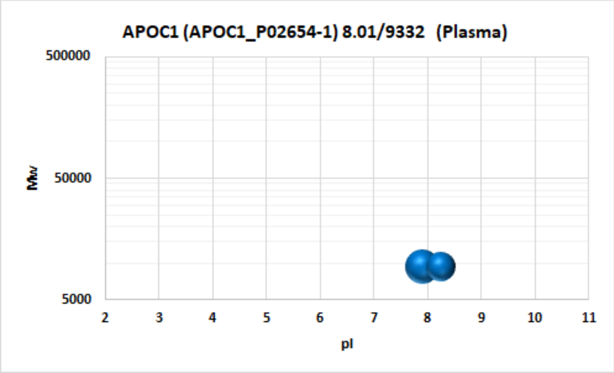
</td>
<td>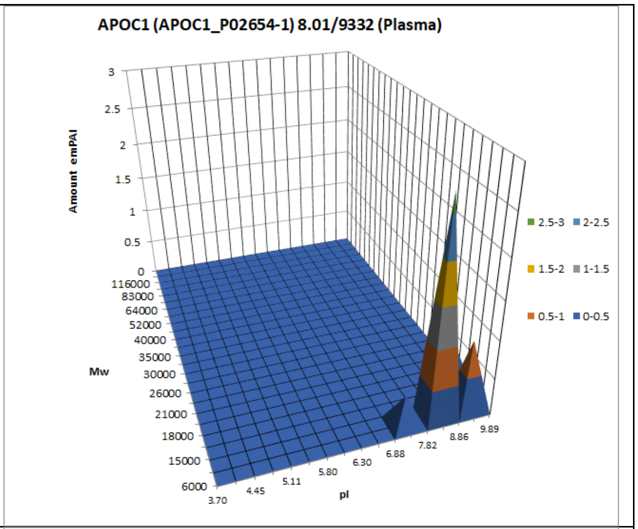
</td>
</tr>
<tr>
<td>20.</td>
<td>APOC2</td>
<td>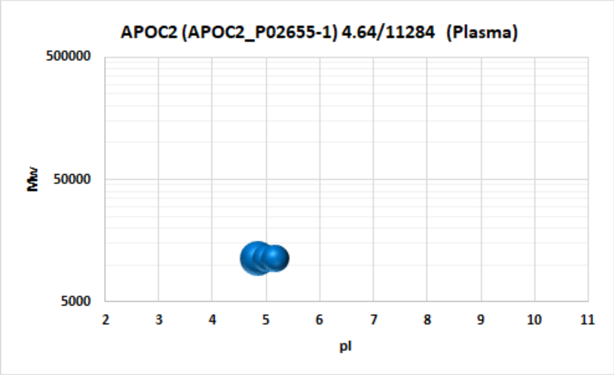
</td>
<td>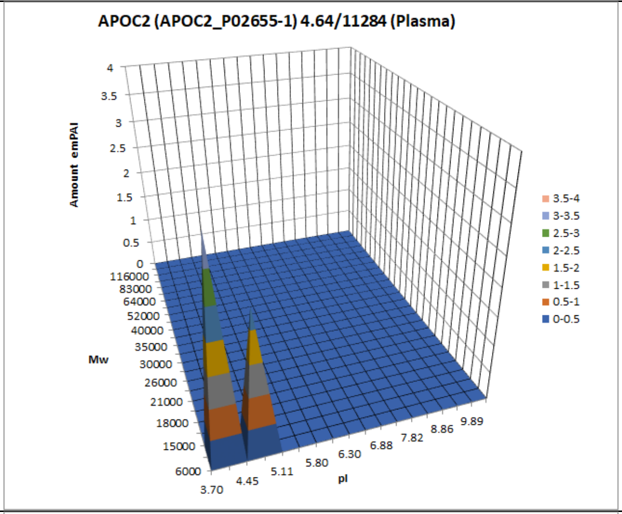
</td>
</tr>
<tr>
<td>21.</td>
<td>APOC3</td>
<td>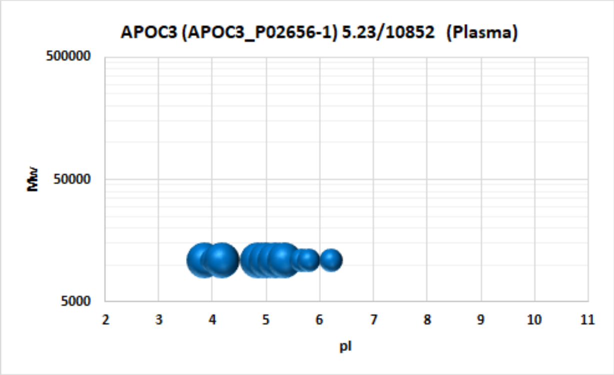
</td>
<td>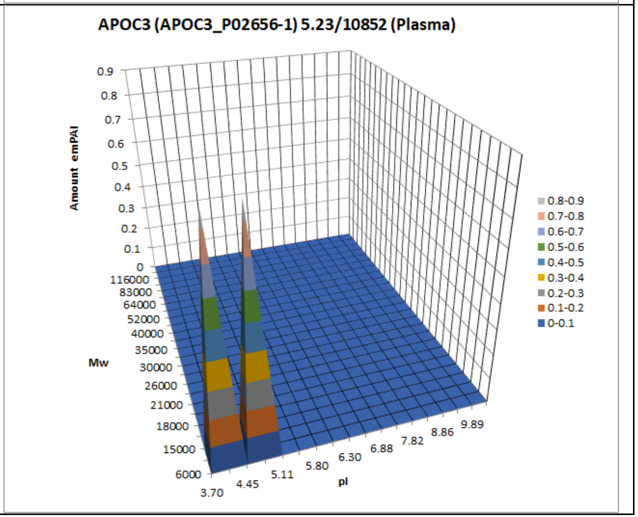
</td>
</tr>
</table>

22.	APOC4	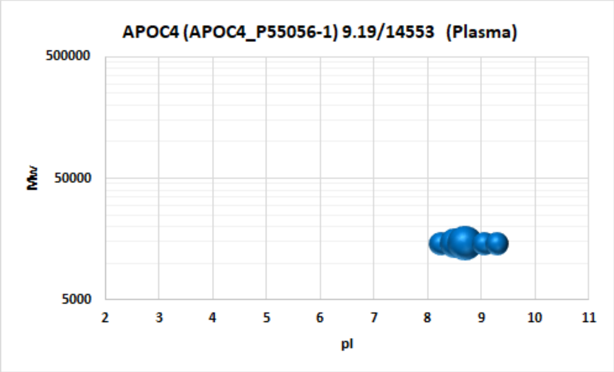	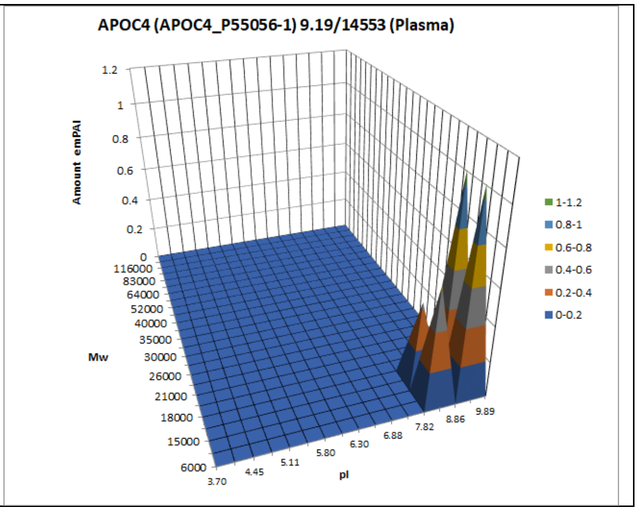
23.	APOD	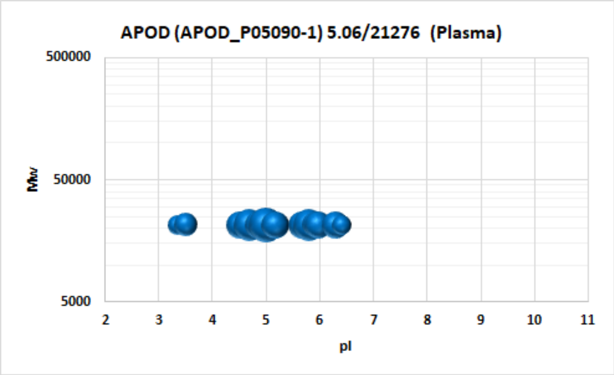	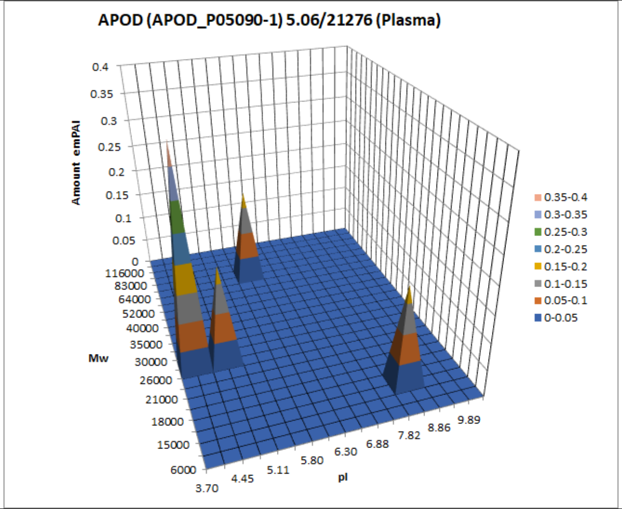
24.	APOE	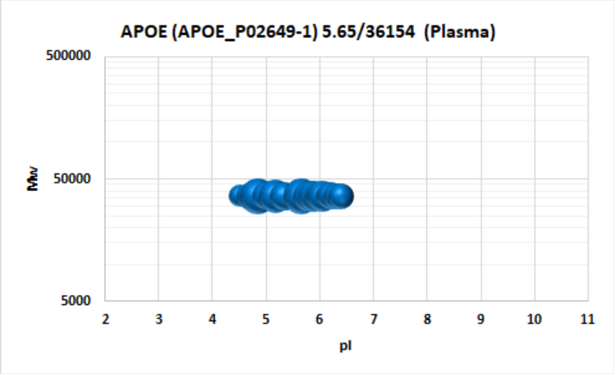	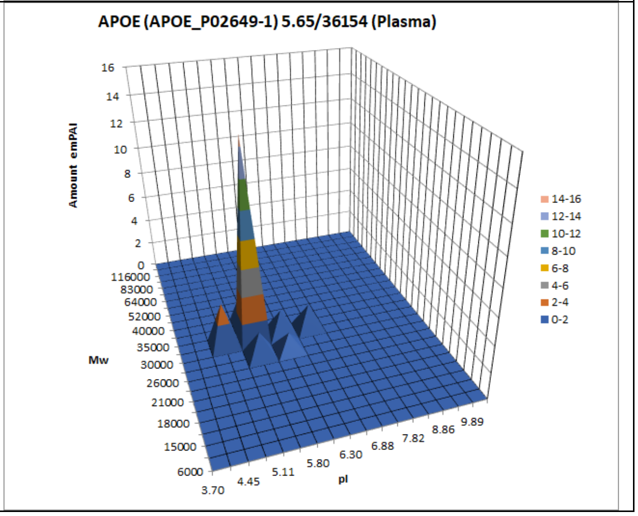

25.	APOF	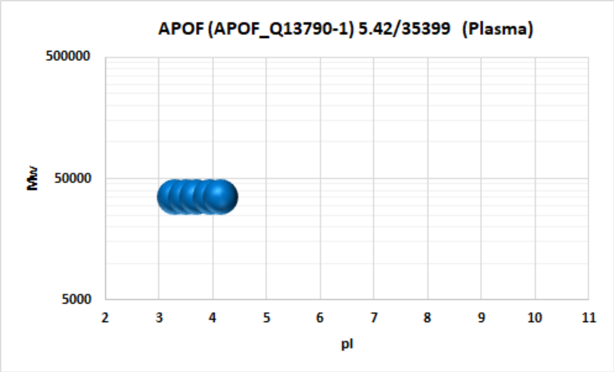	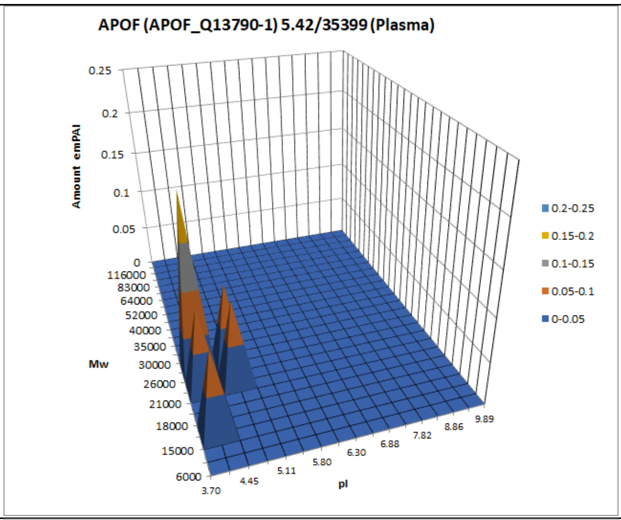
26.	APOH	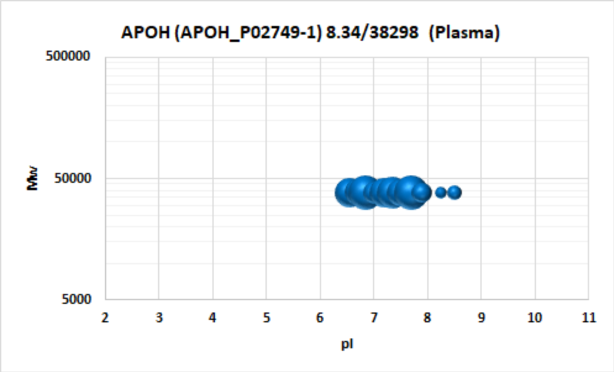	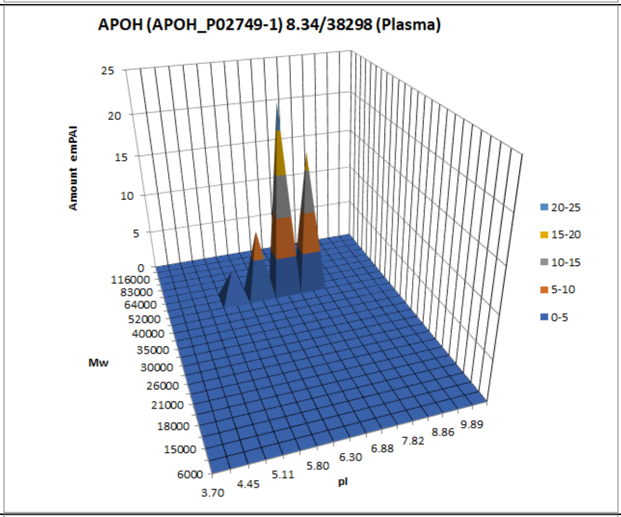
27.	APOL1	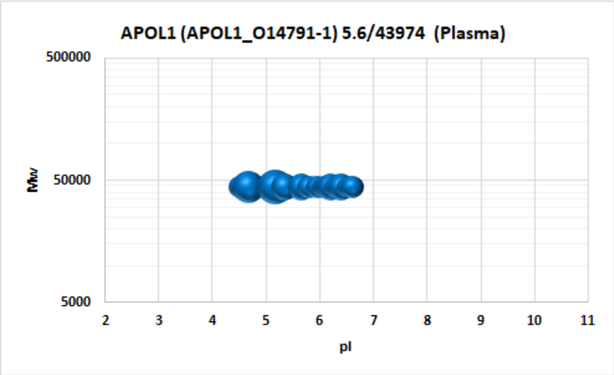	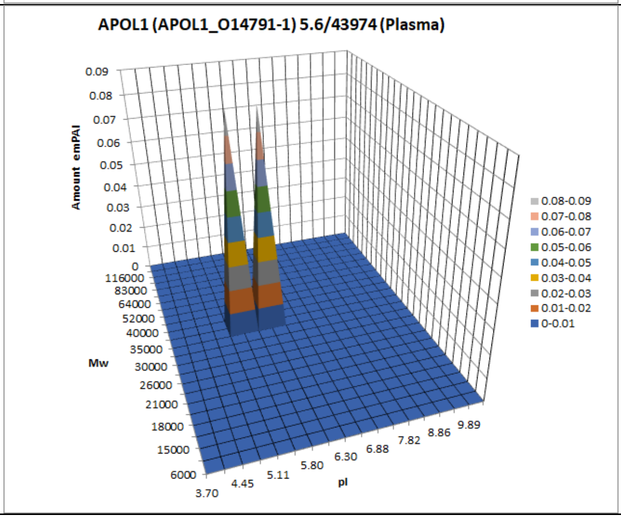

28.	APOM	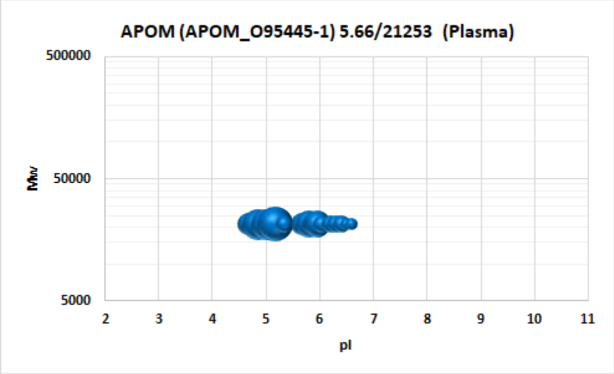	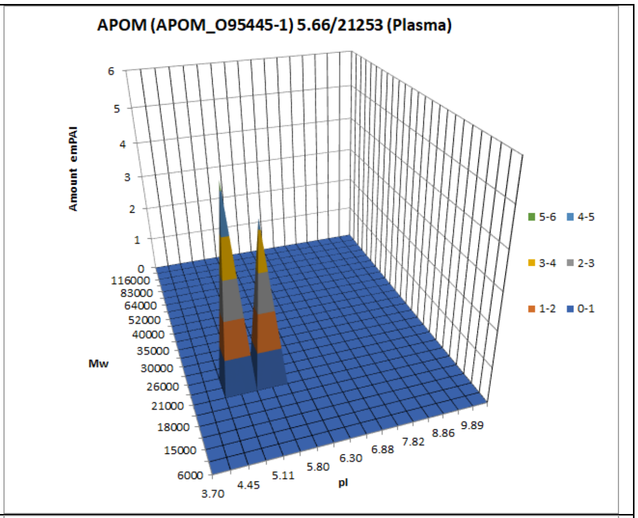
29.	C1QA	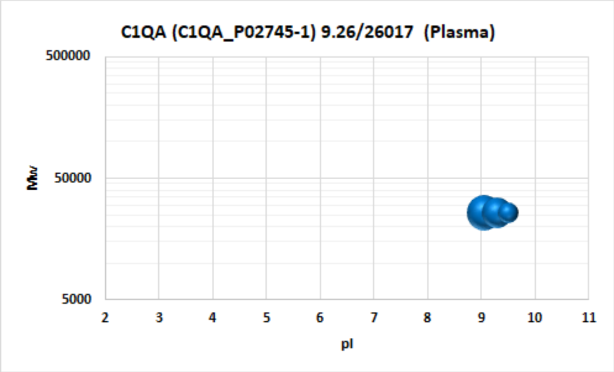	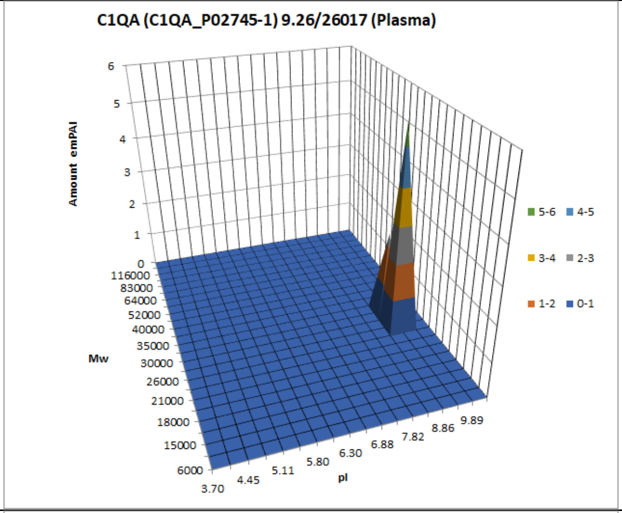
30.	C1QB	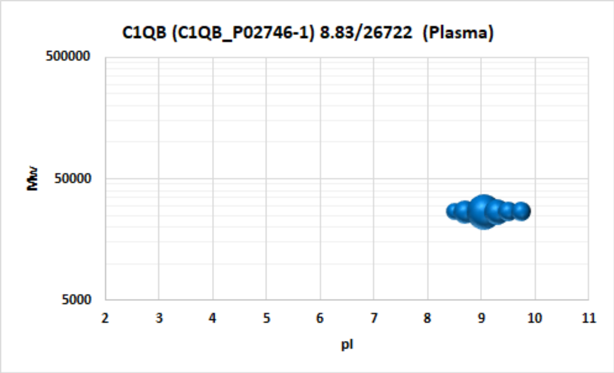	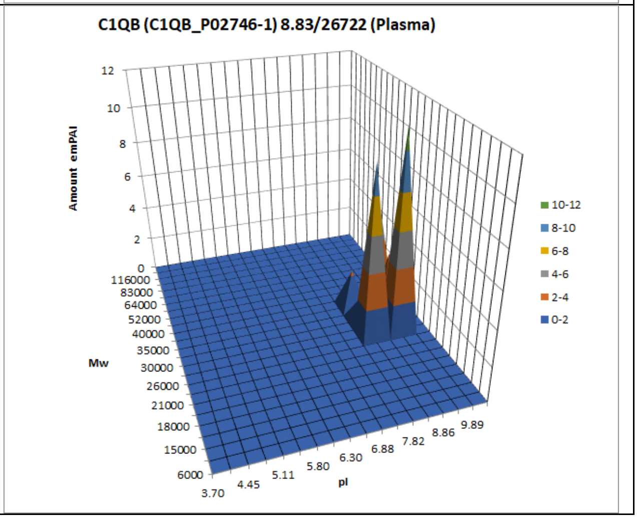

31.	C1QC	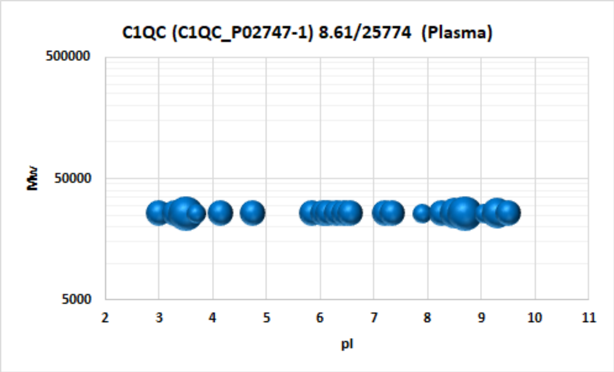	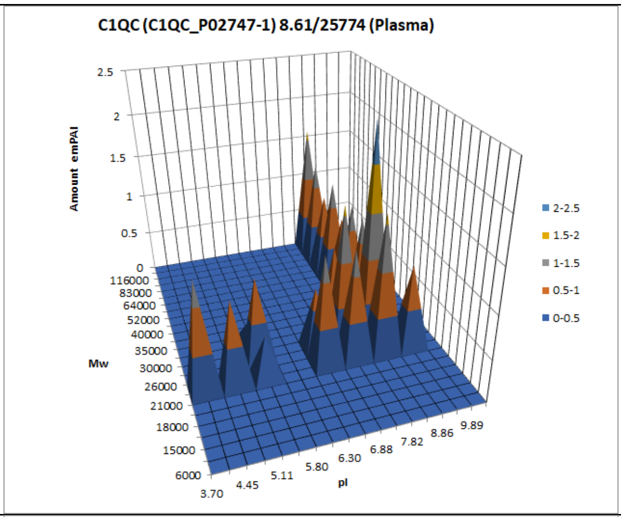
32.	C1R	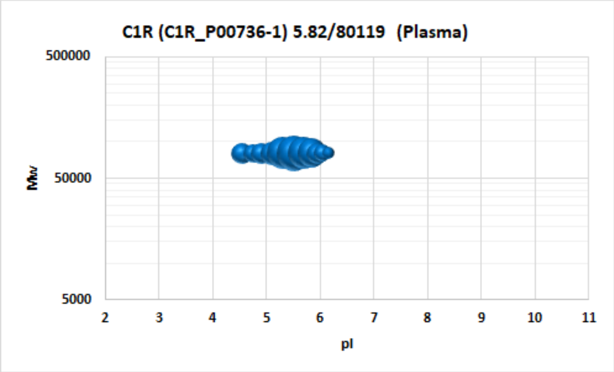	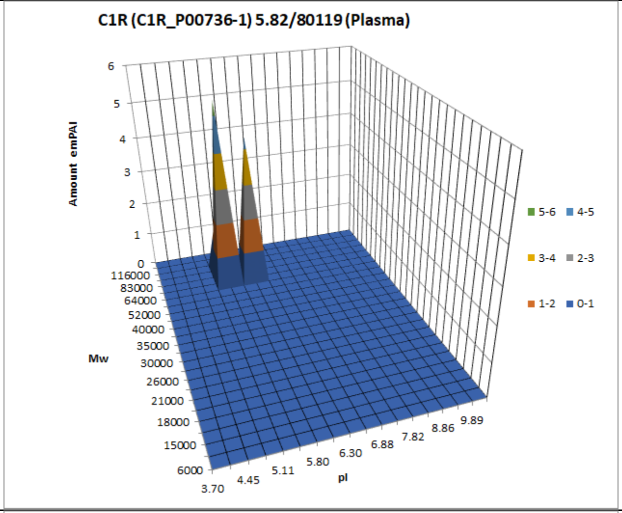
33.	C1S	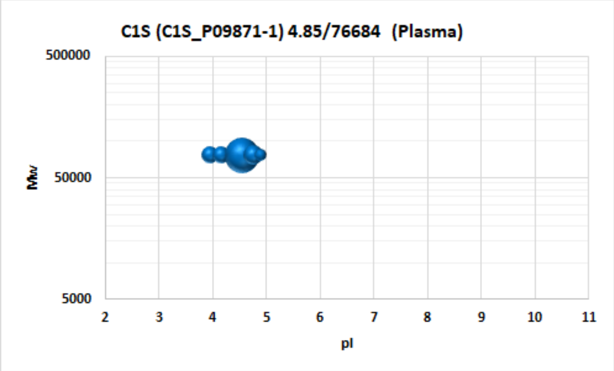	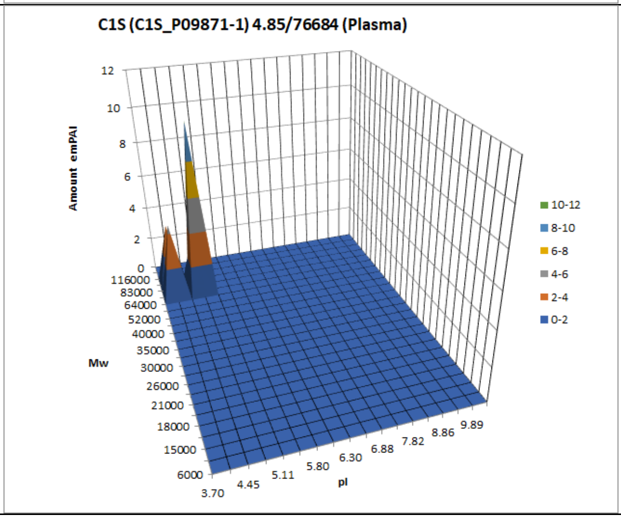

34.	C4BPA	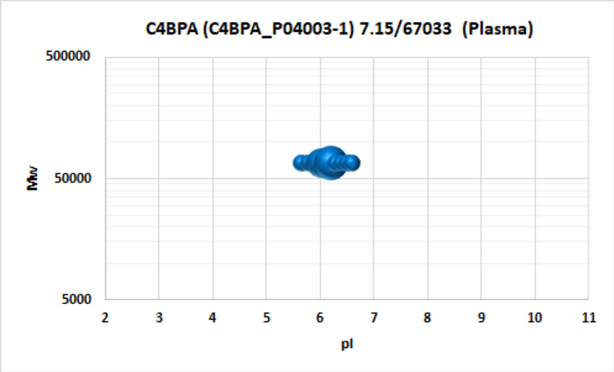	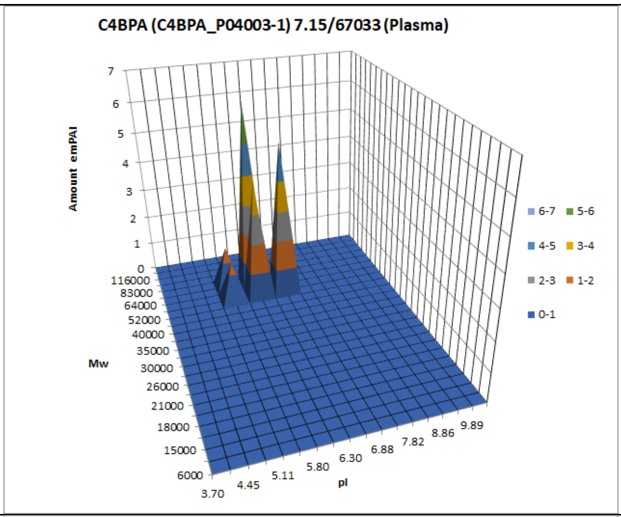
35.	CAH1	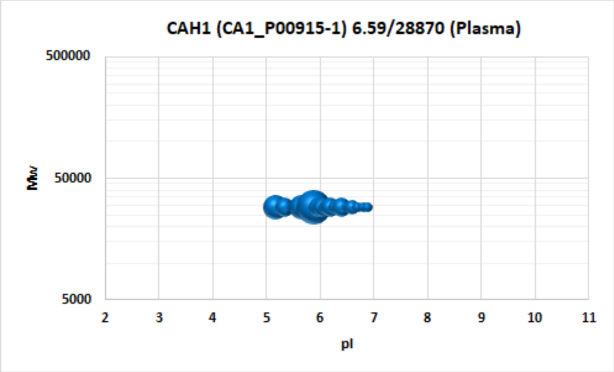	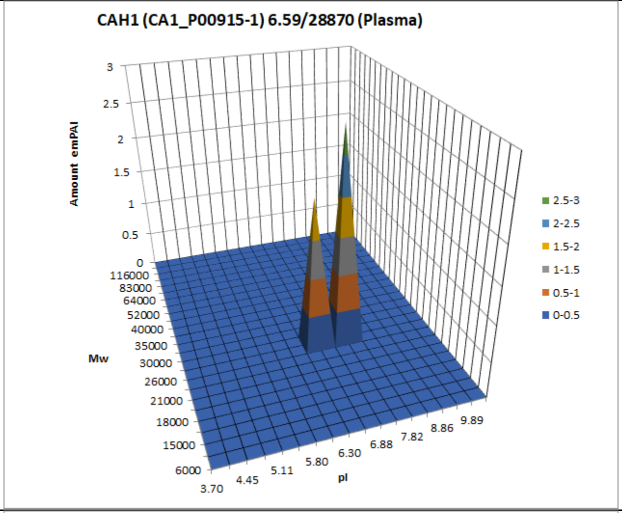
36.	CBG	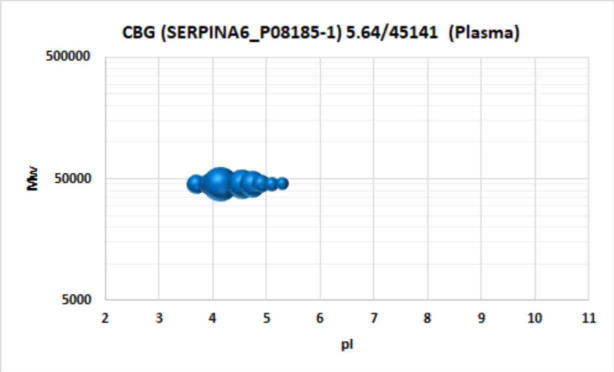	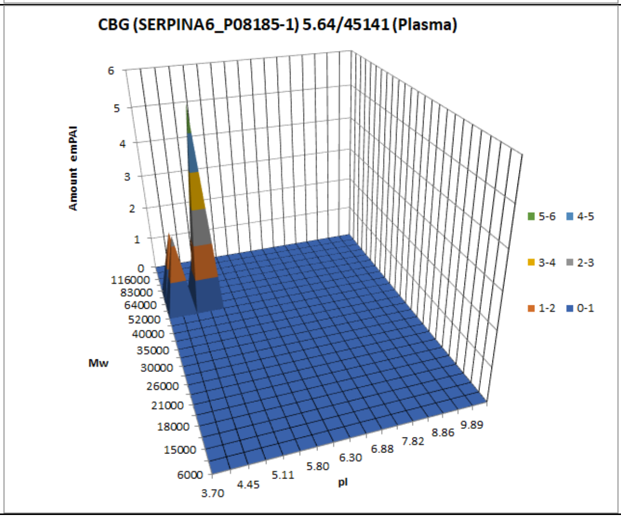

37.	CBPN	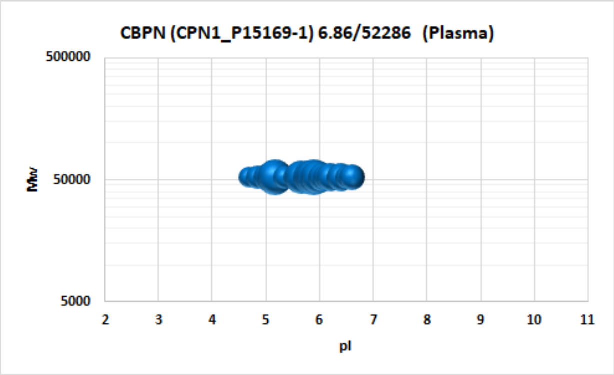	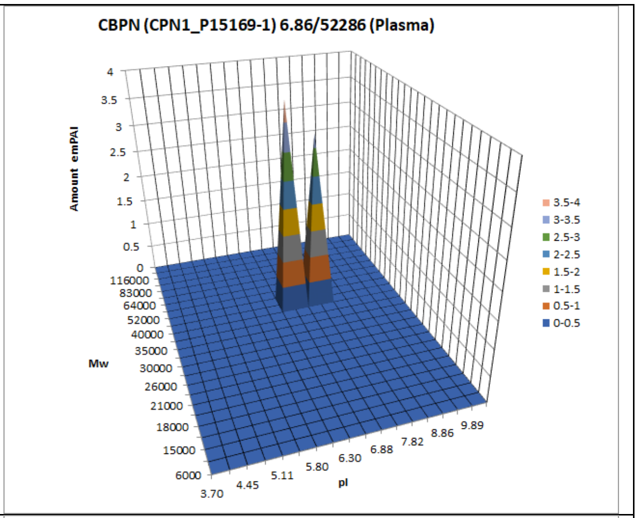
38.	CD14	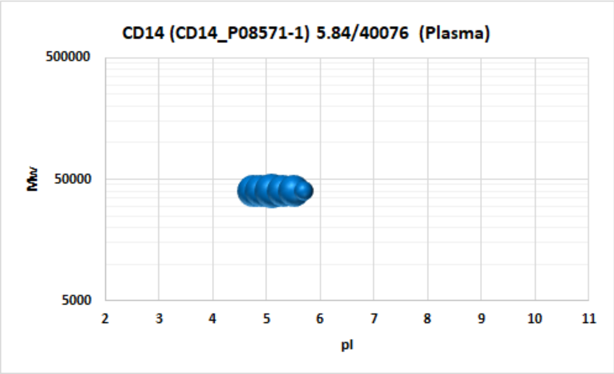	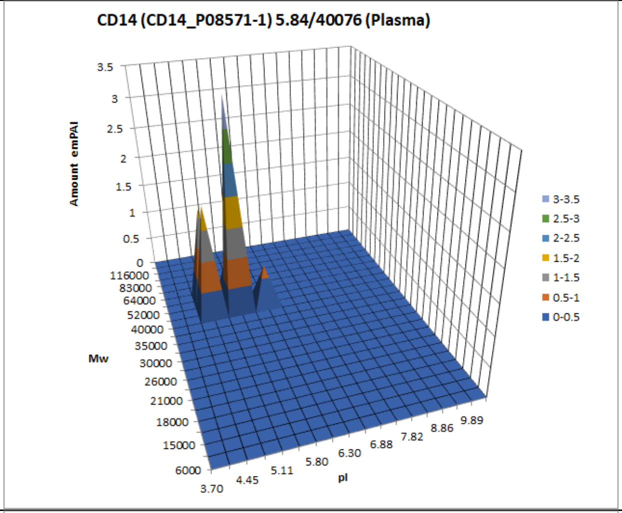
39.	CERU	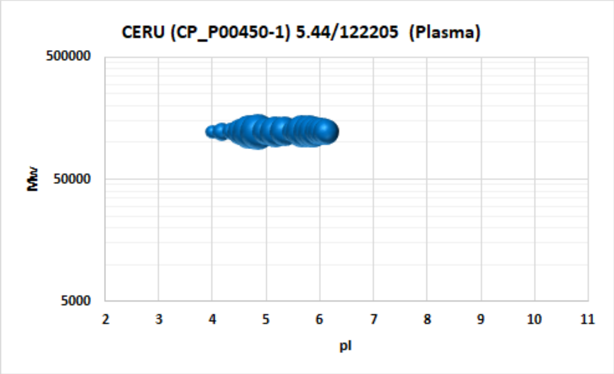	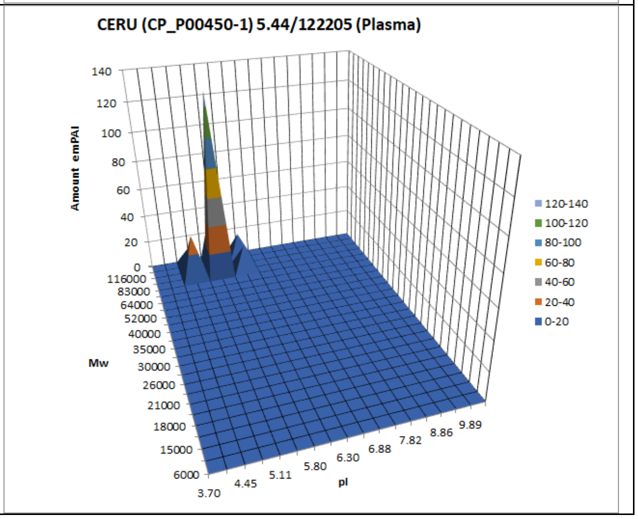

40.	CFAB	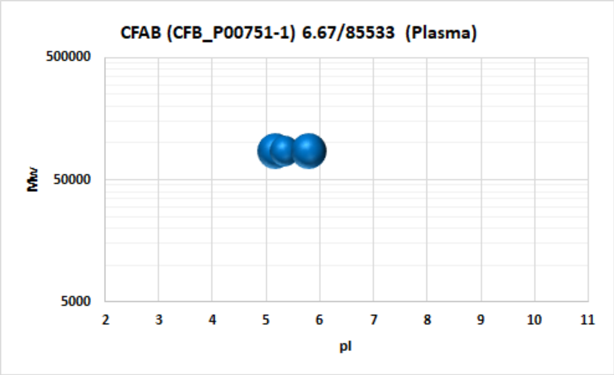	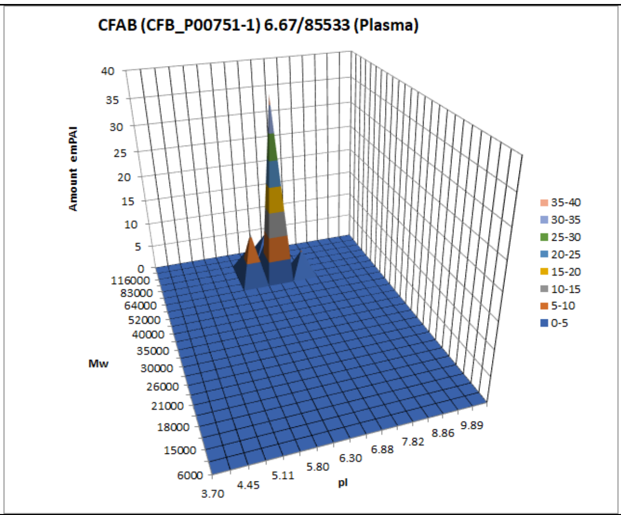
41.	CFAD	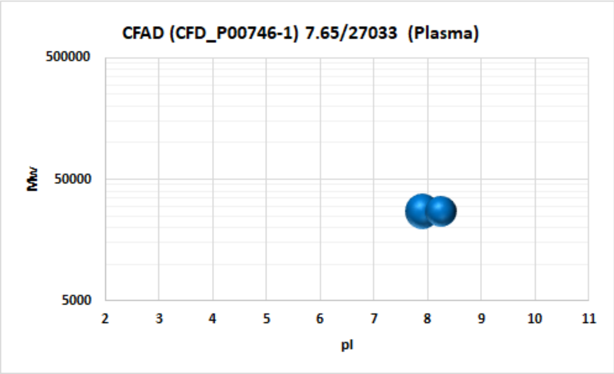	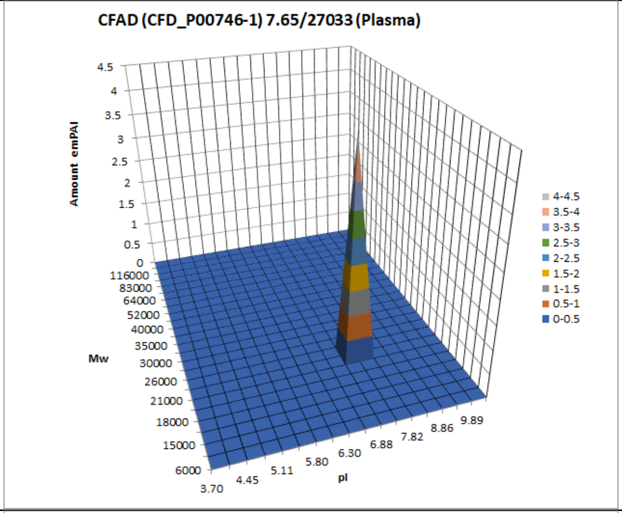
42.	CFAH	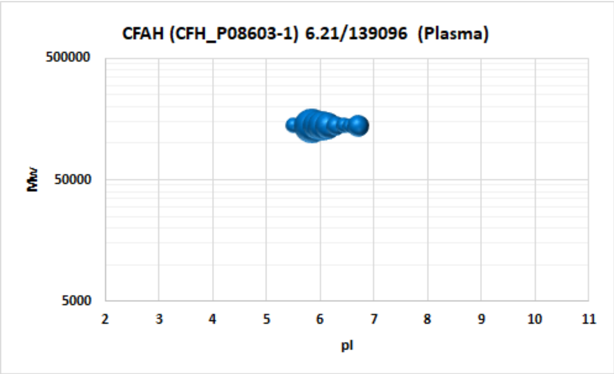	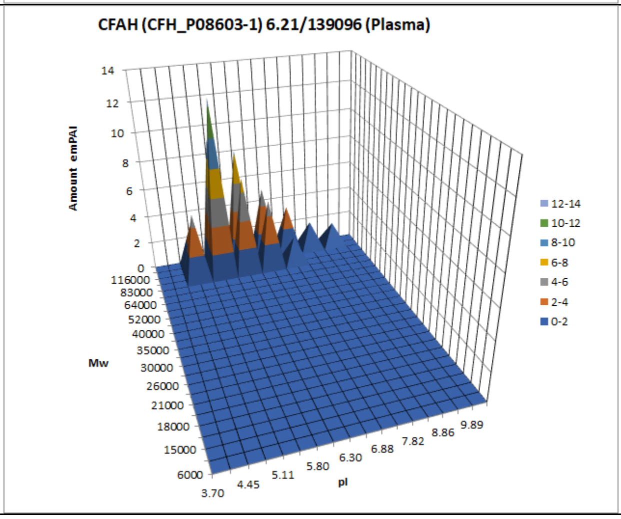

43.	CFAI	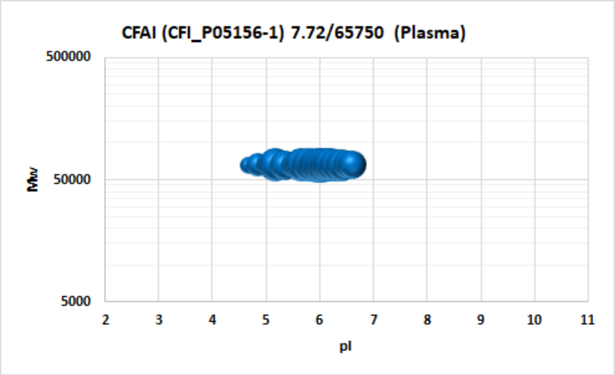	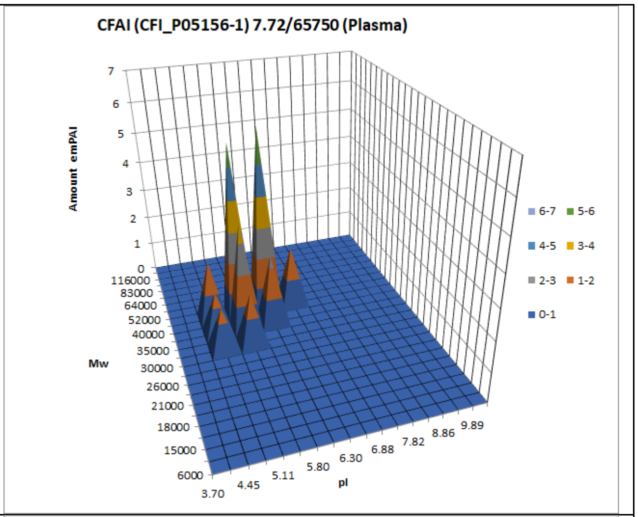
44.	CHLE	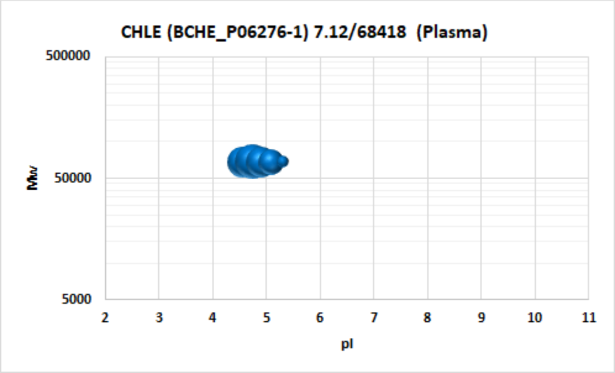	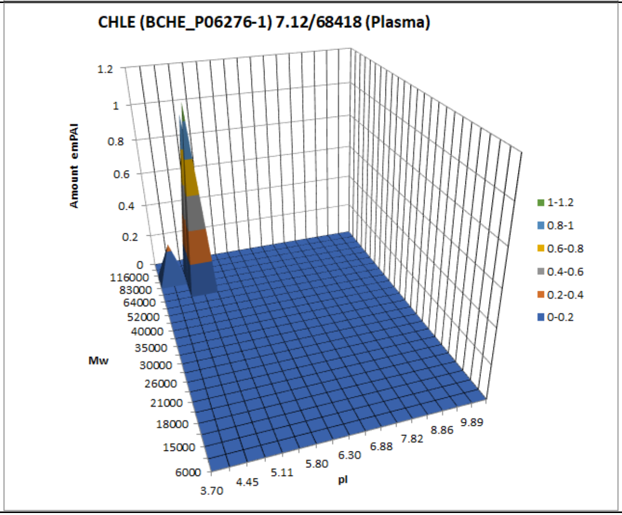
45.	CLUS	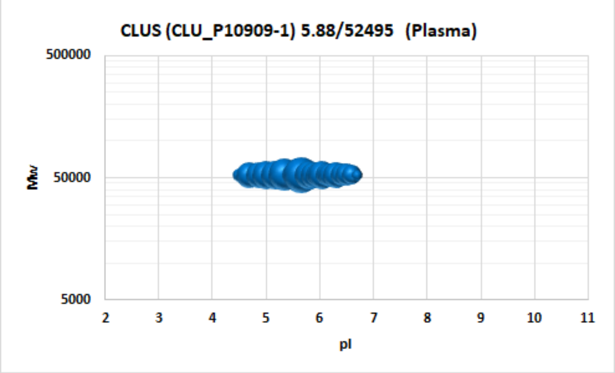	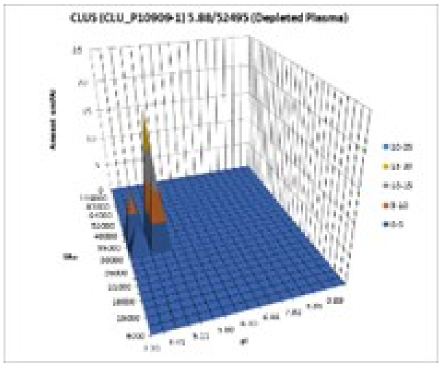

46.	CNDP1	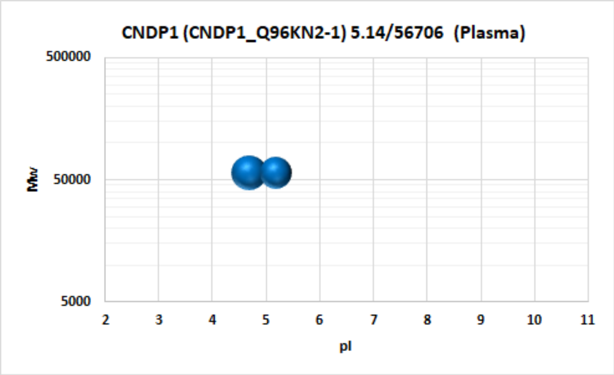	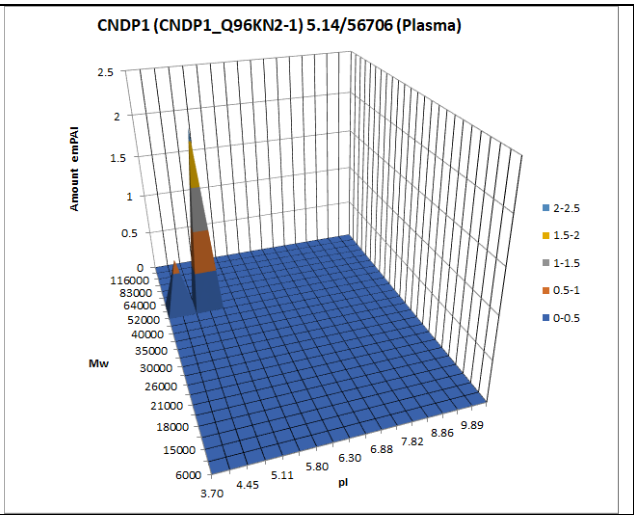
47.	CO2	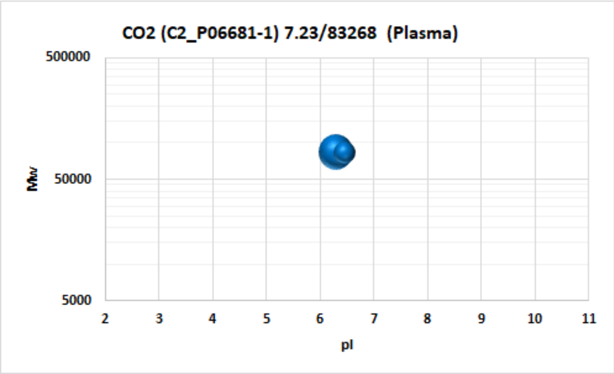	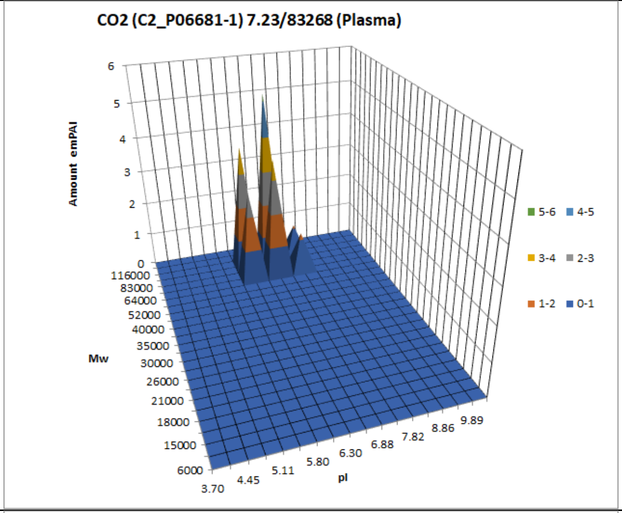
48.	CO3	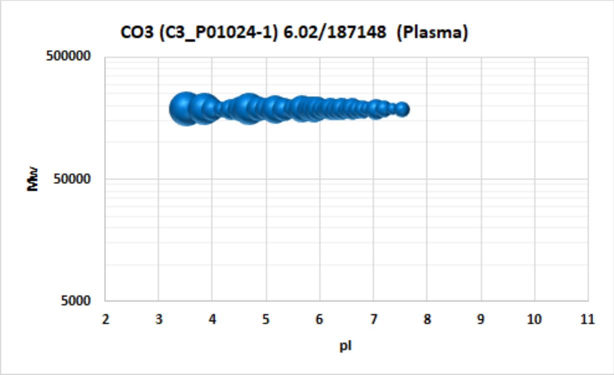	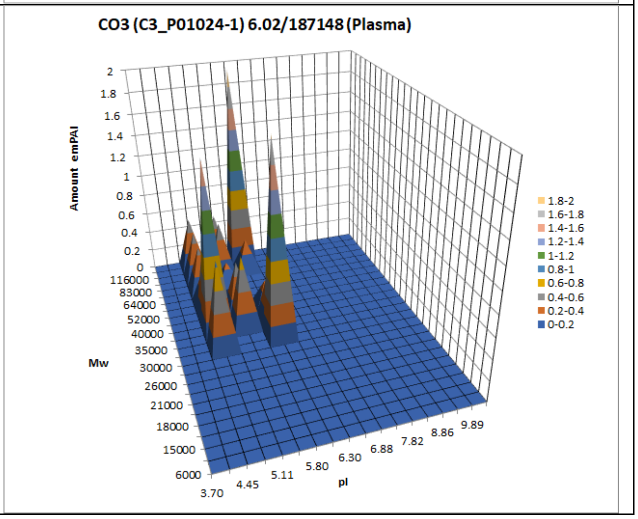

<table>
<tr>
<td>49.</td>
<td>CO4A</td>
<td>
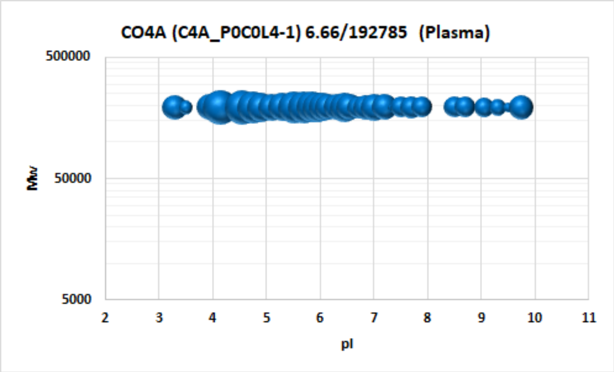

</td>
<td>
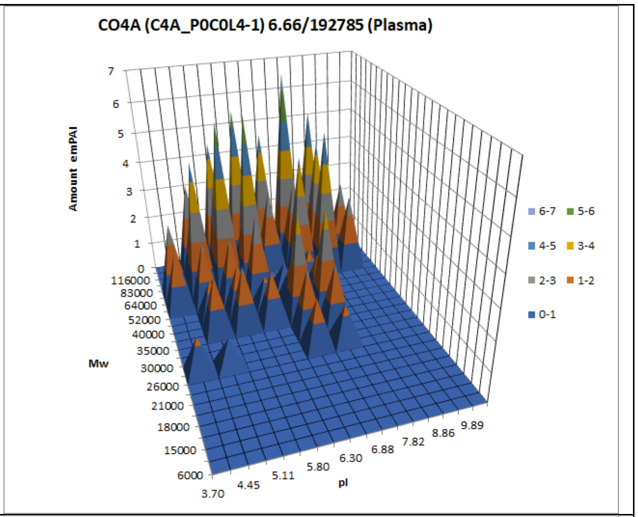

</td>
</tr>
<tr>
<td>50.</td>
<td>CO4B</td>
<td>
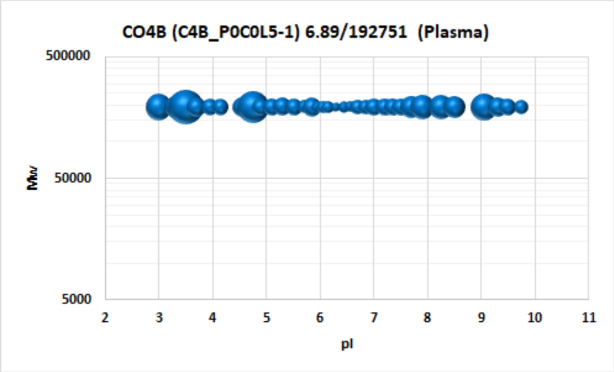

</td>
<td>
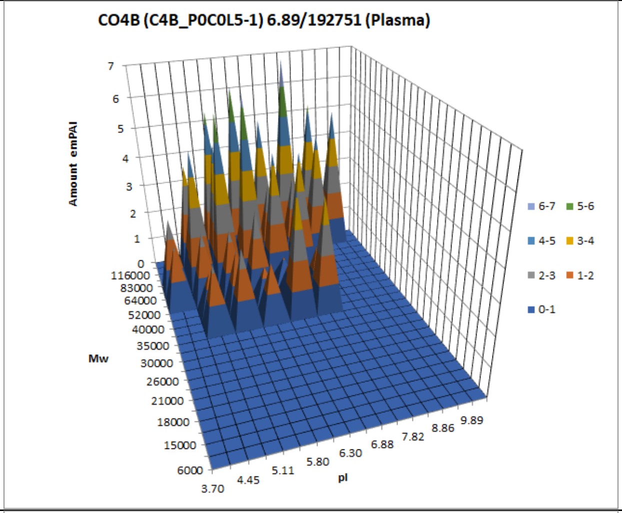

</td>
</tr>
<tr>
<td>51.</td>
<td>CO5</td>
<td>
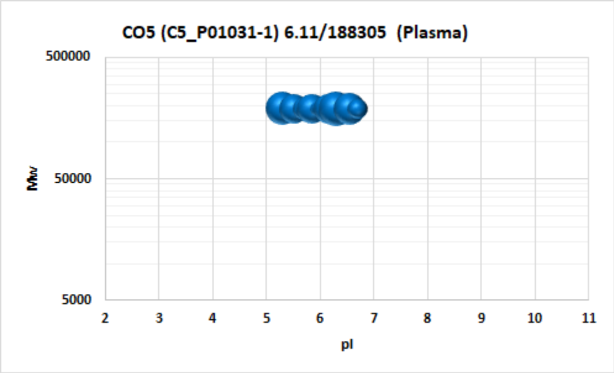

</td>
<td>
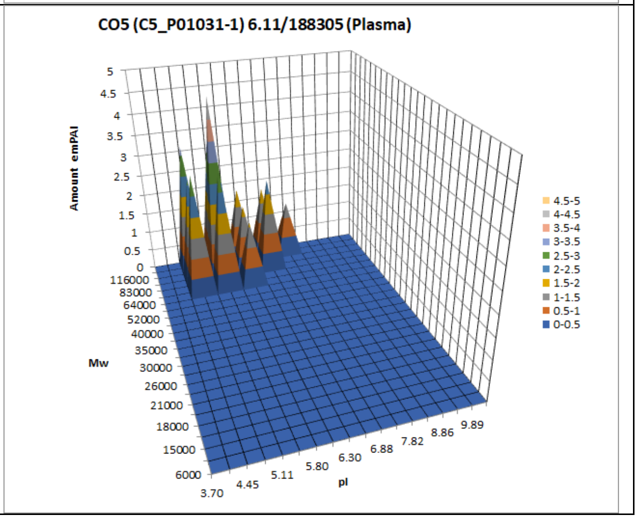

</td>
</tr>
</table>

52.	CO6	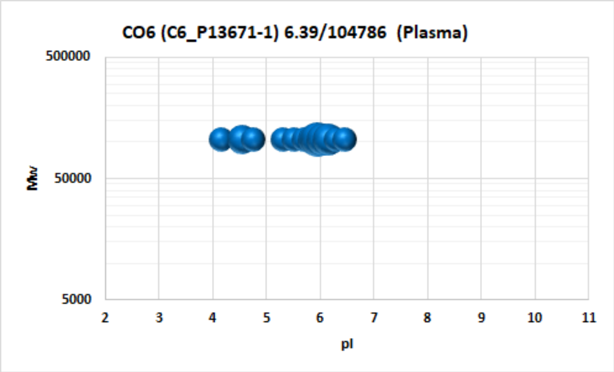	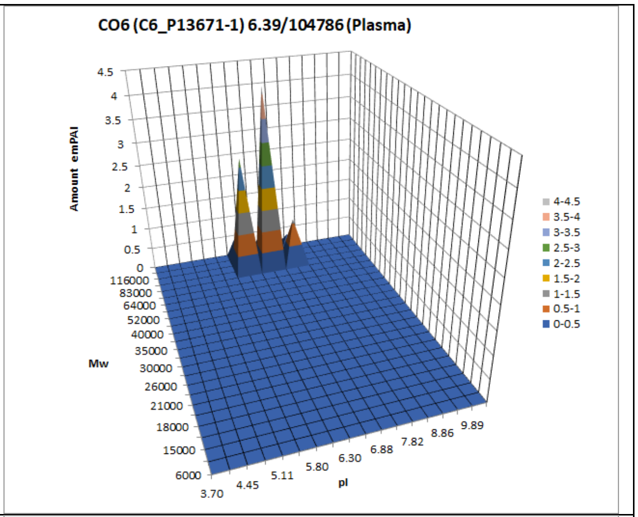
53.	CO7	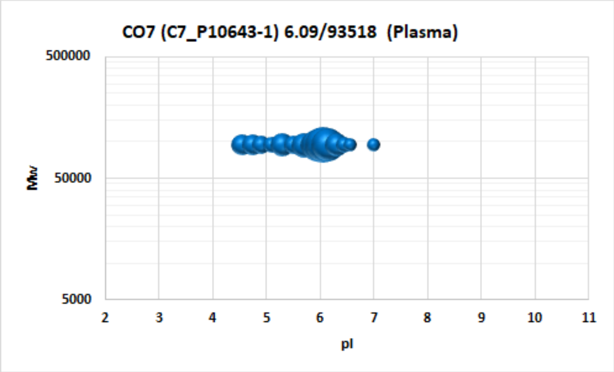	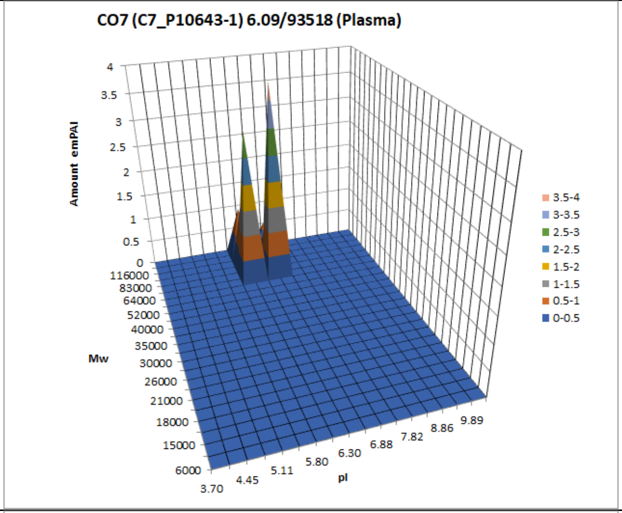
54.	CO8A	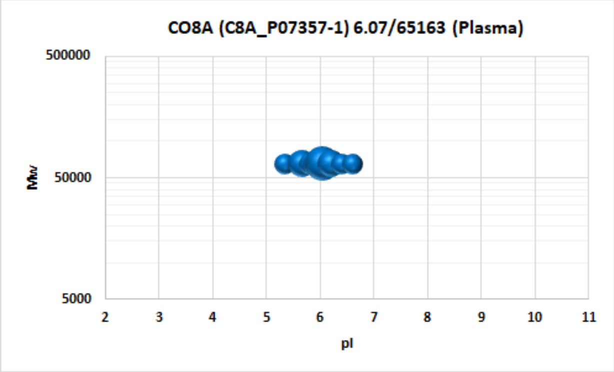	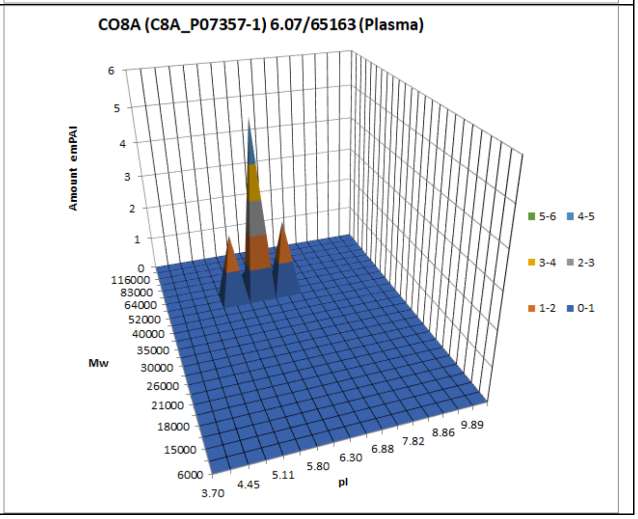

<table>
<tr>
<td>55.</td>
<td>CO8B</td>
<td>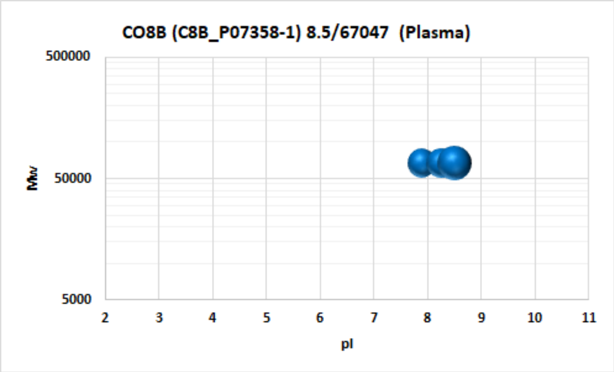
</td>
<td>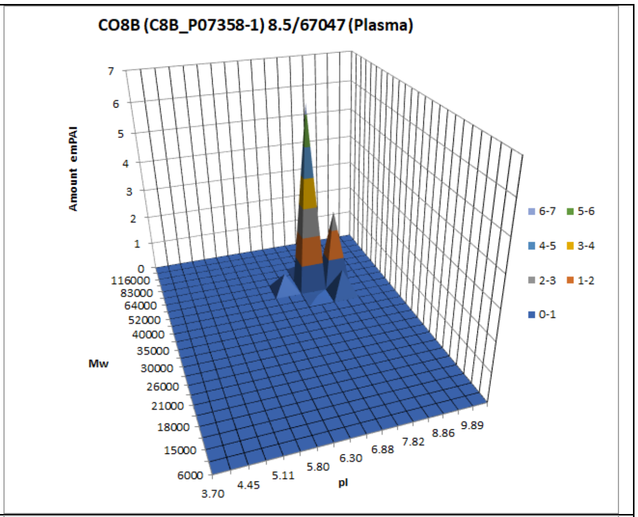
</td>
</tr>
<tr>
<td>56.</td>
<td>CO8G</td>
<td>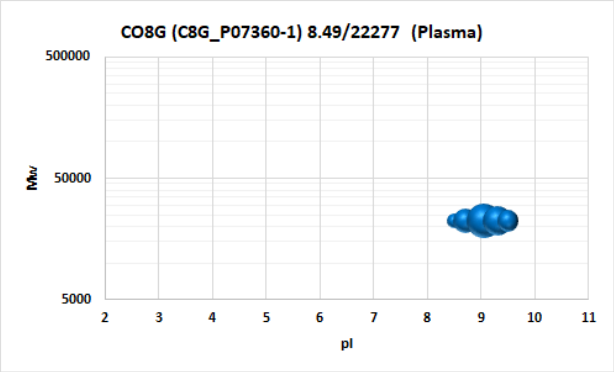
</td>
<td>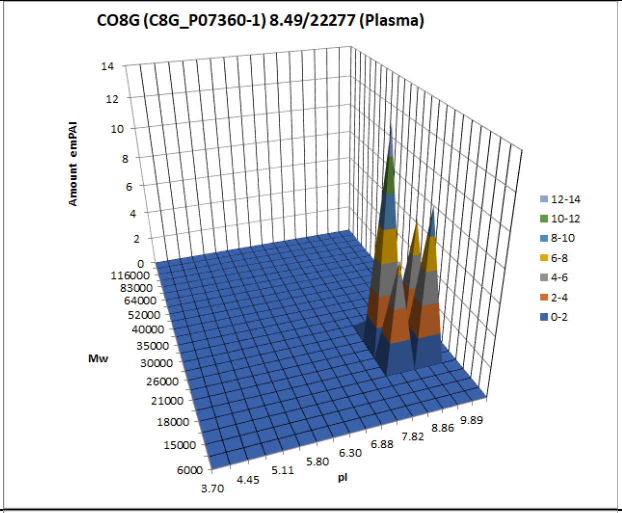
</td>
</tr>
<tr>
<td>57.</td>
<td>CO9</td>
<td>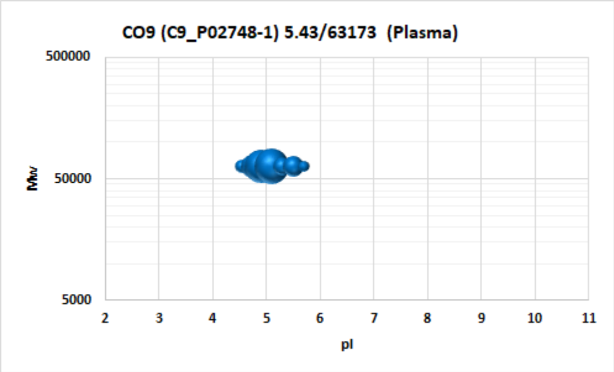
</td>
<td>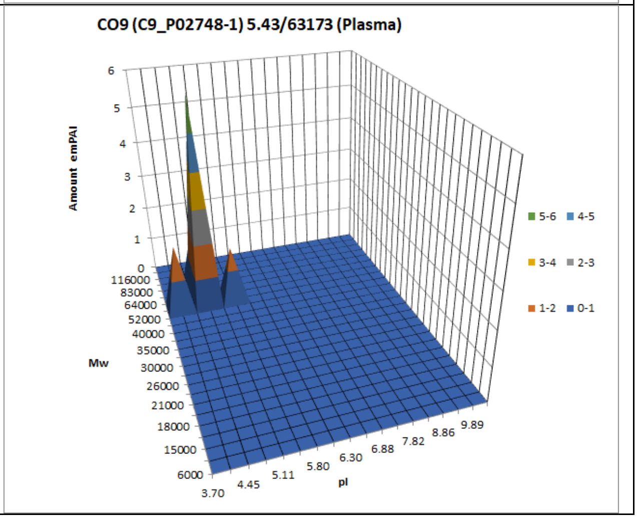
</td>
</tr>
</table>

<table>
<tr><td>58.</td><td>CPN2</td><td>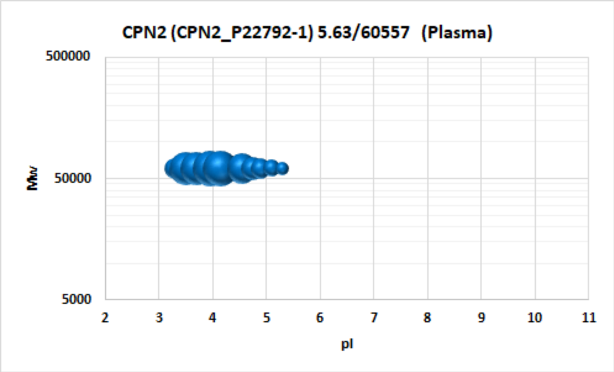
</td><td>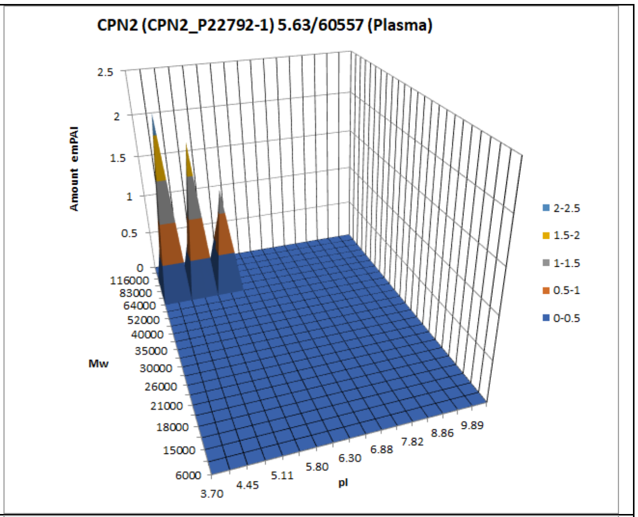
</td></tr>
<tr><td>59.</td><td>CRP</td><td>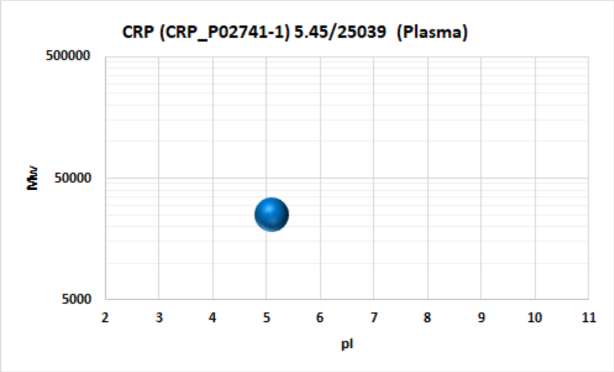
</td><td>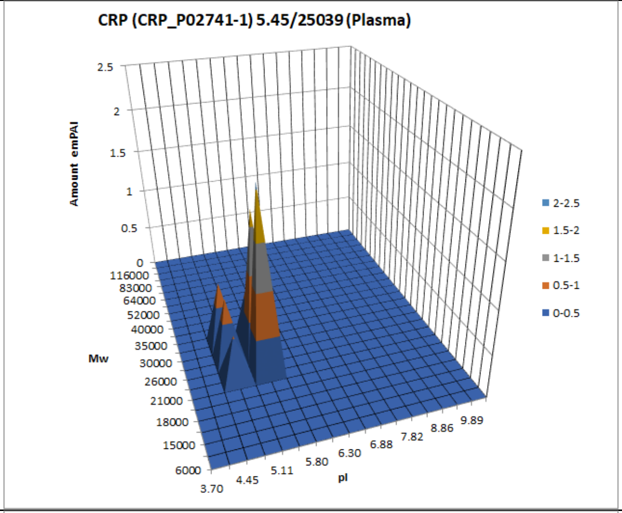
</td></tr>
<tr><td>60.</td><td>ECM1</td><td>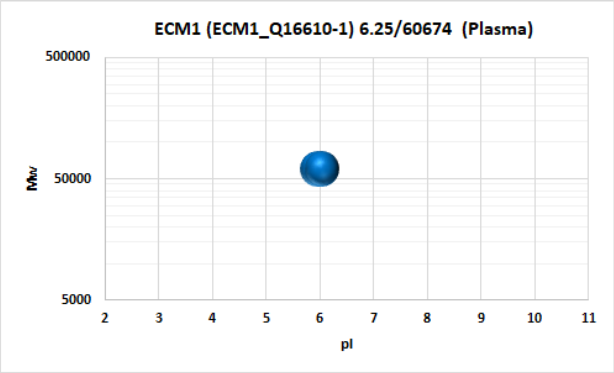
</td><td>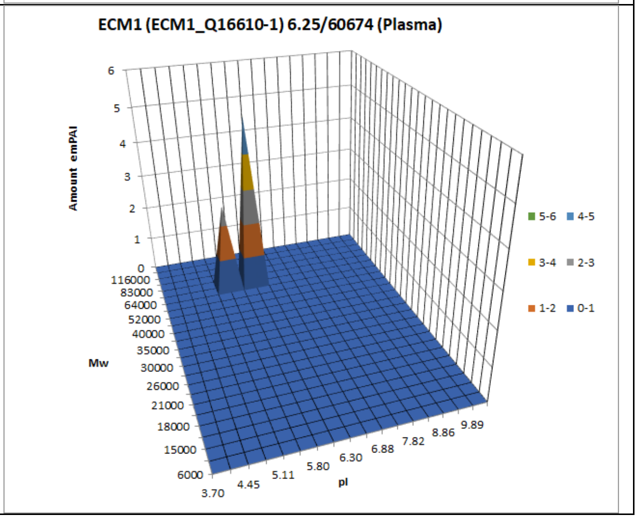
</td></tr>
</table>

61.	F13B	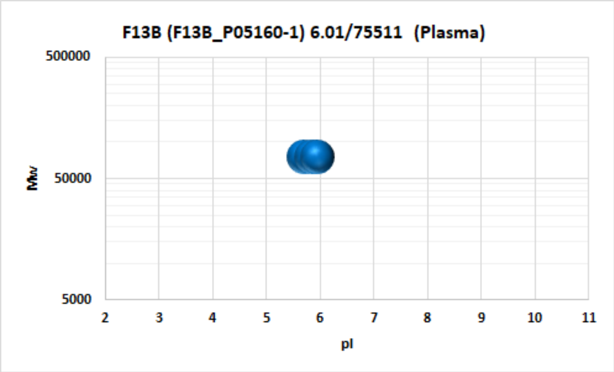	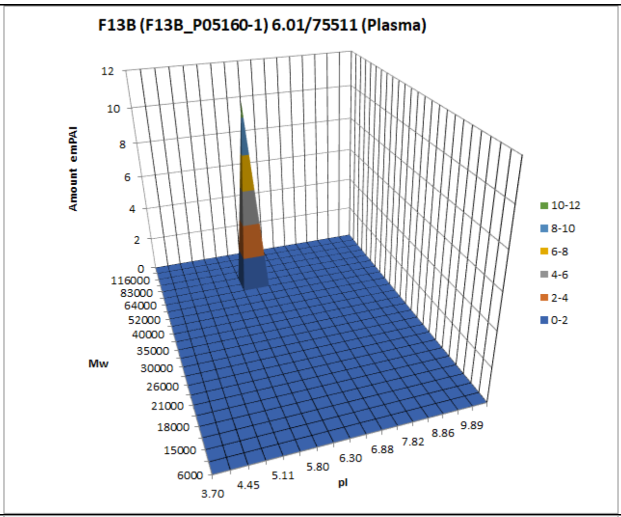
62.	FBLN1	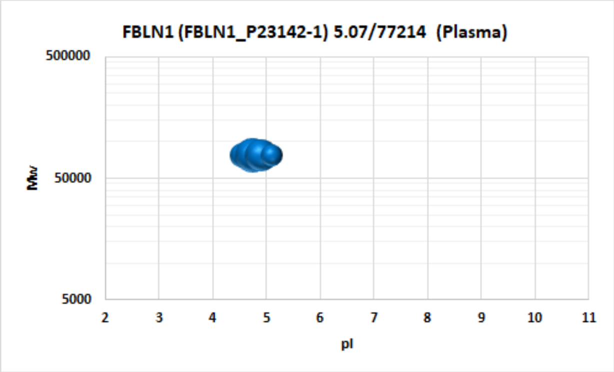	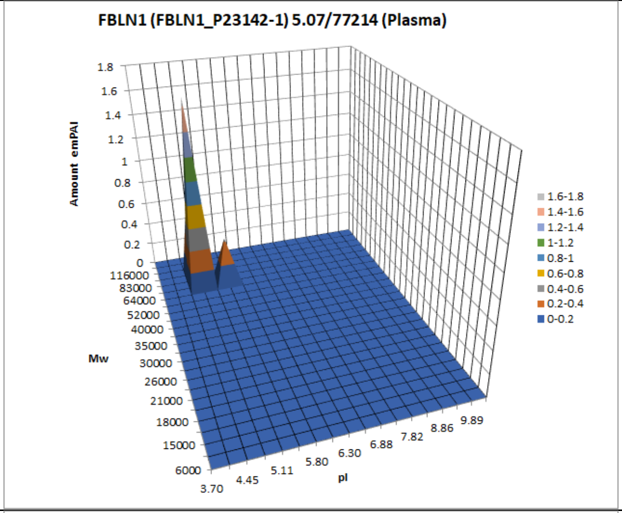
63.	FCN3	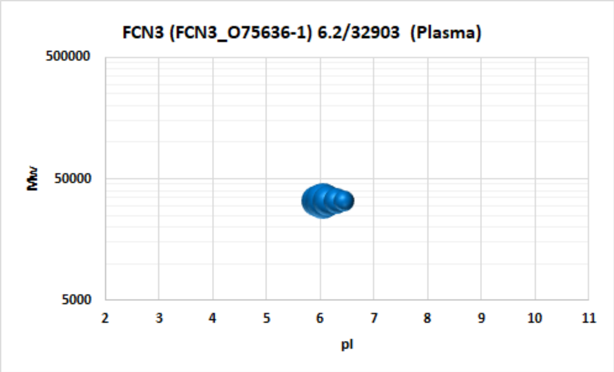	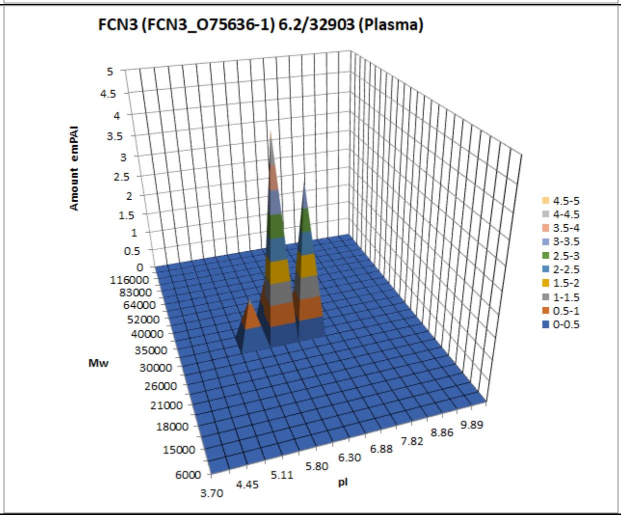

64.	FETUA	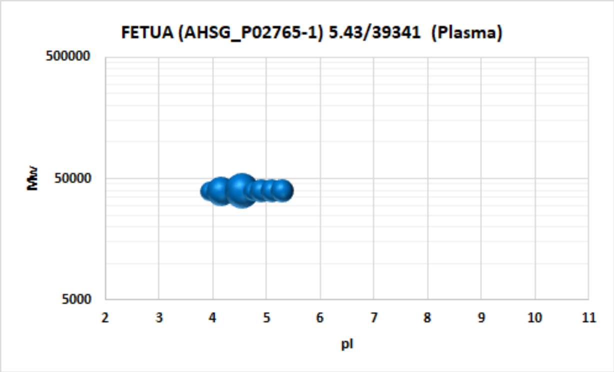	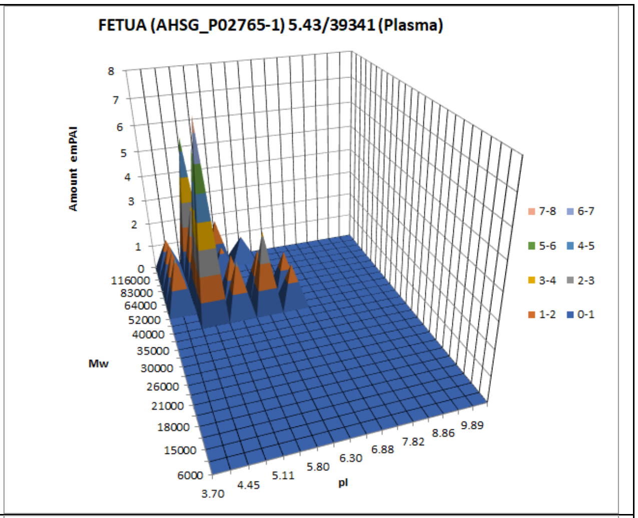
65.	FETUB	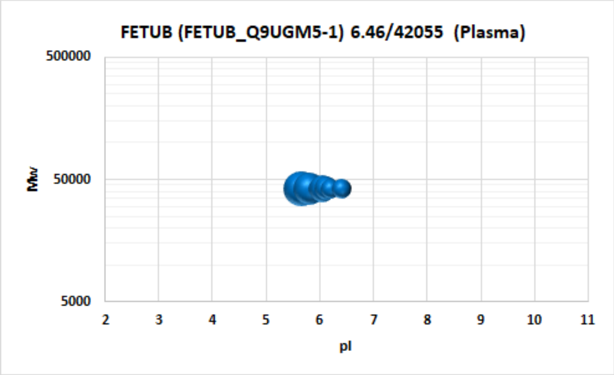	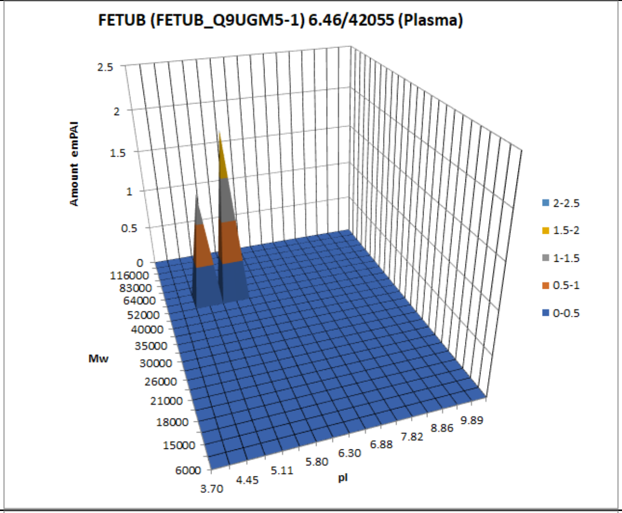
66.	FIBA	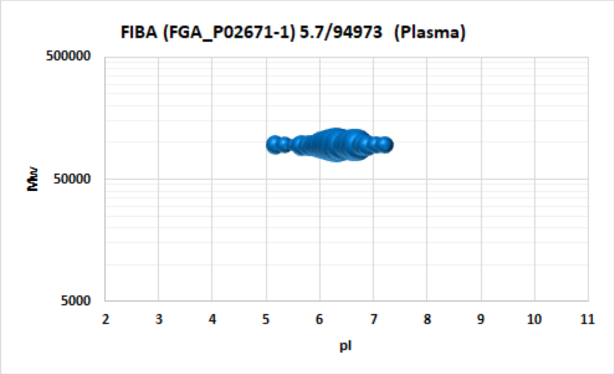	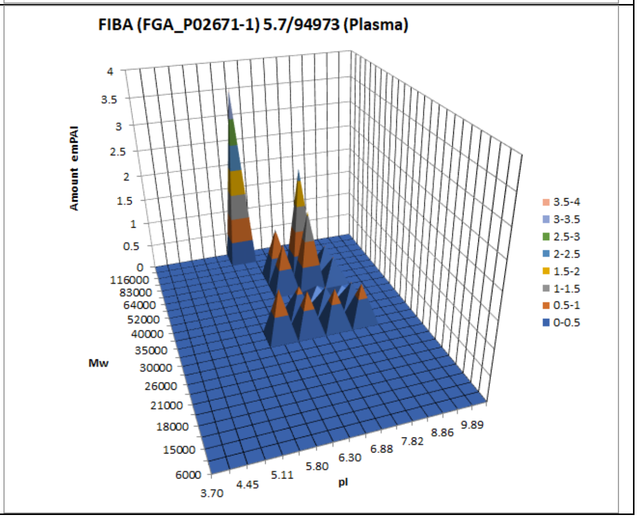

67.	FIBB	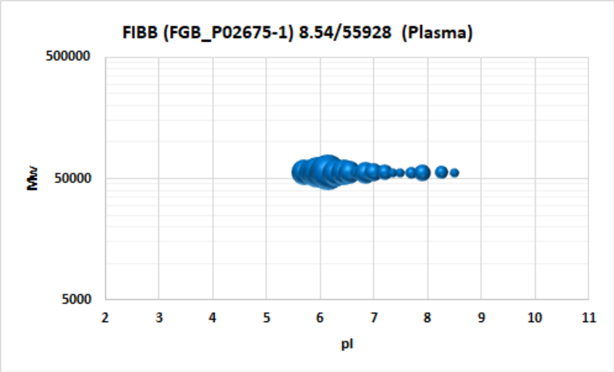	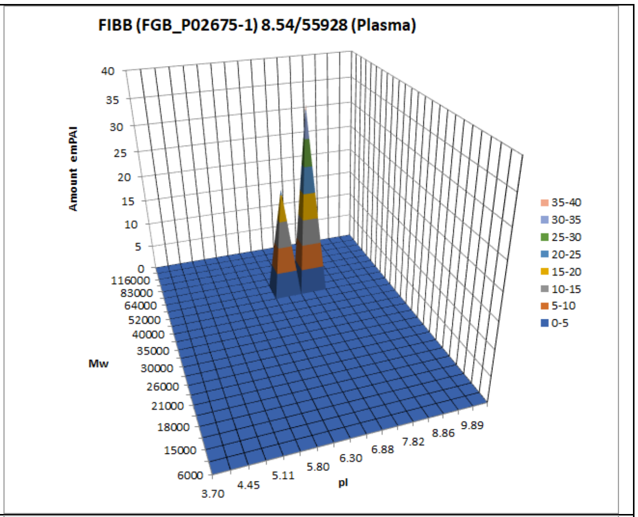
68.	FIBG	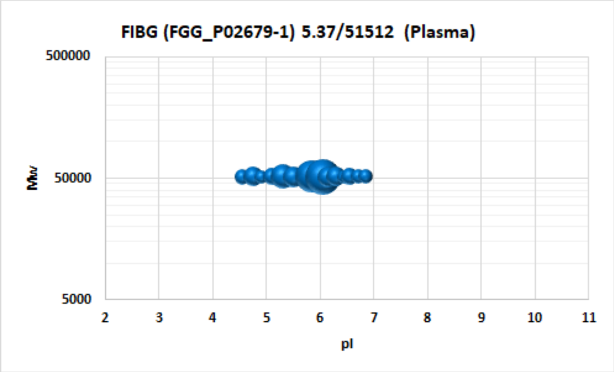	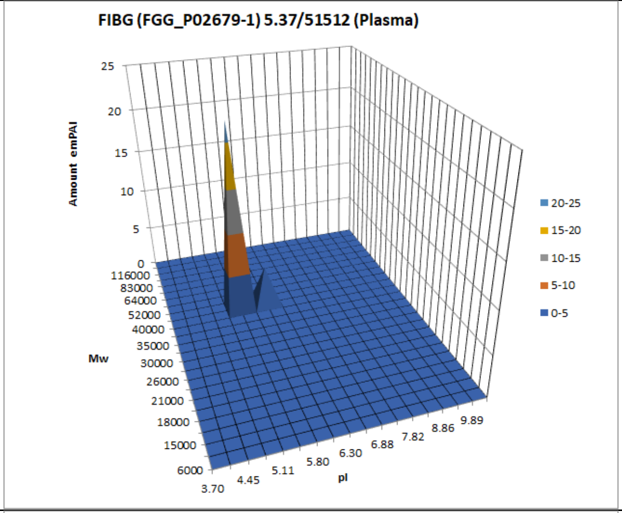
69.	FINC	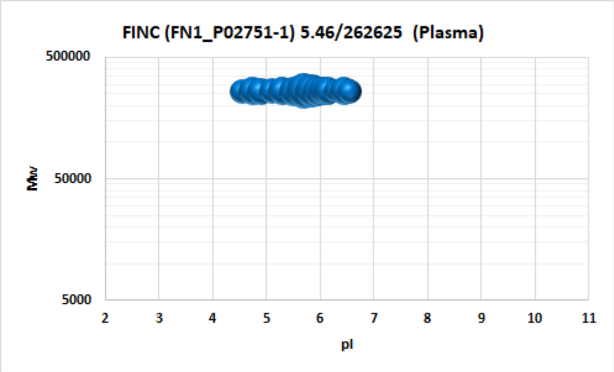	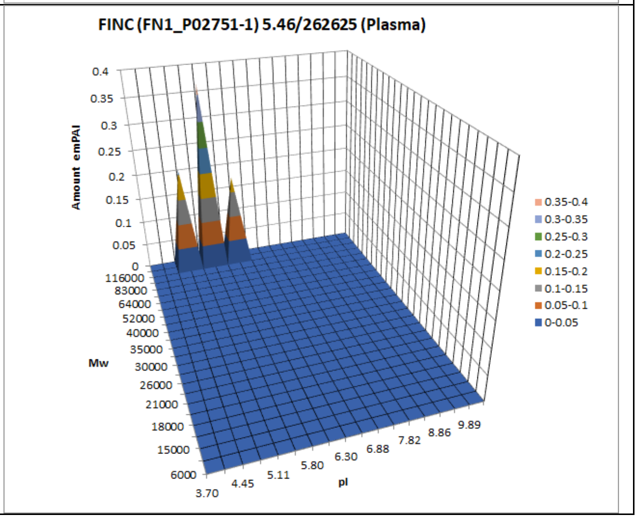

70.	GELS	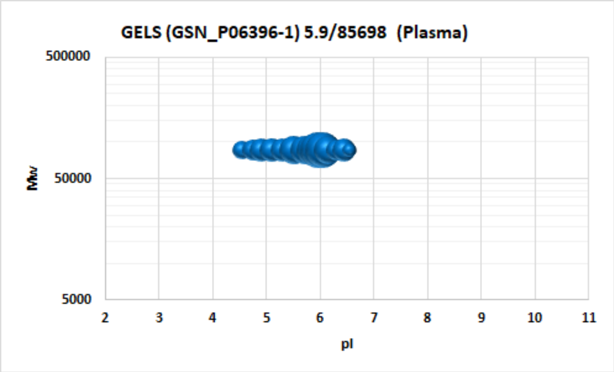	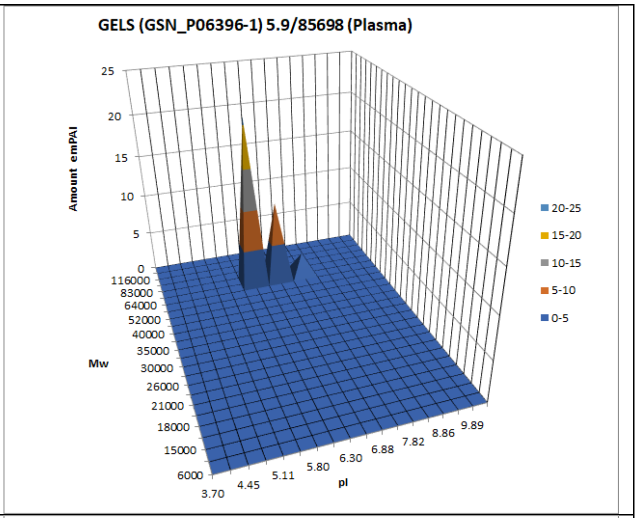
71.	GPX3	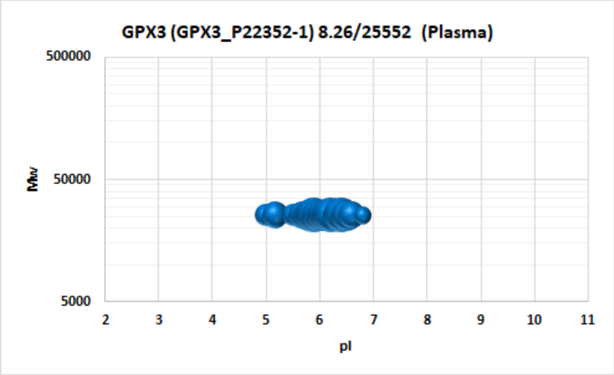	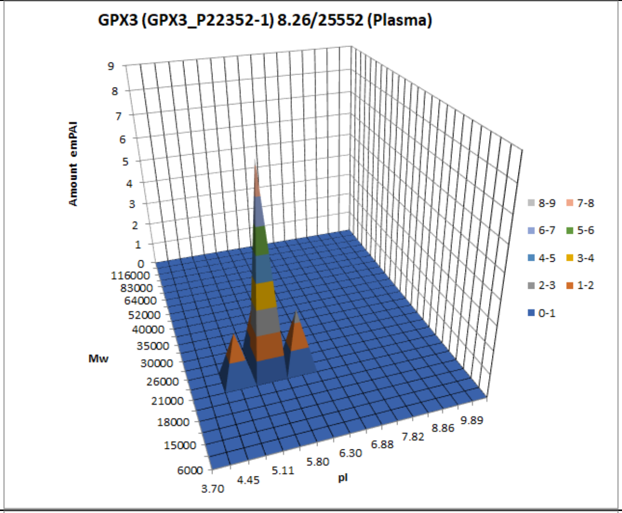
72.	HBA	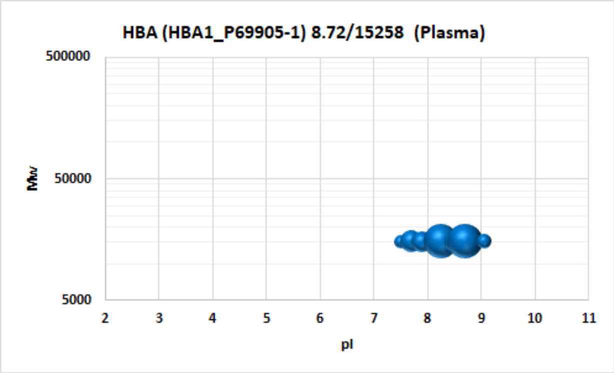	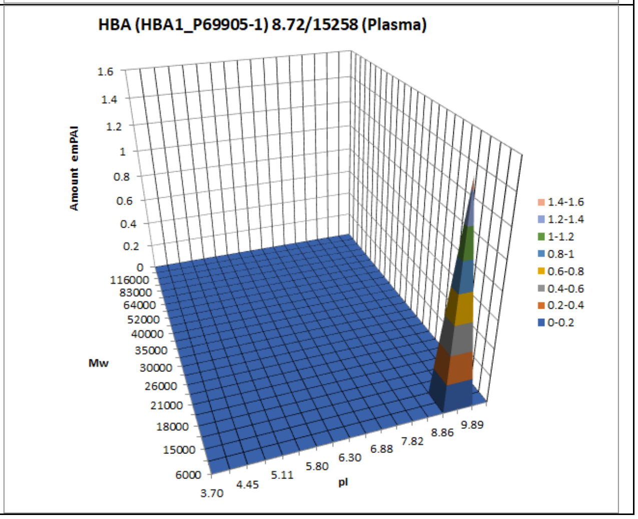

73.	HBB	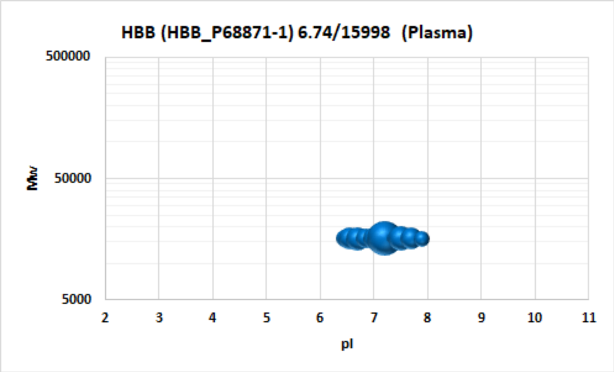	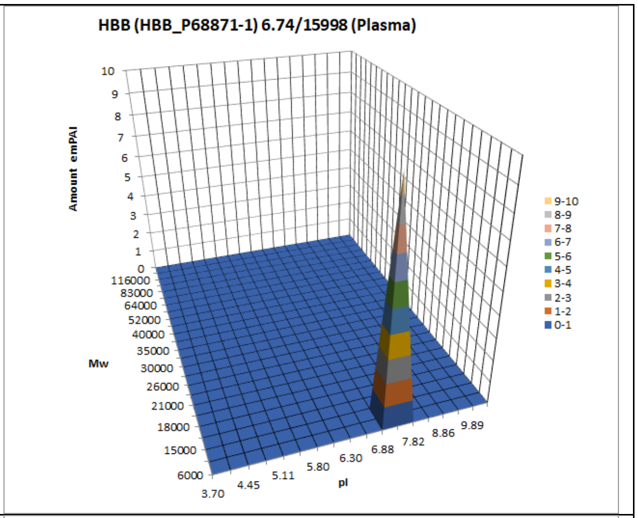
74.	HBD	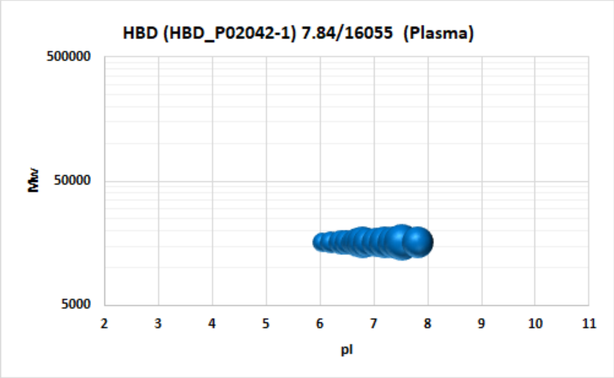	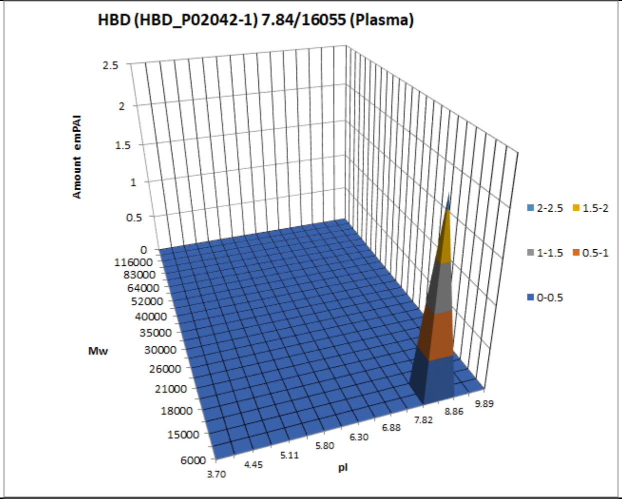
75.	HEMO	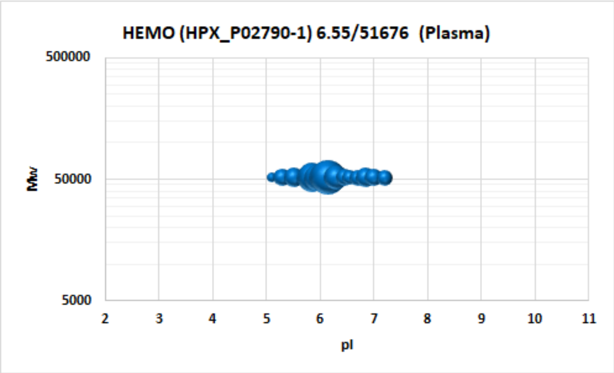	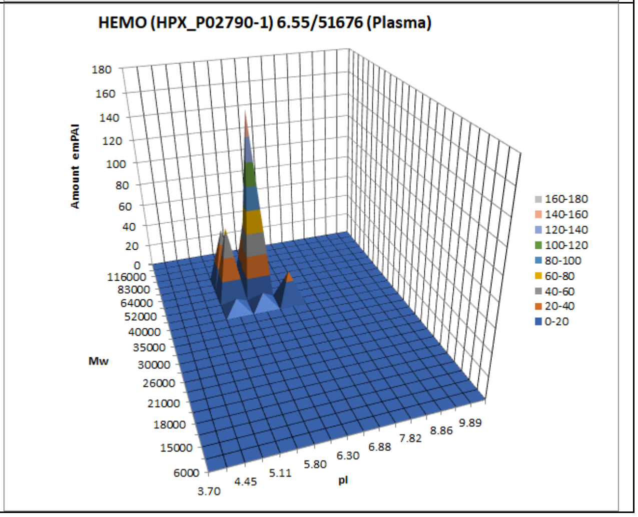

76.	HEP2	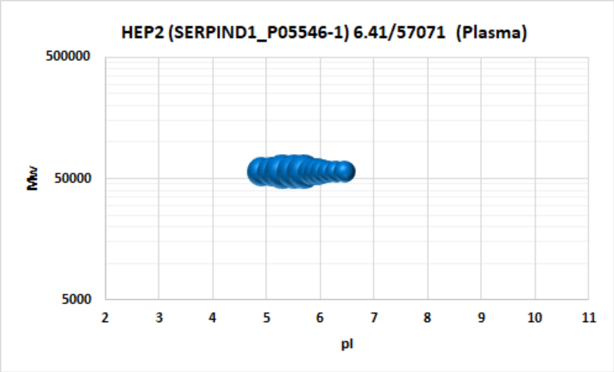	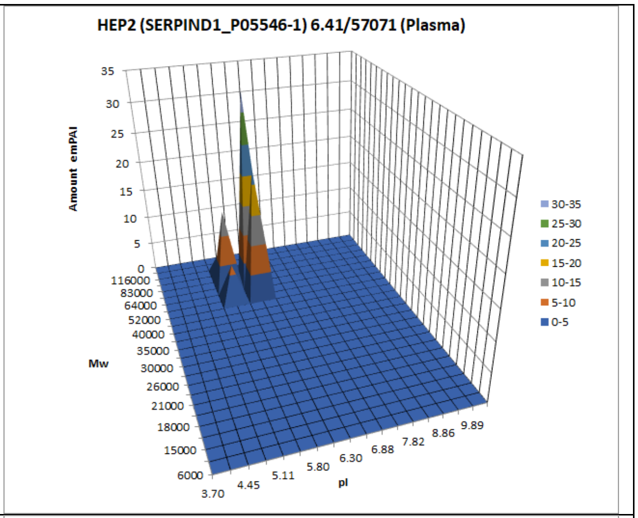
77.	HPT	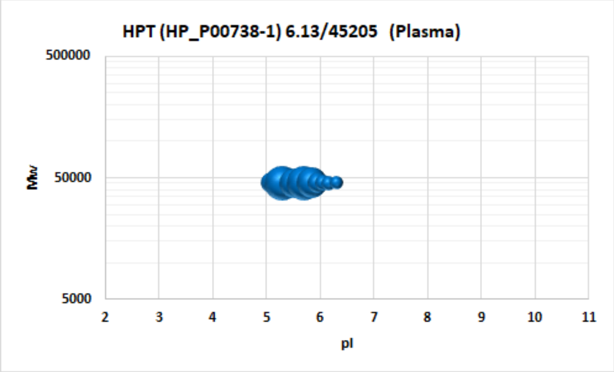	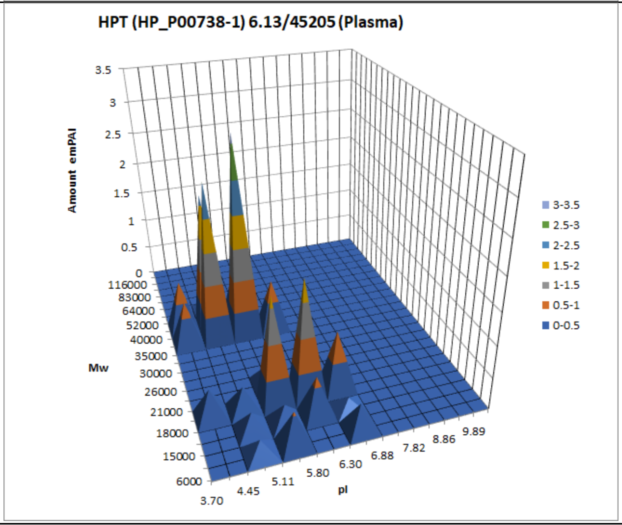
78.	HPT-alpha<br>HPT-beta	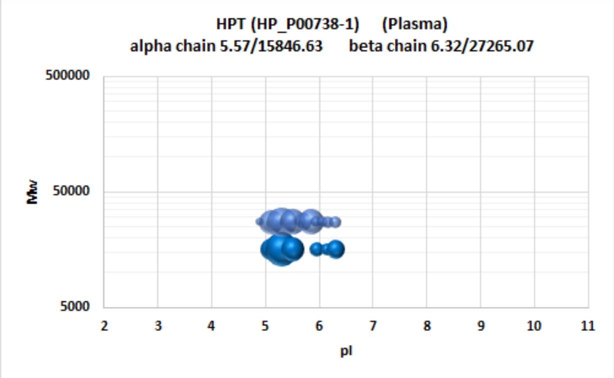	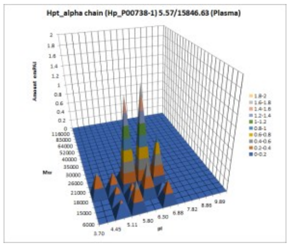 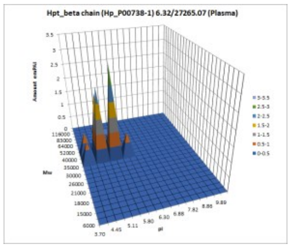

79.	HPTR	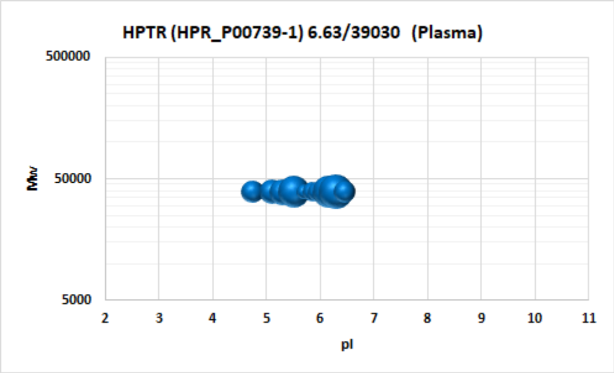	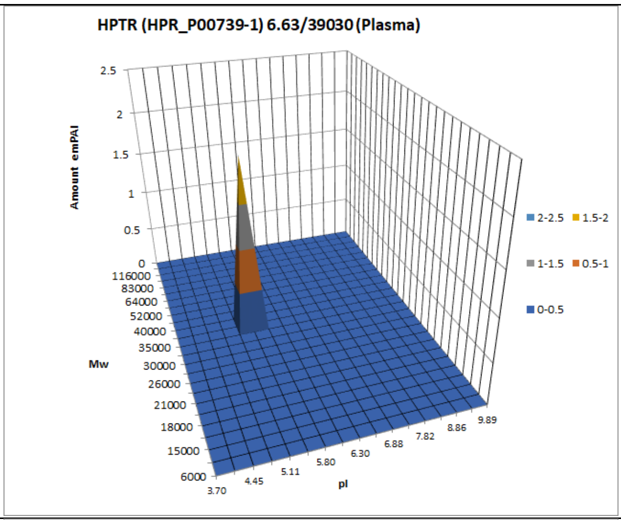
80.	HRG	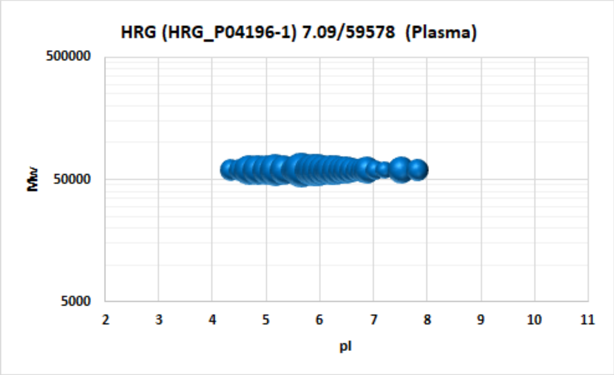	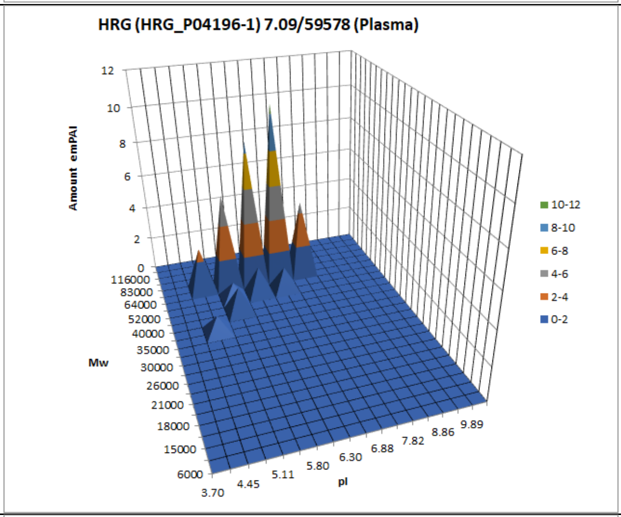
81.	IC1	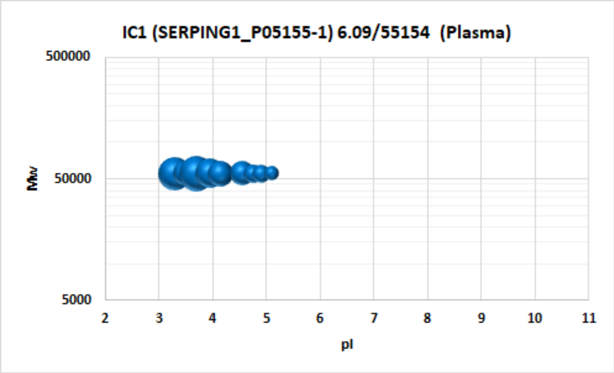	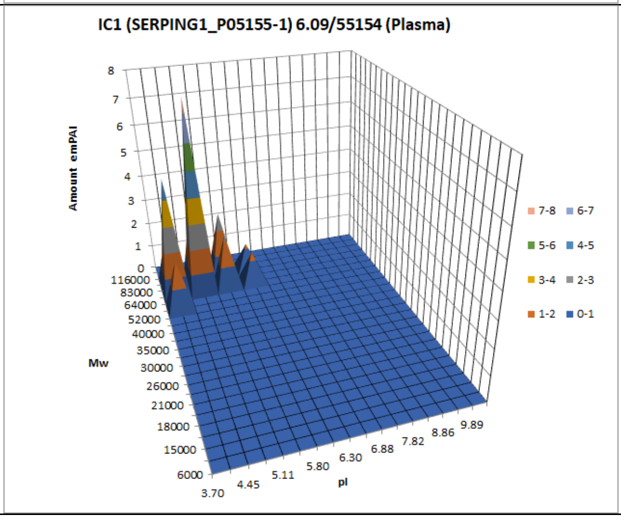

<table>
<tr><td>82.</td><td>ITIH1</td><td>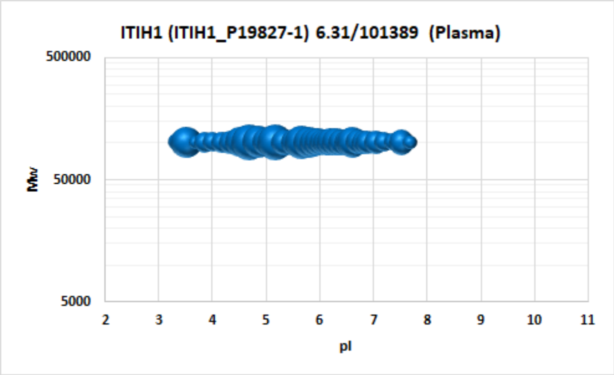
</td><td>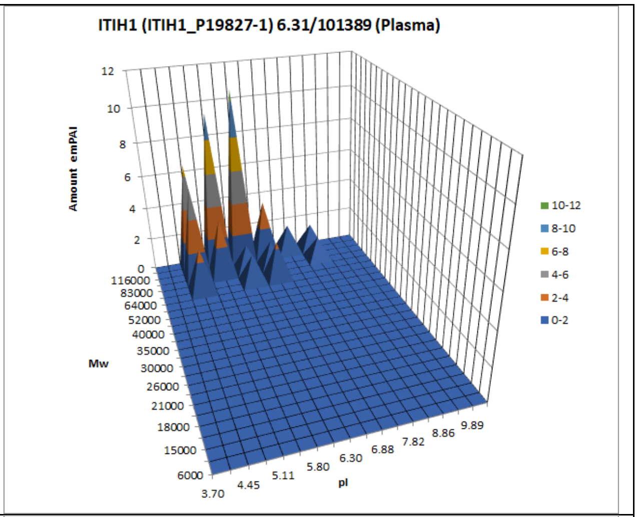
</td></tr>
<tr><td>83.</td><td>ITIH2</td><td>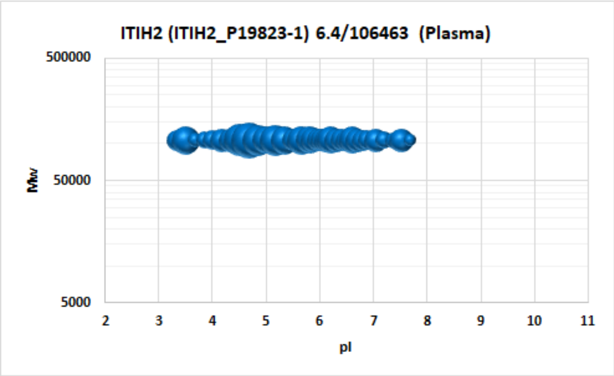
</td><td>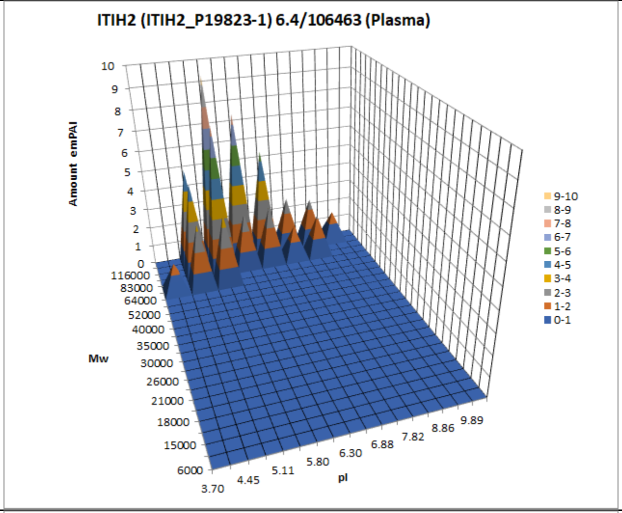
</td></tr>
<tr><td>84.</td><td>ITIH3</td><td>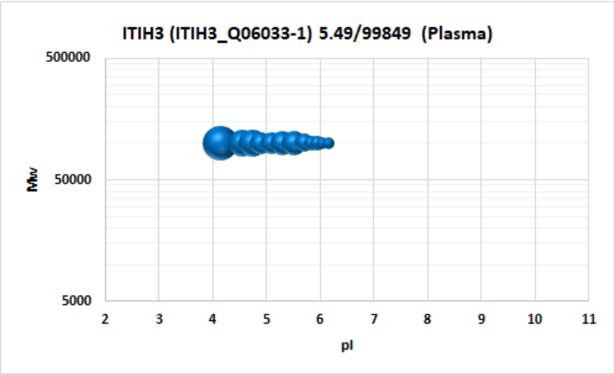
</td><td>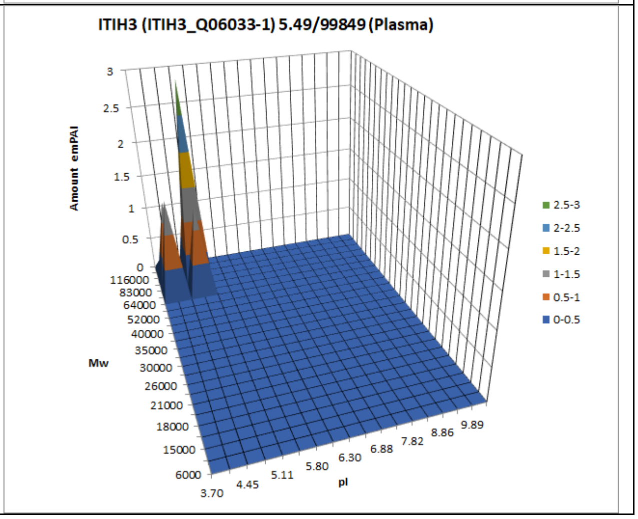
</td></tr>
</table>

<table>
<tr><td>85.</td><td>ITIH4</td><td>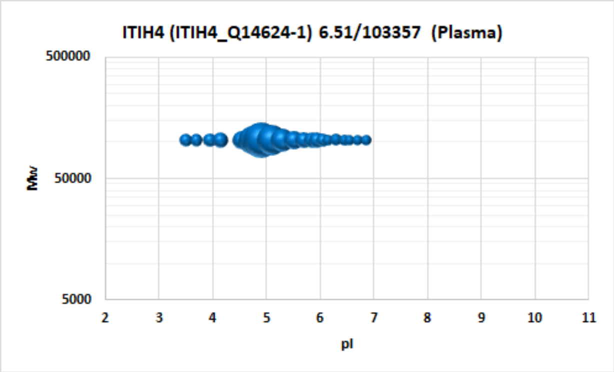
</td><td>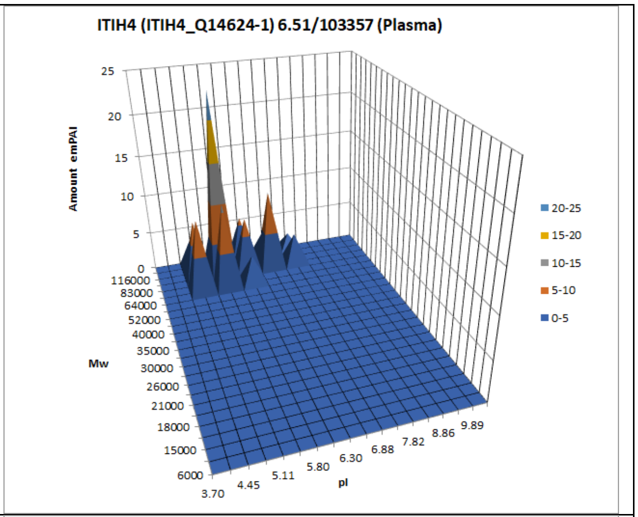
</td></tr>
<tr><td>86.</td><td>KAIN</td><td>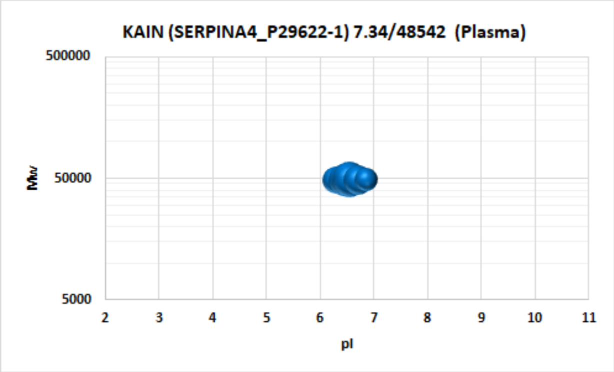
</td><td>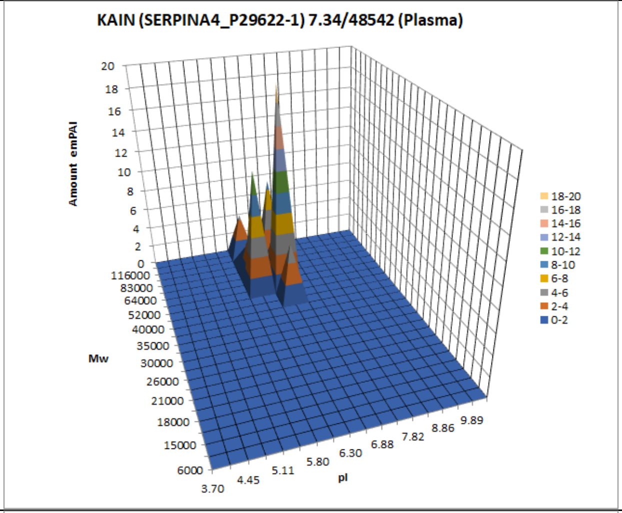
</td></tr>
<tr><td>87.</td><td>KNG1</td><td>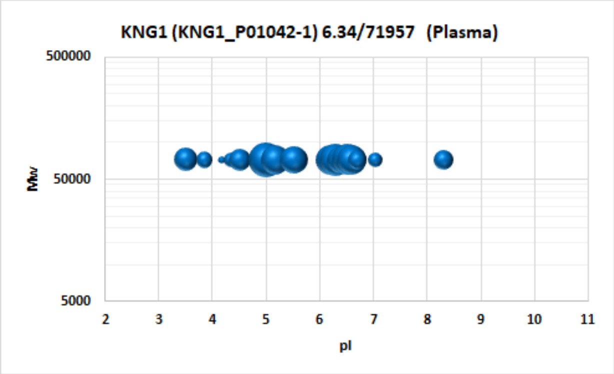
</td><td>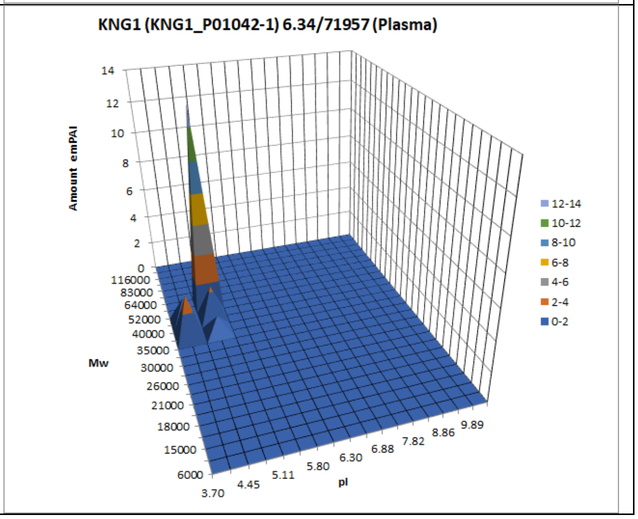
</td></tr>
</table>

88.	LCAT	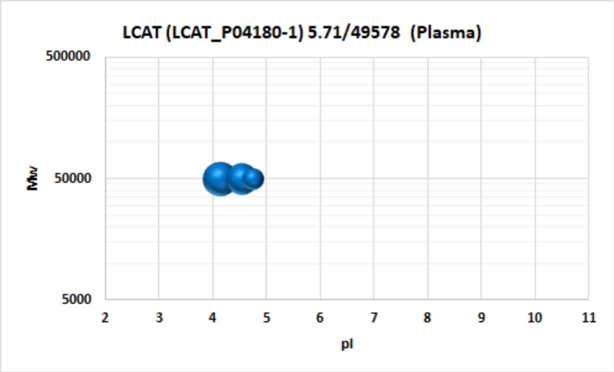	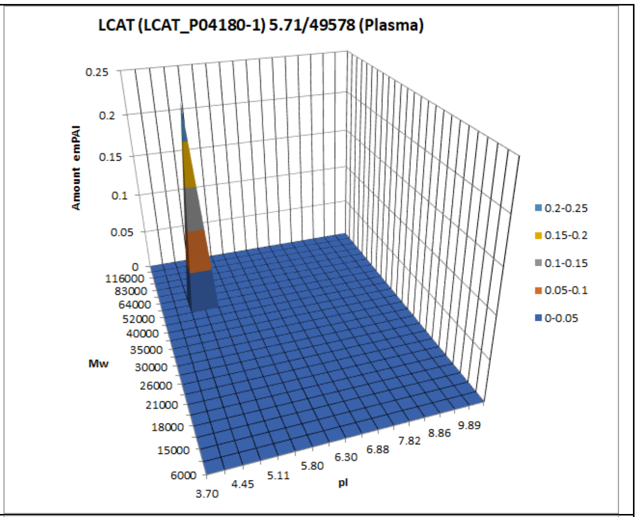
89.	LUM	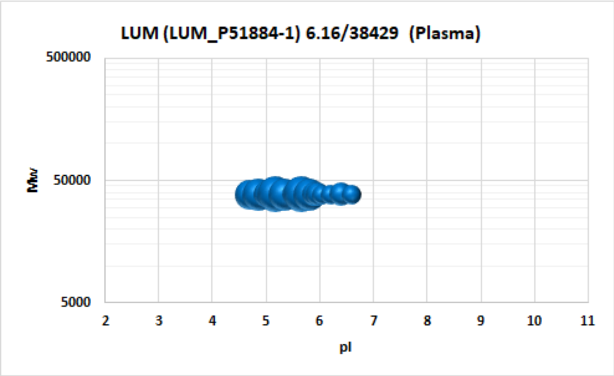	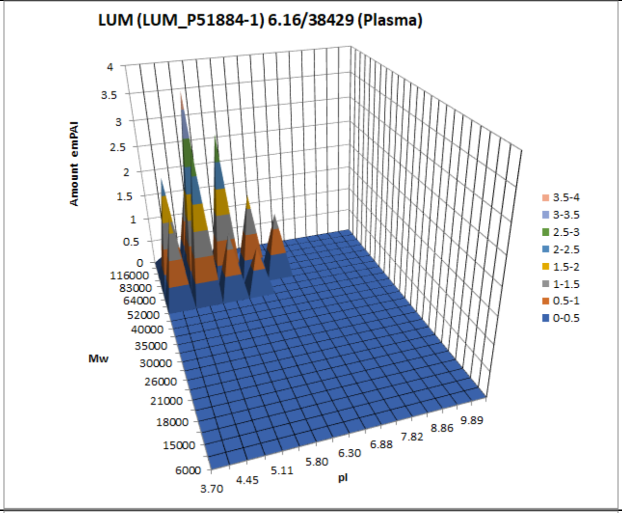
90.	MBL2	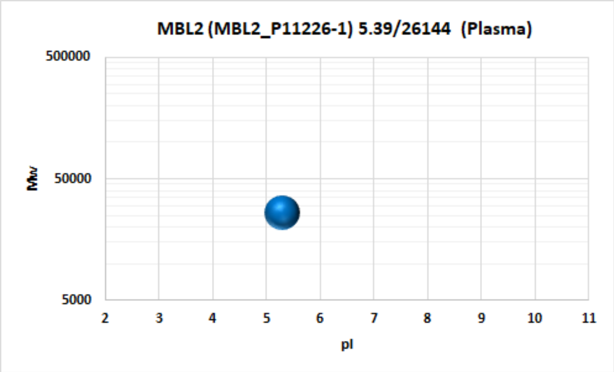	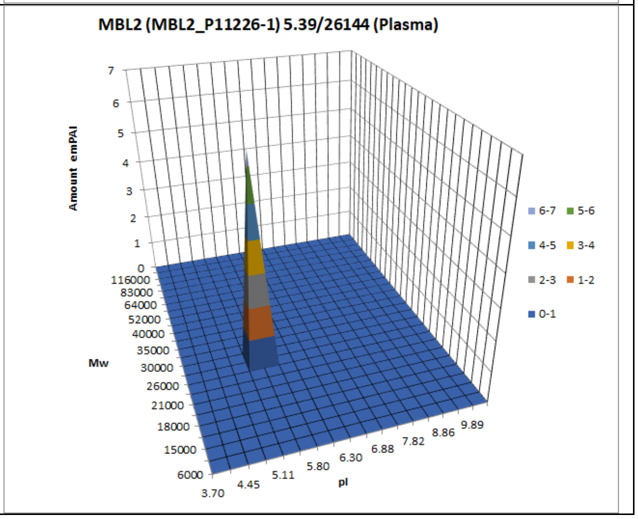

<table>
<tr><td>91.</td><td>PEDF</td><td>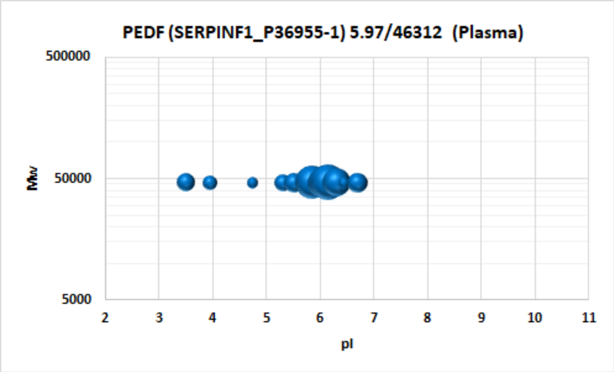
</td><td>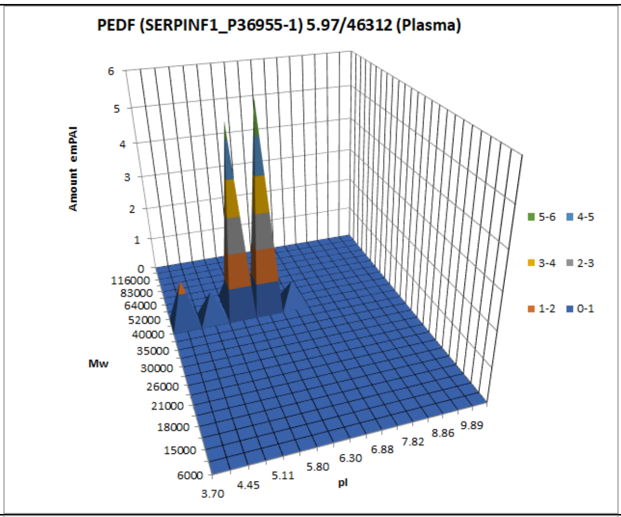
</td></tr>
<tr><td>92.</td><td>PGRP2</td><td>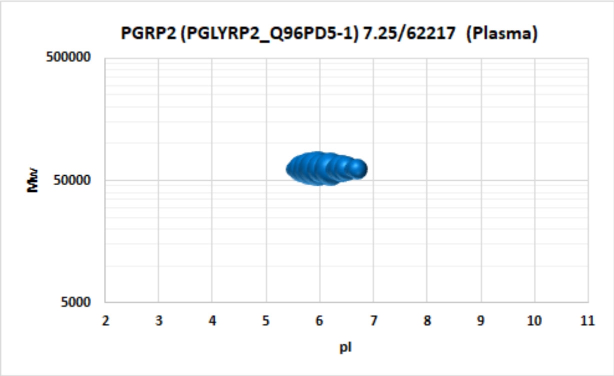
</td><td>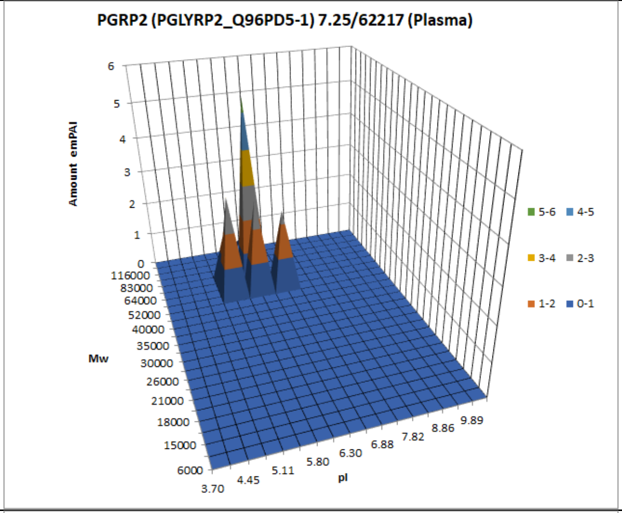
</td></tr>
<tr><td>93.</td><td>PHLD</td><td>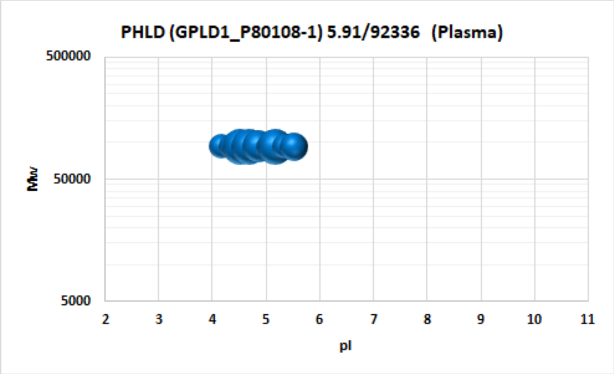
</td><td>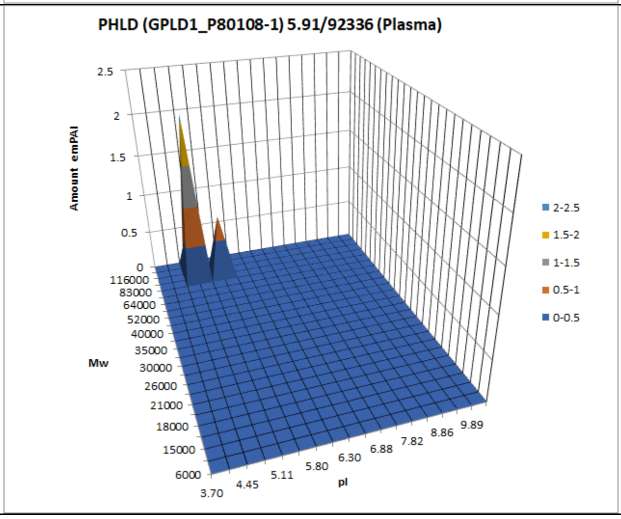
</td></tr>
</table>

94.	PLMN	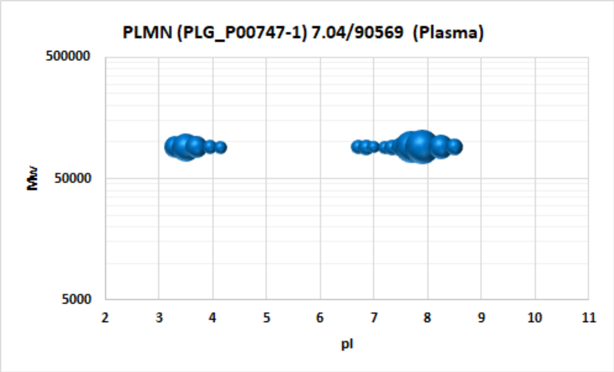	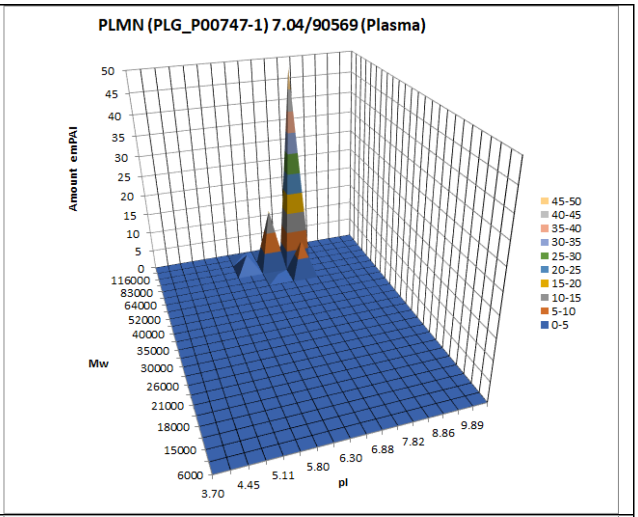
95.	PON1	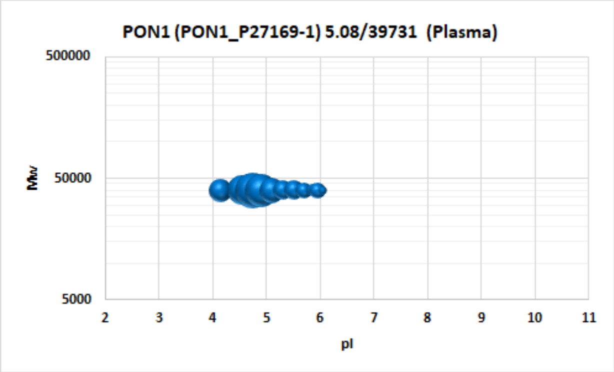	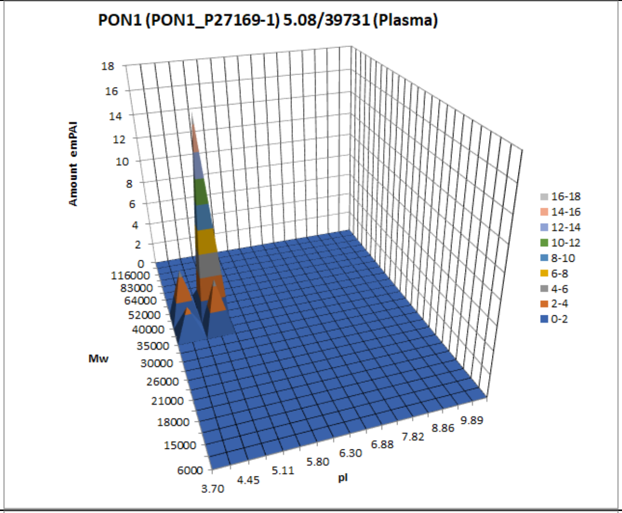
96.	PROP	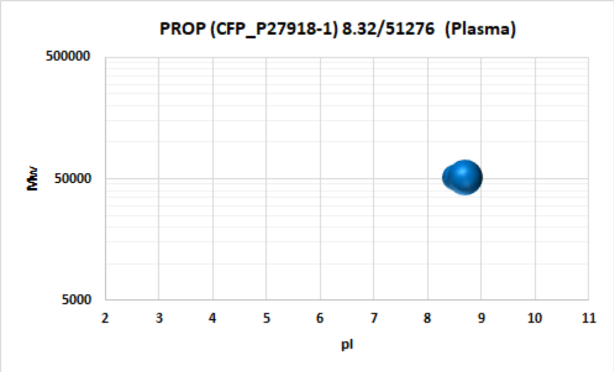	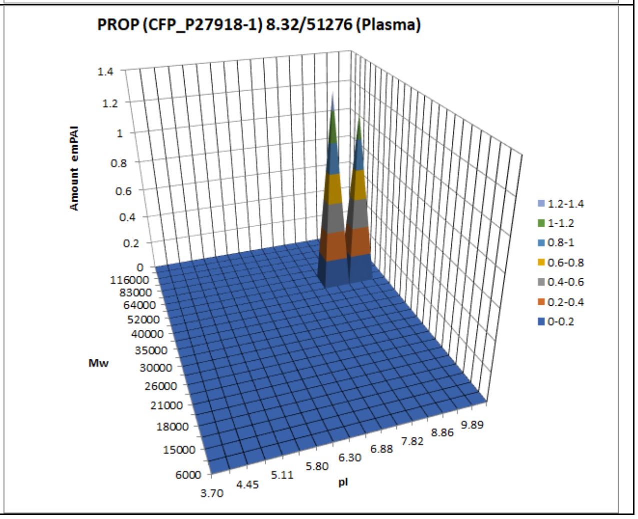

97.	PROS	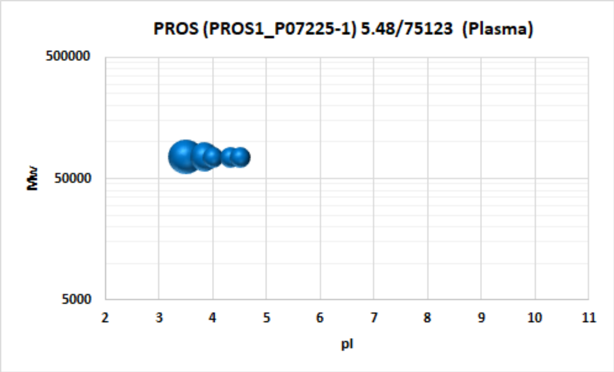	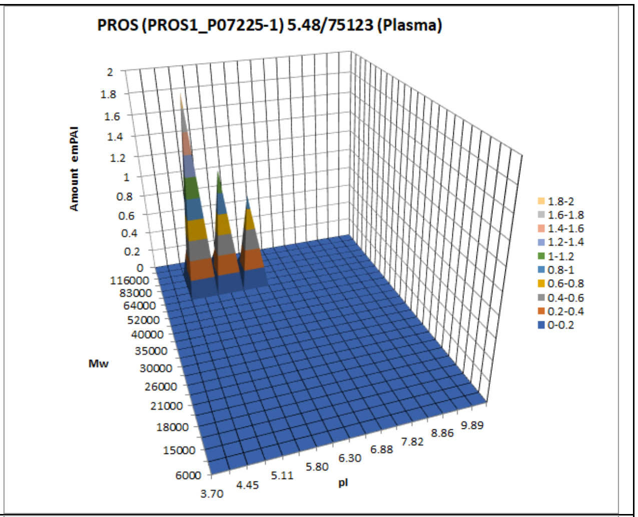
98.	RET4	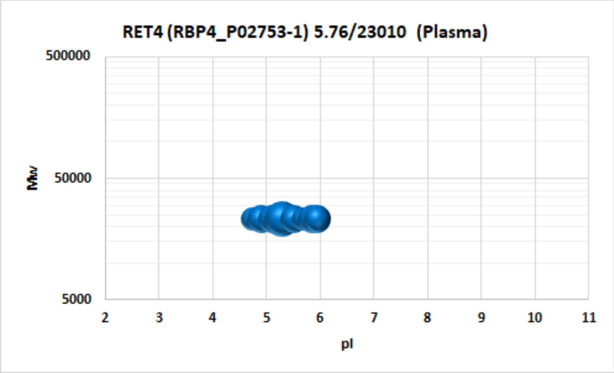	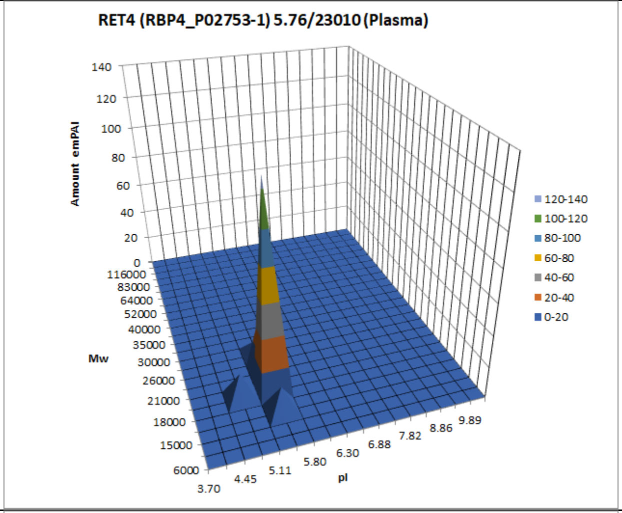
99.	SAA1	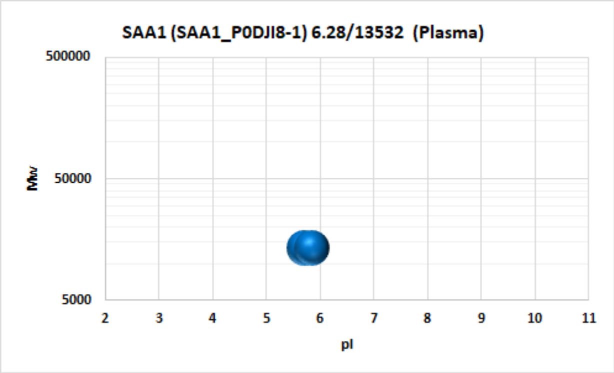	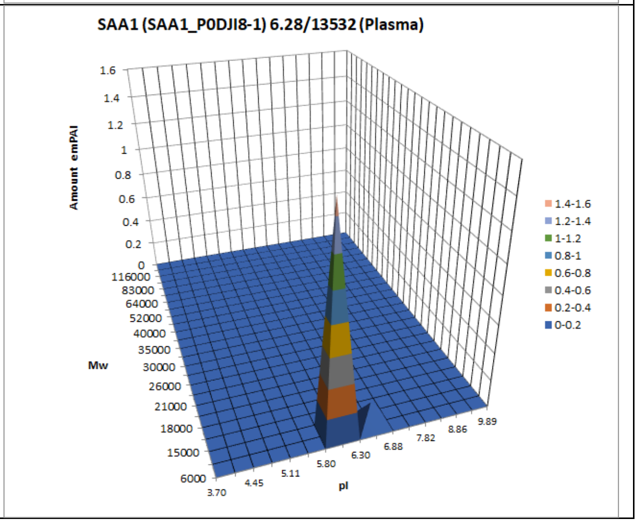

100.	SAA4	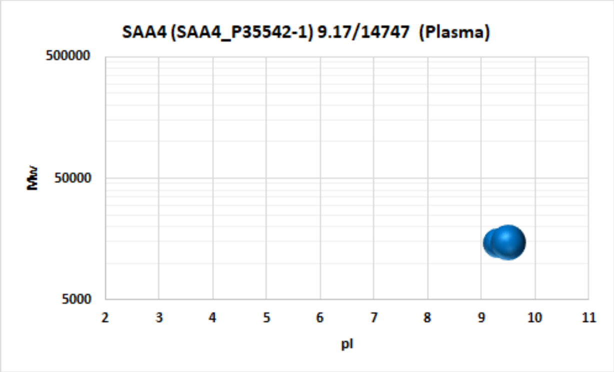	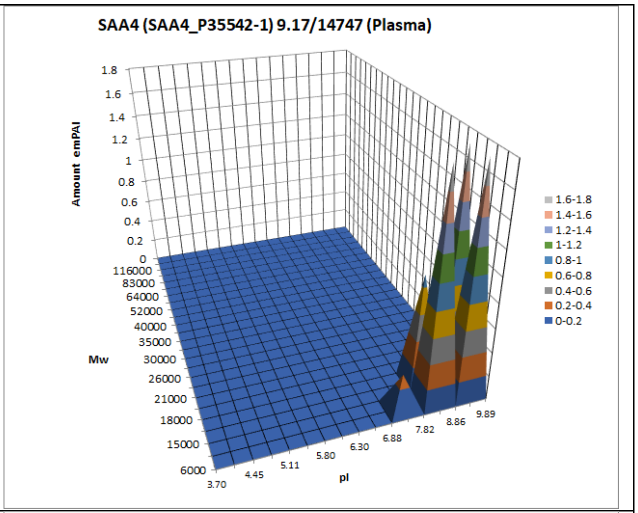
101.	SAMP	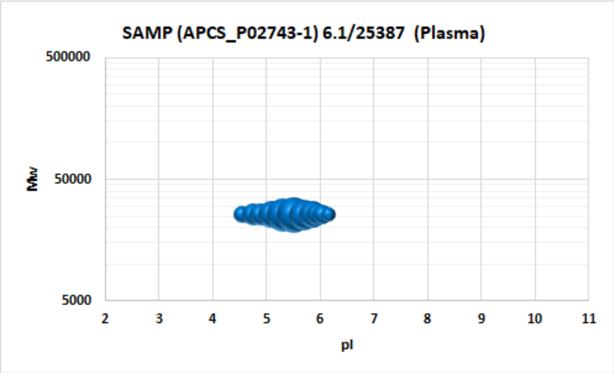	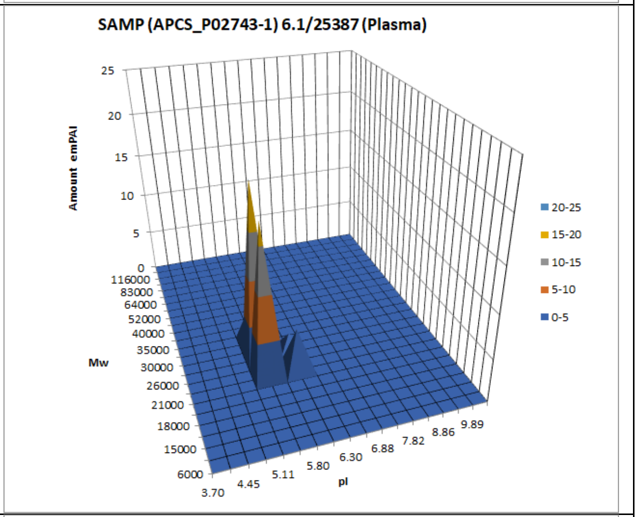
102.	SHBG	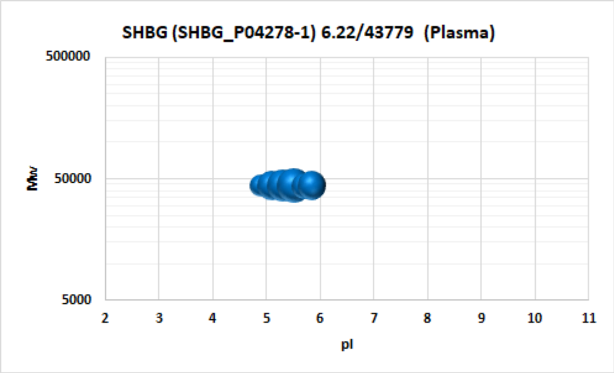	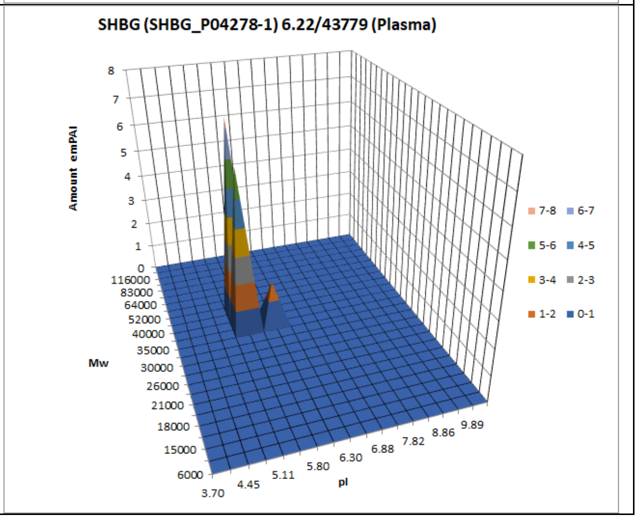

103.	S10A8	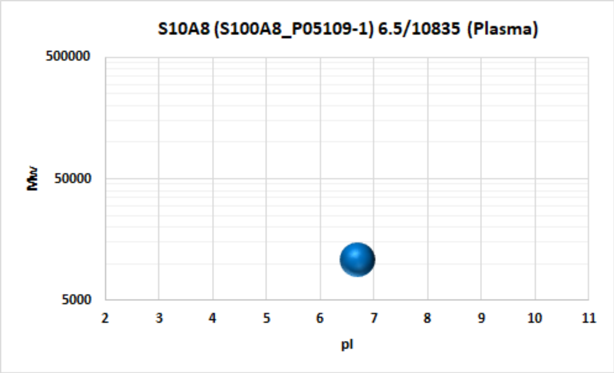	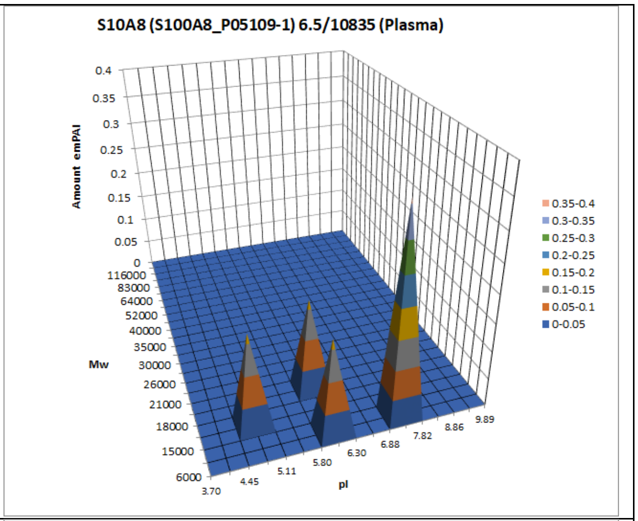
104.	S10A9	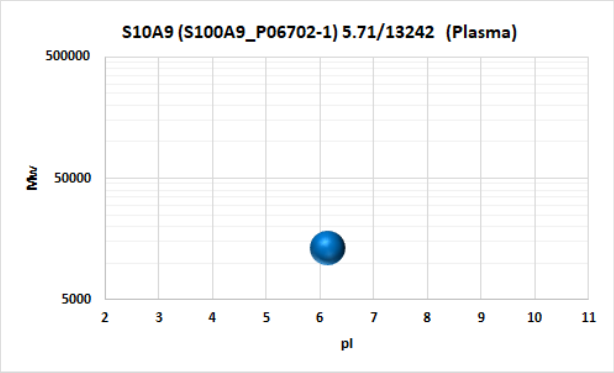	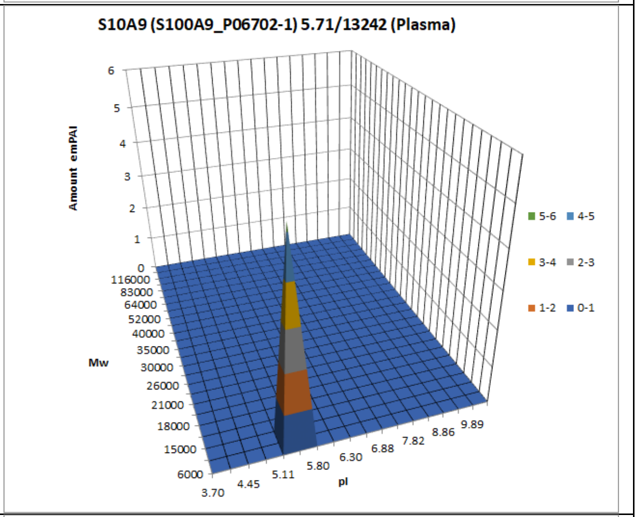
105.	TETN	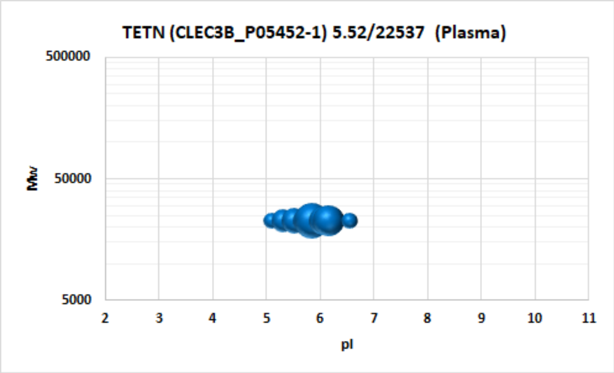	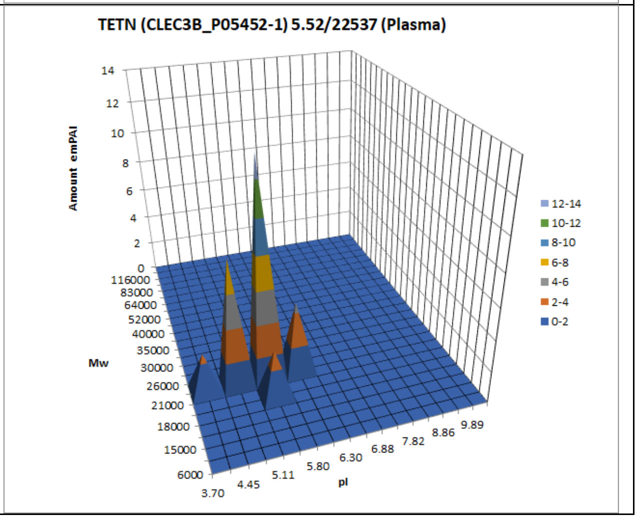

106.	THBG	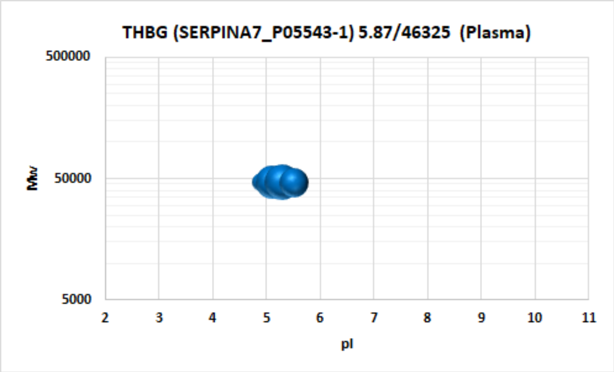	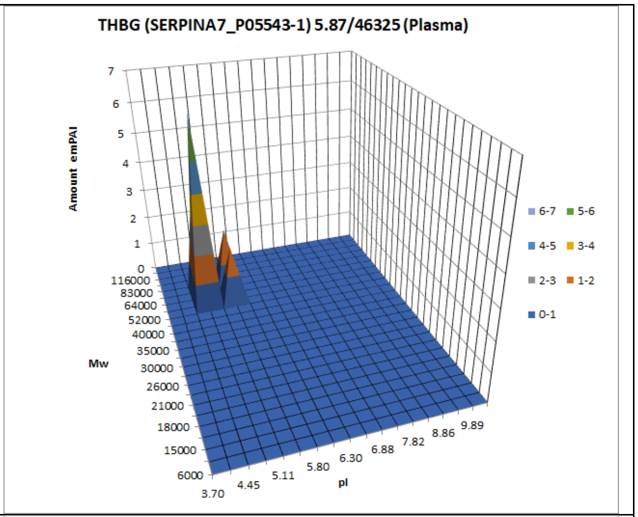
107.	THRB	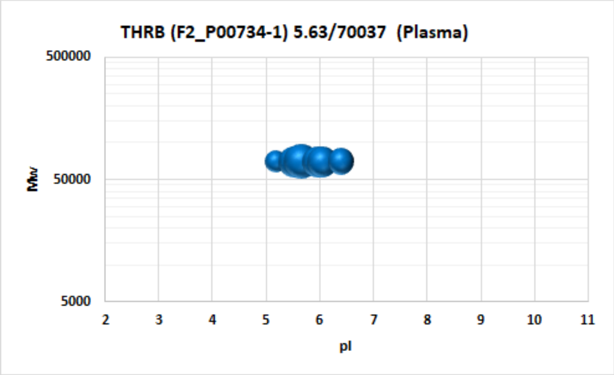	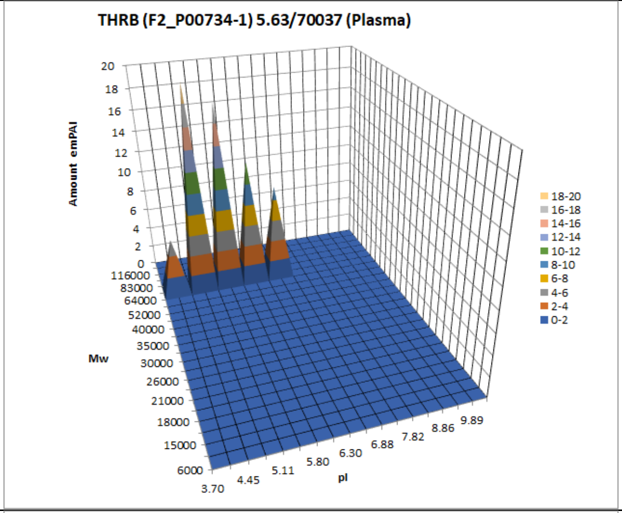
108.	TRFE	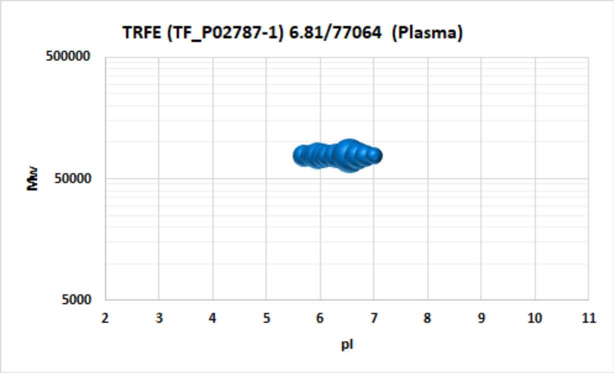	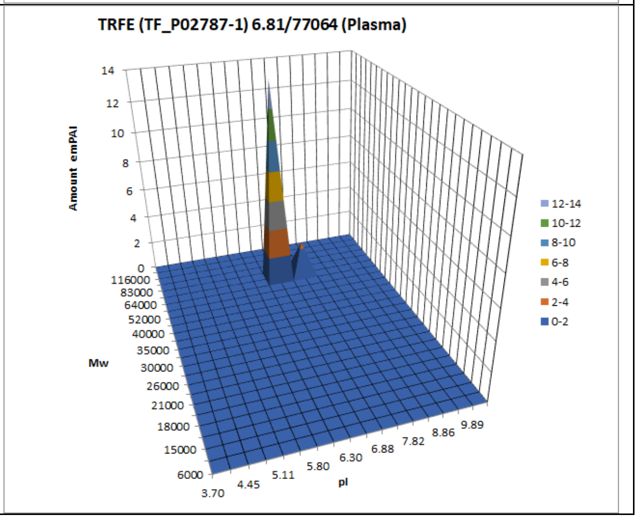

<table>
<tr>
<td>109.</td>
<td>TTHY</td>
<td>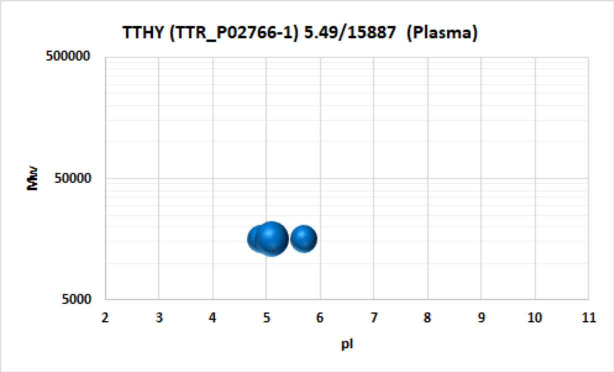
</td>
<td>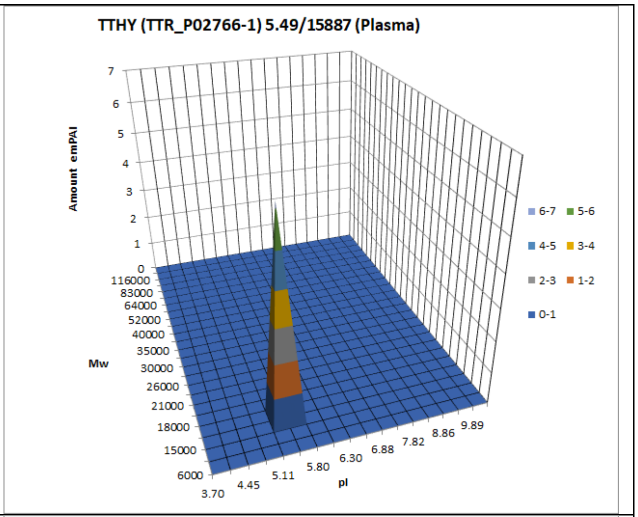
</td>
</tr>
<tr>
<td>110.</td>
<td>VTDB</td>
<td>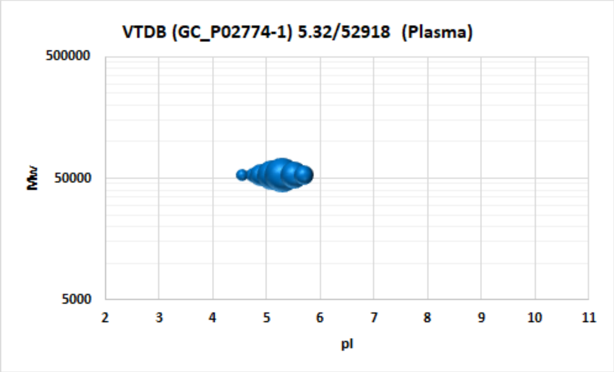
</td>
<td>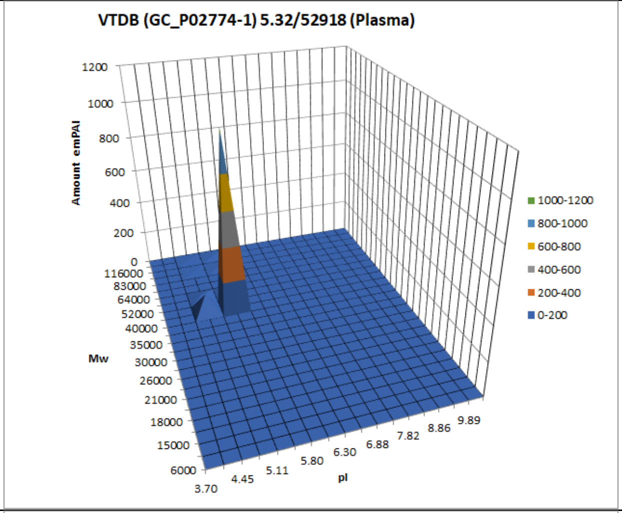
</td>
</tr>
<tr>
<td>111.</td>
<td>VTNC</td>
<td>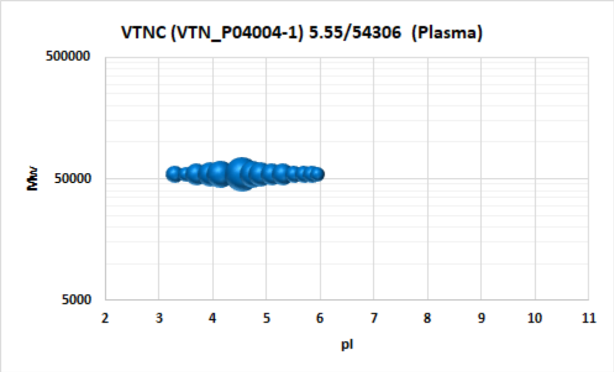
</td>
<td>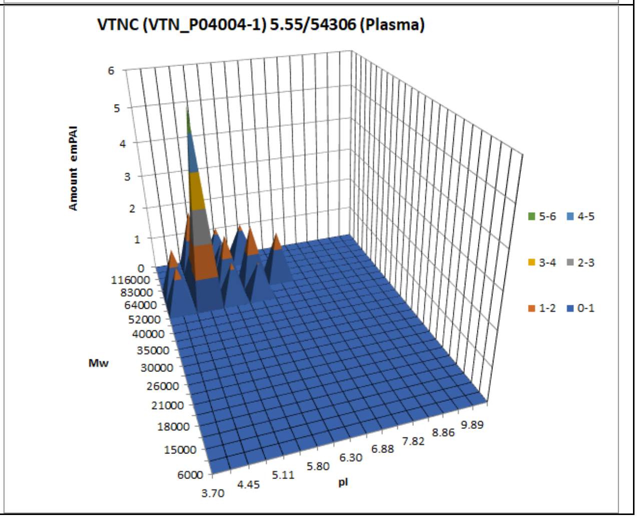
</td>
</tr>
</table>

112.	ZA2G	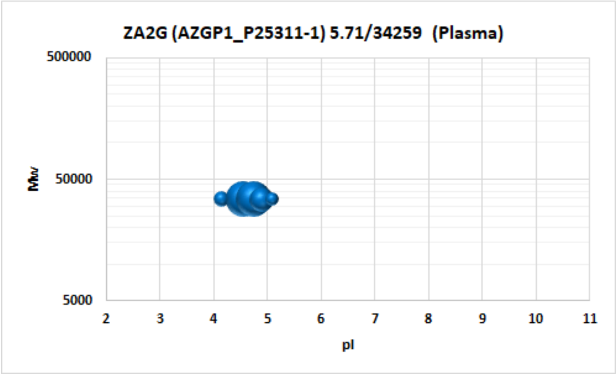ZA2G (AZGP1_P25311-1) 5.71/34259 (Plasma)<br>500000<br>50000<br>5000<br>Mw<br>2 3 4 5 6 7 8 9 10 11<br>pI	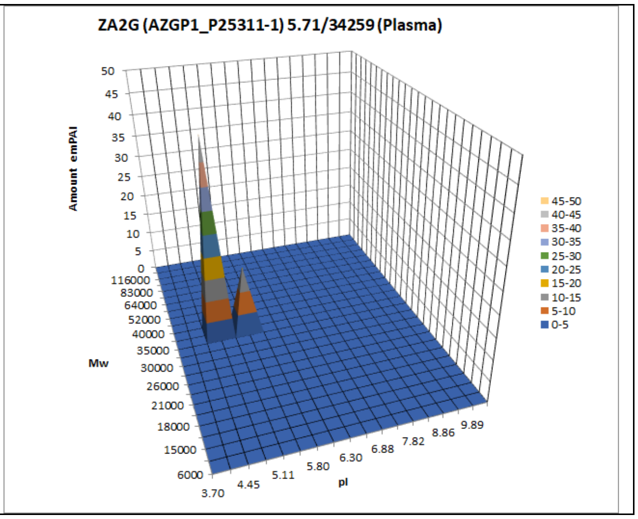ZA2G (AZGP1_P25311-1) 5.71/34259 (Plasma)<br>Amount emPAI<br>50 45 40 35 30 25 20 15 10 5 0<br>Mw<br>116000 83000 64000 52000 40000 35000 30000 26000 21000 18000 15000 6000<br>pI<br>3.70 4.45 5.11 5.80 6.30 6.88 7.82 8.86 9.89<br>45-50<br>40-45<br>35-40<br>30-35<br>25-30<br>20-25<br>15-20<br>10-15<br>5-10<br>0-5
